# Assessing *O*‑Naphthylmethyl and *O*‑Anthracenemethyl Glycosides as Metabolic Inhibitors of Bacterial Glycan Biosynthesis

**DOI:** 10.1021/acsinfecdis.5c00559

**Published:** 2025-09-22

**Authors:** Panhasith Ung, Ankita Paul, Soumyakanta Maji, Pilar Saavedra-Weis, Karen D. Moulton, Suvarn S. Kulkarni, Danielle H. Dube

**Affiliations:** † Department of Chemistry & Biochemistry, 2050 Bowdoin College, 2 Polar Loop, Brunswick, Maine 04011, United States; ‡ Department of Chemistry, 29491 Indian Institute of Technology Bombay, Powai, Mumbai 400-076, India

**Keywords:** glycan, azide, metabolic labeling, bioorthogonal chemistry, inhibitor

## Abstract

Bacterial glycans play a crucial role in survival and pathogenesis, making them attractive antibiotic targets. Unlike mammalian glycans, bacterial glycans incorporate rare sugars such as bacillosamine, *N*-acetylfucosamine, and 2,4-diacetamido-2,4,6-trideoxy galactose. To probe the role of bacterial glycans, we previously developed *O*-benzyl glycosides that metabolically inhibit *Helicobacter pylori* glycan biosynthesis and impair bacterial fitness. Here, we probed the efficacy of *O*-naphthylmethyl and *O*-anthracenemethyl glycosides, which bear larger aglycones relative to previously reported bacterial metabolic inhibitors. *O*-Naphthylmethyl d-*N*-acetylfucosamine inhibited *H. pylori* glycan biosynthesis, reduced biofilm formation, and impeded *H. pylori* growth at lower concentrations than its *O*-benzyl analog while leaving glycosylation of the commensal bacterium *Bacteroides fragilis* intact. By contrast, the *O*-anthracenemethyl glycosides tested were not effective metabolic glycan inhibitors. These metabolic inhibitors expand the bacterial glycoscience toolkit for probing protein glycosylation, help refine metabolic glycan inhibitor design parameters, and have the potential to set the stage for a glycan-based strategy to selectively target pathogens.

## Graphical abstract

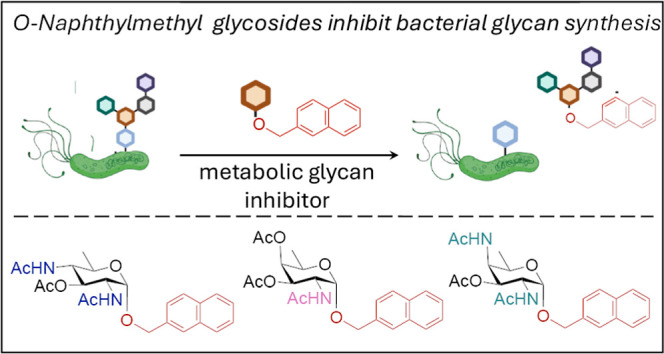

Bacteria encapsulate their cell surfaces with complex carbohydrate structures collectively known as the glycocalyx. The bacterial glycocalyx consists of capsular polysaccharide (CPS), lipopolysaccharide (LPS), peptidoglycan, and, for a subset of bacteria, glycoproteins.^1^ Collectively, cell envelope glycans play a pivotal role in bacterial fitness and survival. Among its essential functions, the glycocalyx facilitates intercellular communication,^2^ adhesion to host cells,^3^ and evasion of the host immune system.^4–6^ Some small molecule inhibitors that disrupt bacterial glycan biosynthesis and function, including penicillin,^7^ vancomycin,^8^ and polymyxin,^9^ have served as blockbuster antibiotics used to treat bacterial diseases. However, widespread misuse and overuse of these antibiotics have led to an alarming increase in antibiotic resistance among pathogenic bacteria, underscoring the urgent need for the development of novel therapeutic strategies.

Bacterial glycans exhibit remarkable compositional diversity. While mammalian glycans are composed of a limited set of nine monosaccharide building blocks, bacterial glycans are collectively composed of more than 700 exclusively bacterial monosaccharides.^10^ However, not all of these monosaccharides are utilized by all of the bacteria. Rather, monosaccharide usage varies among bacterial species and even serotypes of the same species. Some rare deoxy amino sugars appear to be uniquely expressed by specific pathogenic bacteria. Pathogen-associated monosaccharides include di-*N*-acetyl d-bacillosamine (d-Bac), d-2,4-diacetamido-2,4,6-trideoxy galactose (d-DATDG), and *N*-acetyl d-fucosamine (d-FucNAc) (Figure 1A, **1–3**), which are known to be present in higher-order glycans of *Helicobacter pylori*,^11,12^ *Campylobacter jejuni*,^13^ and *Pseudomonas aeruginosa*.^14^ These distinctive monosaccharides present attractive targets for the development of species-specific antibacterial therapeutics.

**Figure 1:**
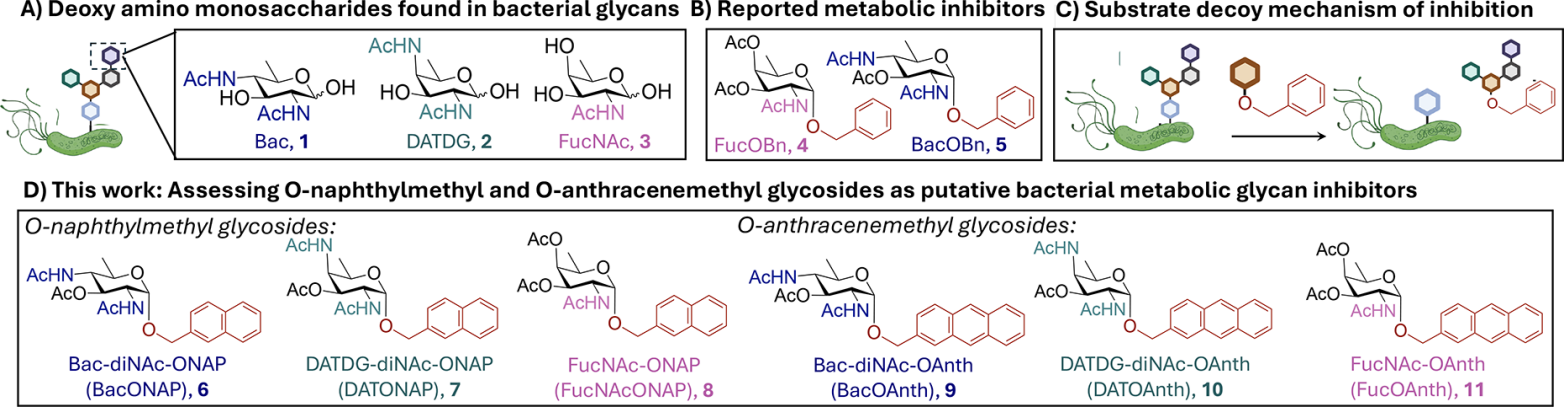
Exclusively bacterial rare deoxy amino monosaccharides serve as targets and scaffolds for metabolic inhibitors that disrupt bacterial glycan biosynthesis. (A) A sampling of exclusively bacterial deoxy amino sugars observed in glycans of bacterial pathogens. (B) Previously reported *O*-benzylglycoside inhibitors known to interfere with bacterial glycan biosynthesis. (C) Proposed mechanism of the substrate decoy metabolic inhibitor on bacterial glycan biosynthesis. (D) Novel *O*-naphthylmethyl and *O*-anthracenemethyl glycoside inhibitors introduced in this study as putative metabolic inhibitors designed to disrupt bacterial glycan biosynthesis.

While bacterial glycan structural diversity affords exciting prospects for antibiotic development, it introduces challenges for delineating glycan function. Genetic perturbation of glycosyltransferase-encoding genes leads to virulence attenuation and suggests a direct link between bacterial glycans and bacterial fitness. For instance, insertional inactivation of glycoprotein biosynthesis in *H. pylori* disrupts motility and biofilm formation,^15^ two factors important for colonization of the host. Moreover, genetic disruption of *C. jejuni* glycoprotein biosynthesis impairs bacterial growth and increases susceptibility to host proteases.^16^ Similarly, deletion of genes responsible for pilin glycosylation in *P. aeruginosa* suggest an essential role of glycans in virulence within the pulmonary environment.^14^ Despite these advances, genetic approaches often fail to isolate the effects of individual glycan structures. Small molecule inhibitors offer a complementary approach to dissecting glycan biosynthesis and function.

The development of bacterial glycosylation inhibitors has relied on both computational and experimental approaches, and in some cases, specific glycan biosynthesis enzymes were the targets of inhibitor screens. In 2017, Zhang et al. conducted an in silico screening of over 500,000 compounds to identify competitive inhibitors of glycosyltransferase C, a key enzyme in the biosynthesis of glycosyl polymers by *Streptococcus mutans*. Subsequent in vivo studies in bacterial cells confirmed efficacy in inhibiting biofilm formation.^17^ In the same year, Xu et al. screened nonsubstrate-like molecules, leading to the identification of a covalent inhibitor of LgtC, an α-1,4-galactosyltransferase involved in bacterial lipooligosaccharide biosynthesis.^18^ Razvi et al. utilized high-throughput screening to identify methyl 2-(2-pyridinylmethylene) hydrazinecarbodithioate and its phenyl derivative as potent, noncompetitive, and nontoxic inhibitors of *P. aeruginosa* exopolysaccharide biosynthesis, specifically targeting PelA and consequently impairing biofilm formation.^19^ In these examples, the identification of novel inhibitors relied on a comprehensive understanding of glycan-processing enzymes, their expression, and access to robust in vitro assays.

An alternative approach to developing bacterial glycosylation inhibitors is to focus on substrate-based metabolic inhibitors that can be screened directly in cell-based assays. In 2022, Morrison et al. demonstrated that C6-substituted UDP-GlcNAc acts as a chain terminator for the biosynthesis of *Escherichia coli*’s extracellular matrix polysaccharide poly-*N*-acetylglucosamine and reduces biofilm formation.^20^ Our laboratory previously developed *O*-benzyl^21^ and *S*-benzyl^22^ glycoside analogs of d-Bac and d-Fuc (Figure 1B, **4–5**) as metabolic glycan inhibitors that disrupt *H. pylori* glycoprotein biosynthesis and compromise bacterial fitness. These compounds are designed to act as substrate decoys that divert glycosyltransferase activity onto decoy substrates, leading to an accumulation of glycans on decoy substrates and a concomitant truncation of endogenous glycans (Figure 1C). Despite their bacteria-selective effects, the reported *O*- and *S*-glycosides exhibited only partial efficacy even at millimolar concentrations, necessitating the search for more effective glycoside analogs. In an extension of these studies, we were motivated to develop novel substrate decoys that more potently inhibit glycan biosynthesis in *H. pylori* and gain a better understanding of optimal design parameters for metabolic glycan inhibitors.

Here, we report the synthesis of novel *O*-naphthylmethyl (Figure 1D, **6–8**) and *O*-anthracenemethyl (Figure 1D, **9–11**) glycoside analogs of the rare deoxy amino bacterial sugars d-Bac, d-DATDG, and d-FucNAc. We further evaluated the efficacy of these compounds as metabolic glycan inhibitors in both pathogenic and commensal bacteria. The *O*-naphthylmethyl glycoside derivative FucNAcONAP (**8**) exhibited potent inhibition of glycan biosynthesis in *H. pylori*, demonstrating observable effects at concentrations lower than those of previously tested inhibitors. FucNAcONAP (**8**) induced multiple bacterial fitness defects, as evidenced by a significant reduction in biofilm formation and impaired growth. Notably, FucNAcONAP (**8**) did not affect glycan biosynthesis or fitness in the commensal bacterium *Bacteroides fragilis*, suggesting its selectivity. By contrast, the analogs of *O*-anthracenemethyl glycoside failed to exhibit significant inhibition of *H. pylori* glycan biosynthesis. Given that FucNAcONAP (**8**) effectively disrupts *H. pylori* protein glycosylation, it can serve as a chemical probe to investigate the functional role of glycoproteins in *H. pylori*.

## Results

### Design and Synthesis of *O*-Naphthylmethyl and *O*-Anthracenemethyl Glycosides

Inspired by the higher potency of *O*-naphthylmethyl glycosides relative to *O*-benzyl glycosides at inhibiting glycan biosynthesis in mammalian cells,^23^ we hypothesized that rare bacterial sugars functionalized as *O*-naphthylmethyl glycosides would demonstrate superior potency as bacterial glycan biosynthesis inhibitors compared to previously reported *O*-benzyl (*O*-Bn) analogs. Further, we wondered whether increasing the steric bulk of the aglycone from *O*-naphthylmethyl to *O*-anthracenemethyl glycosides would enhance potency of substrate decoys still further or whether steric factors would limit substrate tolerance of glycosylation enzymes. We designed derivatives of the rare bacterial monosaccharides d-Bac, d-DATDG, and d-FucNAc due to robust synthetic methodologies to access these scaffolds, their established utilization by select bacterial pathogens, and success of *O*-glycoside and *S*-glycoside metabolic inhibitors based on these sugars. As a first design element based on the previous use of *O*-benzyl glycosides to inhibit bacterial glycan biosynthesis,^21^ we reasoned that *O*-naphthylmethyl and *O*-anthracenemethyl glycosides would likely be recognized by the requisite glycosyltransferases as decoy substrates. As a second design element, we employed transient masking of hydrophilic hydroxyl groups on monosaccharide analogs with hydrophobic acetyl groups to facilitate uptake, as this approach has been successful in some bacteria.^12,24,25^ These design features parallel those adopted by Neelamegham and co-workers, who crafted the *N*-acetylglucosamine analog peracetylated *N*-acetylglucosamine-*O*-naphthylmethyl glycoside for their studies.^23^ Thus, we designed *O*-glycoside analogs BacONAP, DATONAP, FucNAcONAP, BacOAnth, DATOAnth, and FucOAnth (Figure 1D, **6–11**) that embody these two design criteria.

We synthesized the desired analogs **6–11** by adaptation of our previous approaches. Briefly, we relied on an efficient protocol for regioselective displacement of pyranosidic C-2, C-4 bistriflates with desired nucleophiles (azides or nitrites) to give access to a panel of functionalized rare sugar analogs including thioglycosides of bacillosamine, DAT, and fucosamine. Kulkarni and co-workers have developed an efficient, regioselective protocol for the displacement of C-2 and C-4 bistriflates with nucleophiles such as azides and nitriles, enabling access to a diverse array of functionalized rare sugar analogs, including thioglycosides of DAT, fucosamine, and bacillosamine.^21,26^ Preferential displacement at C-2 with bulky TBAN_3_ at −30 °C owing to steric and electronic effects, selectively retains the C-4 triflate, which can be subsequently displaced with NO_2_^–^ to install a hydroxyl group. Previously, this method allowed installation of OBn and SBn handles; here, we expand the glycoside library to include *O*-naphthylmethyl (**6**–**8**) and *O*-anthracenemethyl (**9**–**11**) analogs.

Electrophilic aromatic substitution (EAS) reactions are well-documented in polyaromatic scaffolds such as naphthalene and anthracene owing to their high electron density and extended conjugation.^27^ In our study, we have also observed this tendency during attempts to directly activate DAT thioglycoside **14** using *N*-iodosuccinimide (NIS), where unintended EAS pathways favored glycosyl bond formation. Therefore, for synthesis of the Bac-ONAP analog **16**, we converted Bac-thioglycoside **13** to a hemiacetal, followed by transformation to its subsequent trichloroacetimidate donor (Scheme 1). Glycosylation with commercially available naphthylmethyl alcohol in the presence of TfOH at 0 °C afforded the Bac-ONAP derivative **16** in 50% yield over three steps. The formation of the desired product **16** was supported by the AB quartet at δ 4.97, 4.86 ppm (*J* = 12.0 Hz, 2H) in ^1^H NMR, along with an inverted signal at δ 69.85 ppm observed in ^13^C-DEPT-135 NMR corresponding to the methylene group of the naphthylmethyl unit. Subsequent reduction of the diazido (Zn, AcOH) and acetylation furnished the DAT-ONAP analog **6** in 63% yield over two steps. Analogous glycosylation and azide-to-acetamide transformations provided DAT-ONAP **7** and FucNAc-ONAP **8** in good overall yields.

**Scheme 1:**
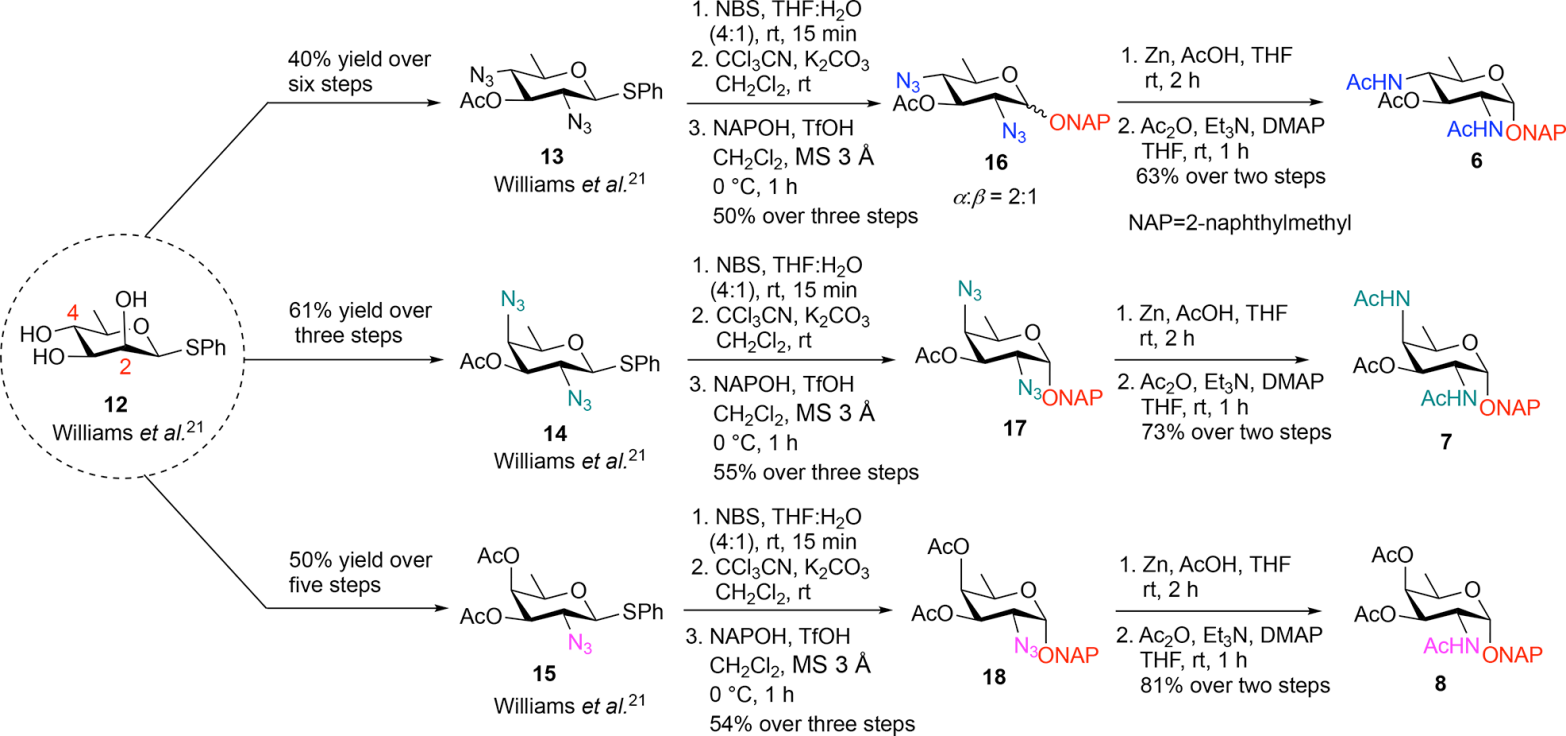
Synthesis of 2-Naphthylmethyl Analogs of BacONAP **6**, DATONAP **7**, and FucNAcONAP **8**

As a part of the synthesis of anthracenemethyl analogs, the thioglycoside donor **13** was hydrolyzed and converted to the Schmidt trichloroacetimidate, which upon glycosylation with 9-anthracenemethanol (AnthOH) exclusively in MeCN owing to its enhanced solubility^28,29^ gave the OAnth-coupled product **19** (α/β = 1:12) in 74% yield over three steps. Product **19** formation was confirmed by an AB quartet at δ 5.85, 5.79 ppm (*J* = 12.0 Hz, 2H) in ^1^H NMR and an inverted signal at δ 62.50 ppm in ^13^C-DEPT-135, indicating the CH_2_ of the anthracenemethyl group. Under identical conditions, the DAT donor **14** and Fuc donor **15** were converted to their corresponding OAnth-coupled product **20** (pure β) and **21** (α/β = 1:11) in 76% and 71% yield, respectively. Finally, azido groups of compounds **20** and **21** were converted into corresponding amine using Zn, AcOH, and subsequent masking of amines with an acetamido groups using Ac_2_O gave the desired target molecules **10** (pure β) and **11** (α/β = 1:15) in 81% and 75% yield, respectively. However, the reduction of azido groups in compound **19** posed significant challenges, potentially as both azido groups occupy equatorial positions at C2 and C4. After extensive optimization (Scheme 2, Table 1),^30^ the desired diamine was efficiently obtained using PPh_3_ with pyridine in THF/H_2_O (9:1), and subsequent acetylation with Ac_2_O in pyridine furnished compound **11** (α/β = 1:14) in 74% yield over two steps.

**Scheme 2:**
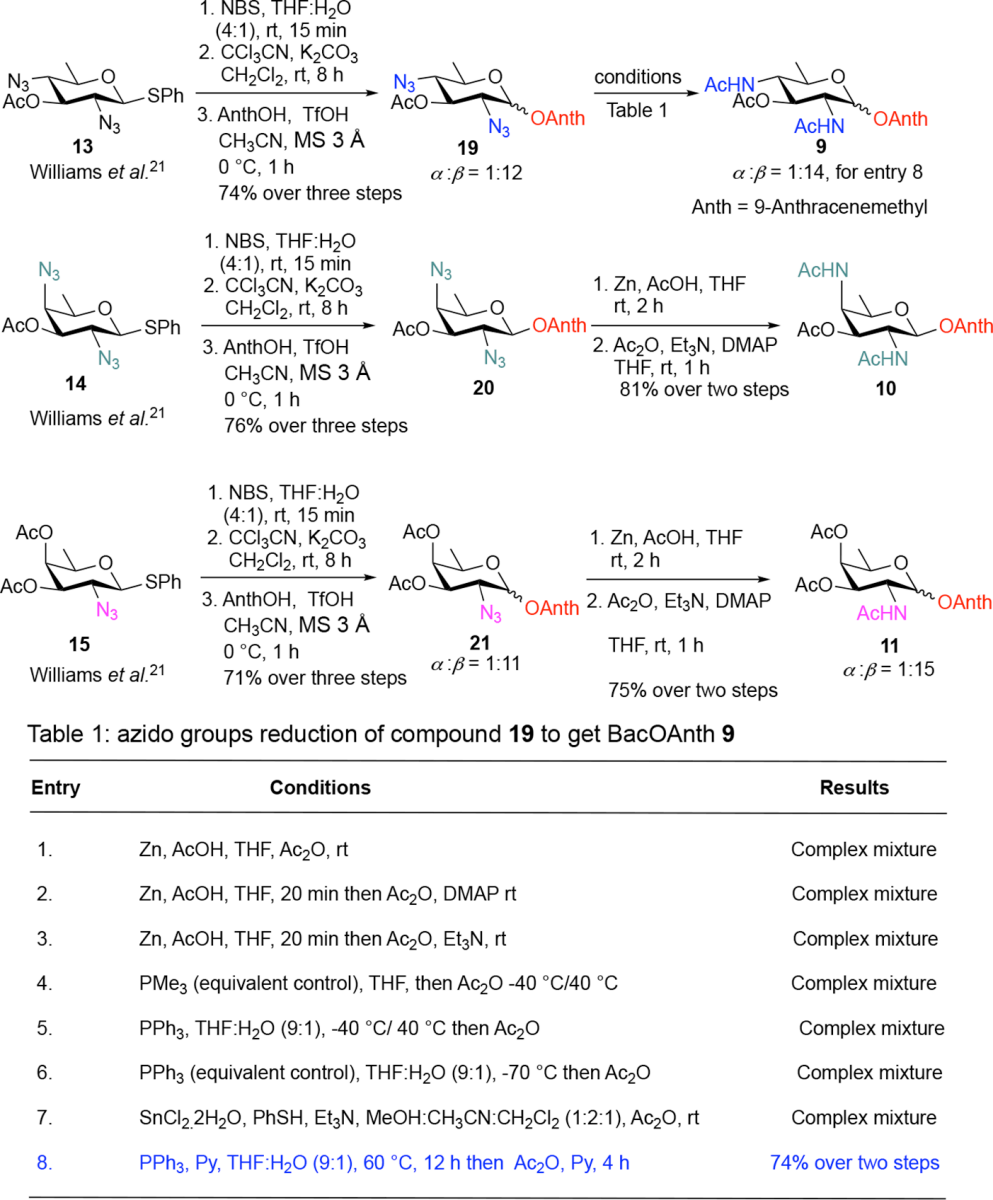
Synthesis of 9-Anthracenemethyl Analogs of BacOAnth **9**, DATOAnth **10**, and FucOAnth **11**

### *O*-Naphthylmethyl and *O*-Anthracenemethyl Glycosides Inhibit *H. pylori*’s Glycoprotein Biosynthesis

We began by investigating the inhibitory effects of *O*-naphthylmethyl and *O*-anthracenemethyl glycosides on *H. pylori* glycan biosynthesis. Toward this end, we employed our previously reported metabolic oligosaccharide engineering (MOE) cell-based assay to investigate bacterial glycan biosynthesis.^21^ This approach employs azide-containing monosaccharide analogs, which can be taken up and metabolized by bacterial cells. Upon incorporation into bacterial glycans, the azide moiety enables selective bioorthogonal labeling via Staudinger ligation with phosphine probes.^31^ Given previous success using peracetylated *N*-azidoacetyl glucosamine (Ac_4_GlcNAz) to metabolically label *H. pylori*’s suite of general O-linked glycoproteins,^24,25^ we leveraged metabolic labeling with Ac_4_GlcNAz as a method to detect glycoprotein biosynthesis and, when applicable, concomitant inhibition. Metabolic labeling was conducted using azide-containing Ac_4_GlcNAz as a positive control and the azide-free sugar peracetylated *N*-acetylglucosamine (Ac_4_GlcNAc) as a negative control. For experimental samples, *H. pylori* were cocultured with 0.5 mM Ac_4_GlcNAz and varying concentrations (0.25–1.0 mM or 0.5–2.0 mM) of each inhibitor (**6–11**) for 3 days. After metabolic labeling, bacterial cells were lysed in detergent-containing lysis buffer to extract proteins, which were subsequently subjected to Staudinger ligation with phosphine–FLAG (Phos-FLAG) to detect azide-labeled glycoproteins.^32,33^ Western blot analysis using an anti-FLAG antibody was then used to detect levels of glycoprotein biosynthesis.

Samples treated with the negative control Ac_4_GlcNAc (Ac) exhibited minimal signal, and the small amount of the observed signal corresponds to nonazide-labeled glycoproteins (Figure 2A). By contrast, a strong signal was observed at an array of molecular weights in samples treated with Ac_4_GlcNAz (Az) (Figure 2A) that corresponds to robust azide-labeled glycoprotein biosynthesis, as observed in previous reports.^24^ These signals benchmark the lowest and highest signals expected across experimental samples. Strikingly, *O*-anthracenemethyl glycosides **9–11** did not exhibit a potent inhibitory effect, with only slight inhibition observed at 2.0 mM for all analogs (Figure 2A). Therefore, we decided not to pursue these compounds in further experiments. Experimental samples treated with **6–8** exhibited a concentration-dependent inhibition of glycan biosynthesis. Specifically, BacONAP (**6**) inhibited glycan biosynthesis at 1.0 mM, DATONAP (**7**) at 0.5 mM, and FucNAcONAP (**8**) demonstrated the highest inhibitory activity, with substantial inhibition of glycoprotein biosynthesis upon treatment with 0.25 mM FucNAcONAP (**8**) (Figure 2A). Given the relative potency of FucNAcONAP (**8**), we sought to identify the threshold inhibitory concentration that could impact *H. pylori* glycoprotein biosynthesis. Toward this end, *H. pylori* were treated with 0.01, 0.1, or 0.25 mM FucNAcONAP (**8**) alongside 0.5 mM Ac_4_GlcNAz to score glycoprotein biosynthesis. Significant inhibition of *H. pylori* glycoprotein biosynthesis was observed at 0.1 and 0.25 mM FucNAcONAP (**8**) treatment (Figure 2A). To assess the relative potency of FucNAcONAP (**8**) with the previously reported *O*-benzyl glycoside FucOBn (**4**), *H. pylori* glycoprotein biosynthesis was assessed following treatment with 0.1–1.0 mM concentrations of these compounds. This head-to-head comparison revealed that FucNAcONAP (**8**) is more potent than the corresponding *O*-benzyl glycoside FucOBn (**4**) (Figure S1). Coomassie staining of these treated samples revealed consistent protein levels across samples and conditions (Figures S1 and S2A), indicating that differences observed by Western blot analysis were not due to uneven protein concentrations or sample loading. Taken together, these data show modest inhibitory activity for novel *O*-naphthylmethyl glycosides **6–8** and reveal FucNAcONAP (**8**) as the most potent inhibitor. By contrast, the *O*-anthracenemethyl glycosides **9–11** were not effective metabolic inhibitors.

**Figure 2:**
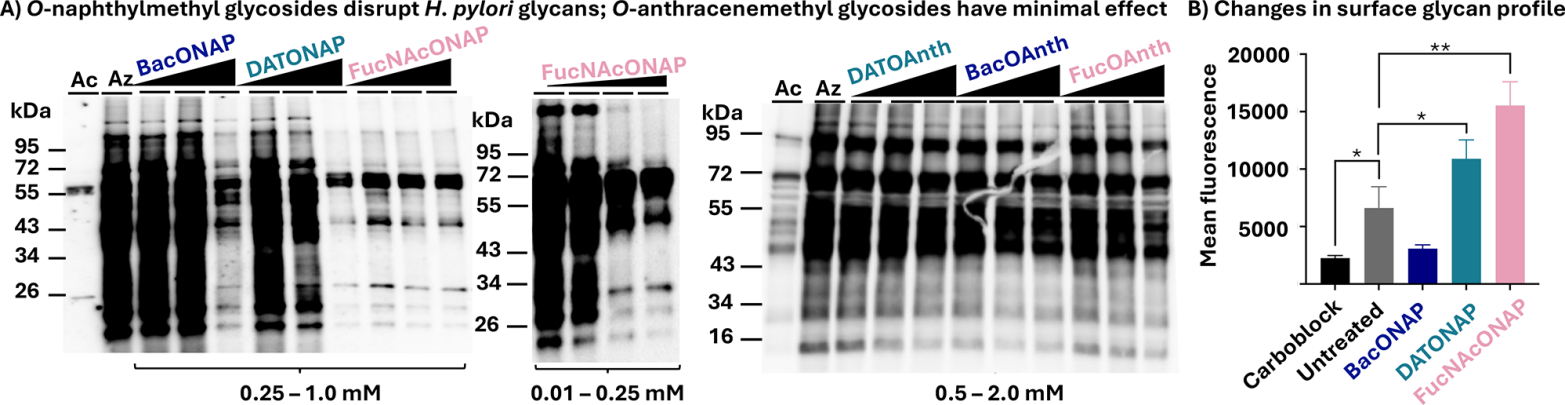
Inhibition of *H. pylori* glycan biosynthesis and surface glycan profile disruption by *O*-naphthylmethyl and *O*-anthracenemethyl glycosides **6–11**. (A) Western blot analysis indicates reduction in glycoprotein biosynthesis in *H. pylori* upon treatment with Ac_4_GlcNAz (0.5 mM) alongside increasing concentrations (0.25 mM, 0.5 mM, or 1.0 mM) of BacONAP (**6**), DATONAP (**7**), and FucNAcONAP (**8**) or (0.5 mM, 1.0 mM, or 2.0 mM) of BacOAnth (**9**), DATOAnth (**10**), and FucOAnth (**11**). Additionally, FucNAcONAP (**8**) was further evaluated at lower concentrations (0.01 mM, 0.05 mM, 0.1 mM, or 0.25 mM) to assess its inhibitory effects on glycan biosynthesis. Ac_4_GlcNAz (Az) treatment with no inhibitor addition served as a positive control, and treatment with the azide-free sugar Ac_4_GlcNAc (Ac) served as a negative control. (B) Flow cytometry analysis demonstrated a change in fluorescence intensity of ConA upon treatment with 1 mM BacONAP (**6**), 1 mM DATONAP (**7**), and 0.1 mM FucNAcONAP (**8**) compared to untreated samples, consistent with the perturbed cell surface glycan profile. ConA pretreatment with 400 mM mannose (carboblock) prior to probing untreated *H. pylori* led to decreased binding. Error bars represent the technical replicates. Data are representative of replicate experiments (*n* = 3) that exhibited the same findings. One-sample *t*-test was used to determine statistical significance between control and experimental samples (*P < 0.01; **P < 0.001).

To further probe the effects of novel glycosides on glycan biosynthesis, we performed a lectin-binding assay to measure surface glycan perturbation as a complementary assay. The mannose-binding lectin *concanavalin A* (ConA) was used to measure *H. pylori* cellular glycan architecture according to previous reports.^21^ Briefly, *H. pylori* were treated with either 1.0 mM BacONAP (**6**), 1.0 mM DATONAP (**7**), or 0.1 mM FucNAcONAP (**8**) or without a putative glycan inhibitor and then probed with AlexaFluor 488-ConA to analyze glycan architecture. As a control, ConA was preincubated with its endogenous ligand mannose before being applied to *H. pylori*. Flow cytometry analysis revealed robust fluorescence of untreated cells following incubation with ConA and suppressed fluorescence when untreated cells were incubated with ConA pretreated with mannose (“carboblock”), thus validating the assay (Figure 2B). A significant change in fluorescence was observed in *H. pylori* treated with 1 mM DATONAP (**7**) and 0.1 mM FucNAcONAP (**8**) relative to untreated cells (Figures 2B and S2B), indicating alterations in the surface glycan profile aligning with previous studies.^15,20,21^ The diminished level of lectin binding observed upon treatment with 1 mM BacONAP (**6**) suggests the surface glycan profile is perturbed differently, perhaps by a reduction in relative levels of mannose-containing glycans on *H. pylori*.

We wondered whether alterations in the cell surface glycan profile detected via lectin-binding were due to inhibition of glycoprotein biosynthesis alone or coupled to inhibition of LPS biosynthesis as well. To determine whether FucNAcONAP (**8**) affects LPS biosynthesis, we performed a crude preparation of LPS from untreated *H. pylori* and *H. pylori* treated with 0.25 mM FucNAcONAP (**8**). Briefly, *H. pylori* were lysed in detergent, and resulting pellets and supernatant were boiled in LPS lysis buffer containing SDS and β-mercaptoethanol. After heating, samples were treated with proteinase K overnight and then boiled prior to electrophoretic analysis. Visualization of LPS with Pro-Q Emerald 300 polysaccharide stain revealed *H. pylori* treated with 0.25 mM FucNAcONAP (**8**) exhibited a signal corresponding to high-molecular-weight LPS bearing O-antigens (4–50 kDa^34^), comparable to LPS produced by untreated controls (Figure S2C). These data suggest that FucNAcONAP (**8**) does not appreciably interfere with LPS biosynthesis in *H. pylori*. This finding supports the hypothesis that the inhibitory effects of FucNAcONAP (**8**) arise specifically from the disruption of glycoprotein biosynthesis. Previous genetic perturbation studies indicate that glycoprotein and LPS biosynthesis pathways in *H. pylori* overlap at early stages and bifurcate at a later stage, with further tailoring of glycoprotein structures following this bifurcation event.^15^ FucNAcONAP (**8**) may affect only glycoprotein biosynthesis by interfering with dedicated glycoprotein biosynthesis tailoring enzymes that occur following glycoprotein and LPS pathway divergence.

### *O*-Naphthylmethyl Glycosides Alter *H. pylori*’s Growth, Motility, and Biofilm Formation to Varying Extents

Having established that *O*-naphthylmethyl glycosides inhibit glycoprotein biosynthesis and alter glycan architecture of *H. pylori*, we next sought to determine whether changes in glycan biosynthesis and presentation translate to functional defects in *H. pylori* physiology, particularly in processes essential for host colonization. Thus, growth, motility, and biofilm formation of *H. pylori* treated with *O*-naphthylmethyl glycosides was compared to these phenotypes in untreated *H. pylori*. To begin, we scored growth over time by monitoring optical density at 600 nm (OD_600_) daily for *H. pylori* treated with the lowest concentrations of the compound required to inhibit glycoprotein biosynthesis. Thus, we treated *H. pylori* with 1.0 mM BacONAP (**6**), 1.0 mM DATONAP (**7**), and 0.1 mM FucNAcONAP (**8**). Untreated *H. pylori* exhibited early exponential growth and reached the stationary phase after 2 days (Figure 3A). Treatment with 1.0 mM BacONAP (**6**) and 0.1 mM FucNAcONAP (**8**) caused an initial delay in the exponential phase but had no significant impact on achieving stationary-phase growth comparable to that of untreated cells. Treatment with 1.0 DATONAP (**7**) elicited strong growth inhibition, with no appreciable increase in OD_600_ observed over time. To determine whether the observed growth effects were due to compound toxicity, viability was scored using live/dead staining, followed by flow cytometry analysis (Figure S3). DATONAP (**7**) and FucNAcONAP (**8**) exhibited slight toxicity, suggesting that their inhibitory effects may be partially attributed to cytotoxicity.

**Figure 3:**
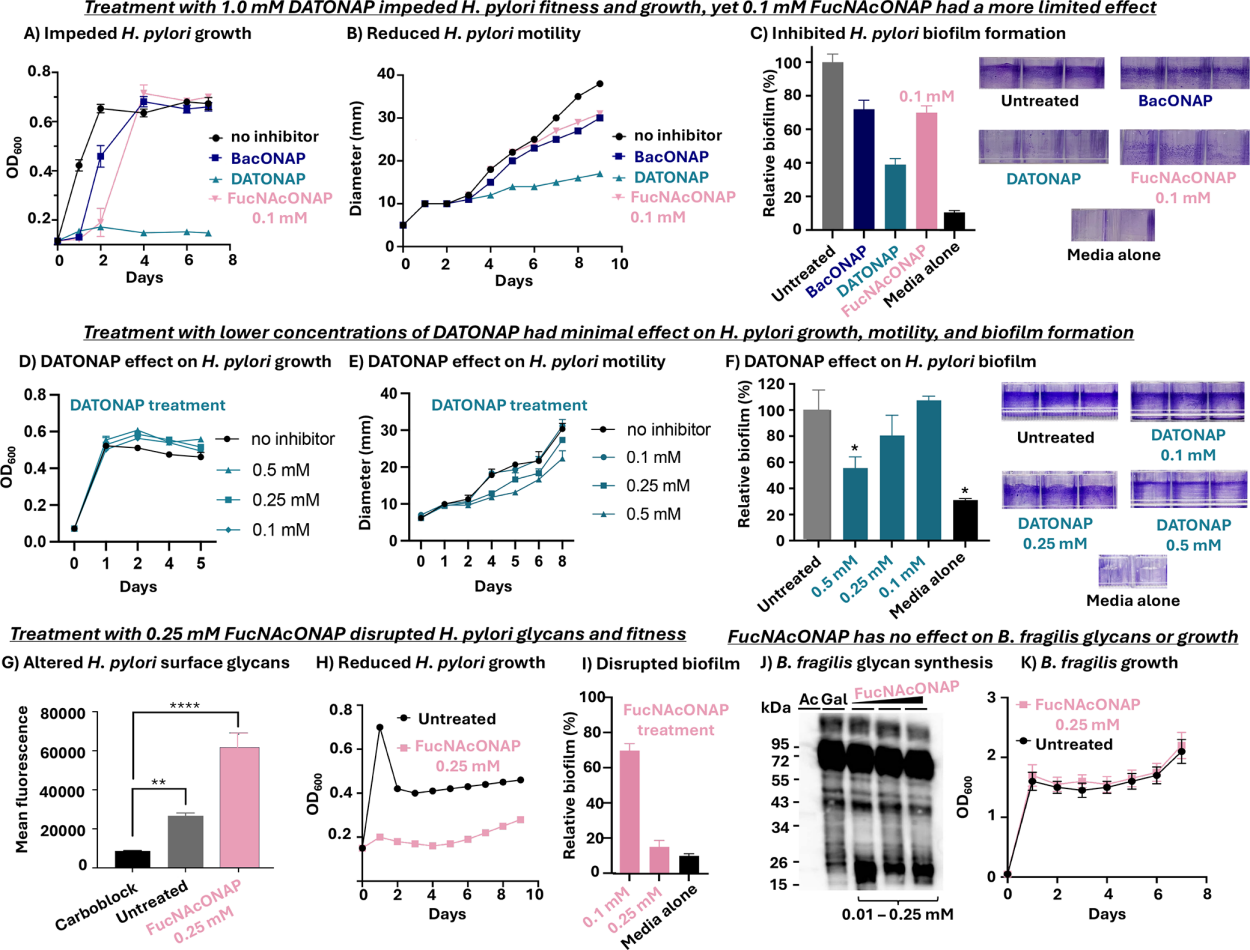
*O*-Naphthylmethyl glycosides have a modest effect on *H. pylori* fitness yet have no apparent effect on *B. fragilis* glycan biosynthesis nor growth. (A–C) Treatment with 1.0 mM DATONAP impeded *H. pylori* fitness and growth, whereas 0.1 mM FucNAcONAP had a more limited effect. (A) Growth of *H. pylori* was monitored following treatment with 1.0 mM BacONAP (**6**), 1.0 mM DATONAP (**7**), or 0.1 mM FucNAcONAP (**8**). (B) *H. pylori* motility was monitored on soft agar and was reduced upon treatment with 1.0 mM BacONAP (**6**), 1.0 mM DATONAP (**7**), and 0.1 mM FucNAcONAP (**8**) compared to the untreated control. (C) Treatment with *O*-naphthylmethyl glycosides reduced percent biofilm formation relative to untreated *H. pylori* cells, as quantified via release of bound crystal violet with 30% acetic acid and measuring absorbance at 562 nm. (D–F) DATONAP has minimal effects on *H. pylori* fitness at low concentrations. (D) DATONAP treatment at low concentrations (0.1, 0.25, 0.5 mM) had no suppressive effect on *H. pylori* growth. (E) Treatment of *H. pylori* with 0.25 mM or 0.5 mM DATONAP slowed the motility of the bacteria relative to no inhibitor treated bacteria, yet treatment with 0.1 mM DATONAP had no effect on motility. (F) Treatment with 0.25 mM or 0.1 mM DATONAP had no significant effect on *H. pylori* biofilm formation relative to untreated controls. (G–I) *H. pylori* fitness assays measuring the effects of 0.25 mM FucNAcONAP revealed an altered surface glycan profile, biofilm formation, and growth. (G) Treatment with 0.25 mM FucNAcONAP disrupted the *H. pylori* surface glycan profile, as indicated by an increase in ConA fluorescence intensity compared to untreated samples. (H) Bacterial growth was reduced in the presence of 0.25 mM FucNAcONAP. (I) Treatment with 0.25 mM FucNAcONAP (**8**) reduced percent biofilm formation relative to untreated *H. pylori* cells, as quantified via release of bound crystal violet. (J,K) Treatment of *B. fragilis* with 0.25 mM FucNAcONAP had minimal effect on *B. fragilis* glycans and growth. (J) Western blot analysis showed that the glycoprotein profile of *B. fragilis* remained unchanged upon treatment with increasing concentrations of FucNAcONAP (**8**) (0.01 mM–0.25 mM), comparable to the untreated control, indicating no appreciable effect on glycan biosynthesis. (K) Moreover, monitoring *B. fragilis* growth over time demonstrated that treatment with 0.25 mM FucNAcONAP (**8**) did not interfere with *B. fragilis* growth, supporting the selectivity of FucNAcONAP (**8**) as a metabolic inhibitor. Error bars represent the technical replicates. The data shown are representative of replicate experiments (*n* = 3) that exhibited the same findings. One-sample *t*-test was used to determine statistical significance between control and experimental samples (**P < 0.001; ****P < 0.00001).

An established assay was used to score the effect of *O*-naphthylmethyl glycosides on *H. pylori* motility on soft agar.^35^ Briefly, inhibitor-treated bacteria were plated on soft agar, and motility was quantified by measuring the increase in colony diameter over a 10 day period. Compared to the untreated negative control, treatment with BacONAP (**6**), DATONAP (**7**), and FucNAcONAP (**8**) resulted in a significant reduction in *H. pylori* motility, with 1.0 mM DATONAP (**7**) treatment causing the most pronounced motility defect (Figure 3B). Furthermore, the impact of *O*-naphthylmethyl glycosides on *H. pylori* biofilm formation was assessed using a crystal violet assay (Figure 3C). Briefly, biofilms were stained with crystal violet, and biofilm formation was quantified by dissolving the bound dye with acetic acid followed by absorbance measurements (Figure 3C).^36^ Relative to untreated controls, BacONAP (**6**), DATONAP (**7**), and FucNAcONAP (**8**) treatment significantly reduced the level of biofilm formation (Figure 3C). Given the role of the flagellar filament in early stages of biofilm formation, these metabolic glycan inhibitors may disrupt biofilm formation by interfering with flagellar assembly.

Treatment with 1.0 mM DATONAP led to significant impacts on growth and the corresponding fitness attributes (Figure 3A–C). However, lower concentrations of DATONAP (0.1, 0.25, and 0.5 mM) had minimal effect on *H. pylori* fitness relative to no inhibitor treated controls (Figure 3D–F). Modest fitness effects at low concentrations of DATONAP are consistent with the minimal effects of low concentrations of DATONAP on *H. pylori* glycoprotein biosynthesis (Figure 2A). Though 0.1 mM FucNAcONAP (**8**) treatment subtly inhibited glycoprotein biosynthesis and impacted fitness, we were curious about whether this concentration might be near a threshold activity level that, if boosted slightly, might precipitate more substantial fitness effects. Indeed, increasing the FucNAcONAP (**8**) concentration to 0.25 mM resulted in altered surface glycans (Figure 3G) and a significant reduction in both bacterial growth and biofilm formation compared to untreated cells (Figure 3H,I). These findings suggest that 0.1 mM FucNAcONAP (**8**) is near a borderline concentration for inhibiting *H. pylori* glycoprotein biosynthesis, and a higher concentration of FucNAcONAP (**8**) (0.25 mM) elicits more substantial effects on glycoprotein biosynthesis and fitness. Thus, modest changes in the concentration of FucNAcONAP (**8**) used in an experiment may afford control over the extent of the functional effects. We focused our attention on FucNAcONAP for subsequent experiments due to favorable effects observed at lower concentrations than DATONAP.

### *O*-Naphthylmethyl Glycoside FucNAcONAP Does Not Disrupt *B. fragilis* Glycans

After determining that FucNAcONAP (**8**) is the most potent metabolic inhibitor in the suite of novel substrate decoys developed, we sought to evaluate its selectivity by assessing its inhibitory effects on commensal gut bacteria. Specifically, we focused on *B. fragilis*, a common obligate anaerobic, gram-negative gut bacterium that constitutes a significant proportion of the gut microbiota. *B. fragilis* has been shown to possess remarkable immunomodulatory properties, primarily mediated by its capsular polysaccharide A (PSA), which plays a crucial role in alleviating inflammation and protecting the host from various diseases.^37^ Kasper and colleagues previously utilized MOE to incorporate peracetylated *N*-azidoacetylgalactosamine (Ac_4_GalNAz) into *B. fragilis* PSA.^38^ In this study, we leveraged the ability of *B. fragilis* to incorporate Ac_4_GalNAz (Gal) into its PSA to assess the inhibitory effect of FucNAcONAP (**8**) on PSA biosynthesis in this key commensal species. Consistent with previous reports, treatment with 0.5 mM Ac_4_GalNAz resulted in the robust metabolic incorporation of azides into PSA and other glycans, as confirmed by Western blot analysis (Figure 3J). Treatment of *B. fragilis* with FucNAcONAP (**8**) at concentrations ranging from 0.01 to 0.25 mM did not produce a significant effect on glycan biosynthesis, as indicated by sustained azide labeling in treated samples that were comparable to the positive control. Coomassie staining revealed equivalent protein concentrations in all of the samples analyzed (Figure S1A). To further evaluate the species-specific effects of FucNAcONAP (**8**), we examined its impact on *B. fragilis* growth. *B. fragilis* growth was as robust for cells treated with 0.25 mM FucNAcONAP (**8**) as it was for untreated samples (Figure 3K). These findings suggest that FucNAcONAP (**8**) exhibits selectivity between these two bacteria, consistent with our hypothesis about the selectivity afforded by rare bacterial monosaccharide epitopes.

## Discussion

Bacterial glycans play an essential role in pathogen survival and virulence and are, therefore, compelling targets for modulating host–pathogen interactions. The enormous structural diversity of bacterial glycans and the presence of rare bacterial monosaccharides that are expressed in a species- and serotype-specific fashion suggest an untapped potential for targeted glycan perturbation. Recent advances in the development of small-molecule inhibitors of glycan biosynthesis have highlighted the potential of these compounds as chemical tools to interrogate structure–function relationships and to validate specific glycan biosynthesis pathways as potential drug targets. Building upon previous successes in mammalian systems by Esko,^39–41^ Kim,^42^ Neelamegham,^23^ and others, as well as our prior work employing peracetylated *O*- and *S*-benzyl glycoside analogs of rare bacterial monosaccharides to inhibit bacterial glycosylation,^21,22^ we sought to explore the effect of putative metabolic inhibitors bearing larger aglycones on bacterial glycan biosynthesis. Motivated by reports that *O*-naphthylmethyl glycosides exhibit improved efficacy over *O*-benzyl glycosides in mammalian cells,^23^ we synthesized a new series of *O*-naphthylmethyl and *O*-anthracenemethyl glycoside analogs based on rare bacterial monosaccharides. These compounds expand the chemical biology toolkit, offer critical insights for how to improve potency, and fine-tune the species selectivity of compounds that disrupt *H. pylori* glycoprotein biosynthesis.

Previously reported *O*-benzyl^21^ glycosides FucOBn (**4**) and BacOBn (**5**) led to substantial inhibition of *H. pylori* glycoprotein biosynthesis at millimolar concentrations. Here, we found that *O*-anthracenemethyl glycosides **9–11** were not effective metabolic inhibitors as they exhibited slight inhibition of only low-molecular-weight glycoprotein (<43 kDa) biosynthesis in *H. pylori* even at 1 mM concentrations. By contrast, *O*-naphthylmethyl glycosides **6–7** showed modest activity as inhibitors of *H. pylori* glycoprotein biosynthesis across a range of molecular weights at 1 mM concentrations. Most notably, FucNAcONAP (**8**) displayed enhanced activity, achieving significant inhibition of *H. pylori* glycoprotein biosynthesis at 0.25 mM, suggesting higher potency than that of reported compounds. FucNAcONAP (**8**) did not exhibit any appreciable effect on glycan biosynthesis in the commensal bacterium *B. fragilis*, underscoring its selectivity, likely due to differential monosaccharide usage and expression. Beyond glycan inhibition and surface glycan profile changes, **6–8** impaired several key phenotypic traits of *H. pylori*, including motility, growth, and biofilm formation, to varying extents. Taken together, these findings highlight DATONAP (**7**) as an inhibitor of *H. pylori* glycoprotein biosynthesis and fitness when used at high concentrations (1.0 mM), and FucNAcONAP (**8**) is a more potent and selective chemical probe for glycan perturbation. These findings establish a framework that could yield insight into bacterial glycan structure–function relationships.

The choice of a rare monosaccharide scaffold appears to influence the efficacy of bacterial glycan biosynthesis inhibitors. This observation is evidenced by the differential inhibitory activity observed among the *O*-naphthylmethyl glycosides, with FucNAcONAP (**8**) exhibiting the most pronounced effects on *H. pylori* glycoprotein biosynthesis. This trend aligns with our previous findings with *O*-benzyl^21^ and *S*-benzyl^22^ analogs and suggests FucNAcONAP may be a privileged scaffold in *H. pylori*. FucNAc (**3**) may occupy a central or structurally critical position within *H. pylori* glycan architectures, potentially functioning as a key branching point or modification site. Alternatively, *H. pylori* glycosyltransferases that utilize FucNAc-containing acceptors as substrates may have lax substrate selectivity and readily catalyze the transfer of additional monosaccharides onto FucNAc-based decoy substrates. In the absence of detailed information on *H. pylori* glycan structure and biosynthesis, the precise mechanism by which FucNAcONAP (**8**) exerts its inhibitory effects remains speculative. Structure–activity relationship studies using an expanded panel of structurally diverse metabolic glycan inhibitors, including chain-terminating and substrate decoy analogs, may serve as valuable probe compounds to define the constraints of the system and yield mechanistic insight. Likewise, molecular-level evidence of the buildup of higher-order glycans on FucNAcONAP (**8**) would provide key insights of glycan biosynthesis in *H. pylori*. Further studies will be necessary to reveal precisely how rare bacterial monosaccharide analogs impact glycan biosynthesis at the molecular level and their basis for selectivity.

Our findings indicate that increasing the aromatic ring system from benzyl to naphthyl enhances the efficacy of glycoside-based inhibitors. However, further extension to an anthracene moiety resulted in a marked decrease in inhibitory activity. While the experimental data presented here do not elucidate the mechanistic basis for this observation, several hypotheses may be considered. The ability of glycosyltransferases to utilize glycosides bearing large aglycones as acceptor substrates may have a steric limit. Substrates bearing benzyl or naphthylmethyl aglycones may be accommodated by glycosyltransferase active sites, but oversized aglycones (e.g., anthracenemethyl) at the anomeric position may not be tolerated due to active site constraints. Indeed, there is a limit to aglycone promiscuity of glycosyltransferases.^43^ An alternative explanation for the reduced potency of the anthracenemethyl analogs relative to the naphthylenemethyl analogs could be due to differences in the configuration of the anomeric position. If glycosyltransferases prefer α-linked glycosyl acceptors, for example, these enzymes may bind to α-linked naphthylmethyl analogs with higher affinity than they bind to the corresponding β-linked anthracenemethyl analogs. Another possibility involves the role of bacterial hexosaminidases, which can degrade metabolic glycan analogs. Structural studies of both human and bacterial hexosaminidases have revealed that enzymatic activity relies on a glutamic acid residue within a polar catalytic pocket.^44,45^ It is plausible that the increased hydrophobicity introduced by the naphthyl group—but not the bulkier anthracene—sufficiently perturbs interactions with this catalytic site, thereby reducing degradation and enhancing the stability of the decoy substrate. Alternatively, differences in the bacterial metabolism of polycyclic aromatic hydrocarbons (PAHs) may play a role. Although no direct evidence exists that *H. pylori* can degrade PAH, one speculation might be that this bacterium can selectively degrade benzene or anthracene derivatives while sparing naphthalene analogs. Other cellular factors may influence inhibitor efficacy, including the compound’s ability to traverse the bacterial envelope or differences in uptake efficiency. These possibilities warrant further investigation to better understand the physicochemical and biological parameters governing the activity of glycan-targeting inhibitors.

This study demonstrates that glycan-disrupting analogs derived from rare bacterial monosaccharides possess the potential to attenuate pathogen fitness by impairing glycan-dependent functions essential for colonization and immune evasion. Among the compounds developed, FucNAcONAP (**8**) exhibited potent and selective activity, supporting its candidacy as a chemical probe for in vivo studies. Specifically, FucNAcONAP (**8**) could be deployed in animal infection models to assess its ability to selectively perturb *H. pylori* glycoprotein biosynthesis. This class of metabolic inhibitor offers a valuable platform for probing glycan architecture and elucidating the functional roles of specific glycan motifs in bacterial physiology and pathogenesis.

## Conclusion

This study developed novel *O*-naphthylmethyl and *O*-anthracenemethyl glycosides based on rare bacterial monosaccharide scaffolds and scored the effects of large aglycones relative to previously reported metabolic inhibitors of bacterial glycan biosynthesis. *O*-Naphthylmethyl d-*N*-acetylfucosamine (FucNAcONAP (**8**)) inhibited *H. pylori* glycan biosynthesis, reduced biofilm formation, and impeded *H. pylori* growth at lower concentrations than its *O*-benzyl analog. This potent and selective metabolic inhibitor of *H. pylori* glycan biosynthesis impairs critical bacterial functions including growth, motility, and biofilm formation—hallmarks of pathogenic fitness. Importantly, FucNAcONAP (**8**) demonstrates a minimal impact on the commensal bacterium *B. fragilis*, highlighting its selectivity. The structure–activity relationship observed across glycoside variants emphasizes the role of both the monosaccharide scaffold and aglycone in determining efficacy. This enabling information offers key insight into design parameters that influence the efficacy of substrate decoys. Collectively, these findings establish FucNAcONAP (**8**) as a valuable tool for probing the glycan structure and elucidating the functional roles of specific glycan motifs in bacterial physiology and pathogenesis.

## Methods

### Materials and Chemical Synthesis

All organic reagents and anti-FLAG antibodies were obtained from MilliporeSigma. *H. pylori* strain G2758 was generously provided by Dr. Manuel Amieva (Stanford University). *B. fragilis* (ATCC 23745) was purchased from ATCC and cultured according to the manufacturer’s instructions. Bowdoin’s Institutional Review Board deemed the studies performed in this work exempt from review. The azide-modified sugars Ac_4_GlcNAc, Ac_4_GlcNAz, Ac_4_GalNAz, and Phos-FLAG were synthesized following established protocols.^46^ *O*-Naphthylmethyl and *O*-anthracenemethyl glycoside were synthesized via standard organic chemistry techniques and characterized by ^1^H/^13^C NMR and mass spectrometry. Compounds were purified using flash silica gel chromatography.

### Metabolic Labeling

*H. pylori* cultures were grown under microaerophilic conditions (14% CO_2_, 37 °C) in rich media (Brucella broth with 10% FBS) supplemented with either 0.5 mM Ac_4_GlcNAz alone or in combination with 0.25–1.0 mM BacONAP, DATONAP, or FucNAcONAP (Figure 1D, **6–8**), 0.5–1.0 mM BacOAnth, DATOAnth, or FucOAnth (Figure 1D, **9–11**), or 0.01–0.25 mM FucNAcONAP (**8**). Controls received 0.5 mM azide-free Ac_4_GlcNAc. Cultures were incubated for 3 days. *B. fragilis* was labeled anaerobically (Oxoid EZ Anaerobe Gaspak, 37 °C) with 0.5 mM Ac_4_GalNAz, either alone or in combination with 0.1–0.25 mM analogs or with 0.5 mM Ac_4_GlcNAc as a control, for 2 days.

### Western Blot Analysis

Labeled cells were rinsed and incubated in lysis buffer (20 mM Tris–HCl, pH 7.4; 1% Igepal; 150 mM NaCl; 1 mM EDTA) containing a protease inhibitor cocktail (MilliporeSigma) for 30 min at −20 °C. Lysates were clarified by centrifugation at 10,000*g* using a microcentrifuge. *B. fragilis* lysates underwent additional freeze–thaw and sonication for 20 min at room temperature. Total protein concentrations were normalized to approximately 2.5 mg/mL using the DC protein assay (Bio-Rad). Lysates were incubated overnight at room temperature with 250 μM Phos-FLAG, followed by separation on 12% Tris–glycine (Bio-Rad) SDS-PAGE gels. Proteins were transferred onto nitrocellulose membranes and detected using anti-FLAG-HRP with chemiluminescence.

### Lectin Binding Assay

*H. pylori* cultures were treated with 1.0 mM BacONAP or DATONAP or 0.1 mM FucNAcONAP (Figure 1D, **6–8**) or were left untreated for 3 days. Cells were then stained with Alexa Fluor 488-conjugated concanavalin A (ConA), with or without 400 mM mannose pretreatment (blocking control), and analyzed via flow cytometry (BD Accuri C6 Plus; 10,000 live cells/sample). Data analysis was performed by using FlowJo software.

### Growth Curve Analysis

The growth of *H. pylori* and *B. fragilis* was monitored over 10 days in the presence of 1.0 mM BacONAP or DATONAP or 0.1 mM–0.25 mM FucNAcONAP (Figure 1, **6–8**). Cultures were initiated at OD_600_ ∼ 0.1 and incubated under the respective atmospheric conditions. OD_600_ readings were taken by using a SPECTROStar Nano plate reader.

### Viability Assay

*H. pylori* cultures were adjusted to an OD_600_ of 0.4 and incubated for 4 days with or without analogs. Bacterial viability was assessed using the LIVE/DEAD BacLight kit (Invitrogen), staining with SYTO 9 and propidium iodide. Samples were analyzed by flow cytometry (BD Accuri C6 Plus), and viability percentages were calculated by using FlowJo.

### Motility Assay

*H. pylori* cultures (OD_600_ 0.3–0.4) were treated with analogs or left untreated and then concentrated and spotted (10 μL) onto soft agar plates (4% agar, 10% FBS). Plates were incubated under microaerophilic conditions for 10 days. Colony diameters were measured at intervals and imaged on day 10.

### Biofilm Assay

Biofilm formation was assessed following the O’Toole method.^36^ *H. pylori* (OD_600_ 0.3–0.4) was cultured in 96-well plates with or without 1.0 mM BacONAP or DATONAP or 0.1 mM–0.25 mM FucNAcONAP (**8**) for 5 days (37 °C, 14% CO_2_). Wells were stained with 0.15% crystal violet, imaged, and solubilized in 30% acetic acid for quantification via the absorbance measurement.

### Crude LPS Extraction

*H. pylori* were cultured for 3 days with 0.25 mM FucNAcONAP (**8**) or no metabolic inhibitor and then harvested by centrifugation at 3500*g* for 15 min. Pellets were resuspended in lysis buffer (20 mM Tris–HCl, pH 7.4; 1% Igepal; 150 mM NaCl; 1 mM EDTA) supplemented with a protease inhibitor cocktail (MilliporeSigma, St. Louis, MO) and incubated at room temperature for 10 min. Lysates were clarified by centrifugation at 10,000*g* for 10 min. Pellet and supernatant fractions were mixed 1:1 (v/v) with LPS lysis buffer (composition: 4 mL of 10% SDS, 800 μL of β-mercaptoethanol, 1.2 mg of bromophenol blue, 2 mL of glycerol, 10 mL of 1.5 M Tris–HCl, and 5 mL of H_2_O) and heated at 100 °C for 10 min. Following heat treatment, samples were incubated overnight at 55 °C with proteinase K (20 mg/mL) added at a 1:30 (v/v) ratio to digest contaminating proteins.

### Pro-Q Emerald 300 Polysaccharide Stain

Crude lipopolysaccharide (LPS) samples from both pellet and supernatant fractions were boiled at 95 °C for 10 min prior to electrophoresis. Samples were resolved on a 4–20% Tris–glycine SDS-PAGE gel with a 4% stacking layer. Each lane was loaded with 10 μL of the sample alongside molecular weight markers: 10 μL of EZ-Run Prestained Recombinant Protein Ladder, 10 μL of CandyCane glycoprotein molecular weight standard (Thermo Fisher Scientific; prepared at 1:6 v/v from a 5 mg/mL stock), and 10 μL of LPS standard (MedChemExpress, from *E. coli* O55:B5, 2 mg/mL). Gels were run in 1× SDS running buffer (3.47 mM SDS, 24.71 mM Tris base, 191.95 mM glycine) at 200 V for 50 min. Following electrophoresis, the gel was stained using the Pro-Q Emerald 300 Glycoprotein Stain Kit (Thermo Fisher Scientific) according to the manufacturer’s instructions: The gels were fixed twice for 45 min in fixative solution (50% ethanol, 5% acetic acid) and then washed twice with 3% acetic acid. Carbohydrate oxidation was performed for 30 min in a solution containing 0.04 M periodic acid in 3% acetic acid. Gels were then stained with Pro-Q Emerald 300 stain (500 μL of stock solution in 25 mL of staining buffer) for 100 min in complete darkness. Stained polysaccharides were visualized under a 300 nm UV transilluminator.

## Supplementary Material



## Notes

Full experimental details, compound characterization data, comparing FucNAcONAP with FucOBn activity, protein loading controls, histograms of samples from flow cytometry analyses, lipopolysaccharide analysis, and *H. pylori* viability (PDF)

## Abbreviations

CPS: capsular polysaccharide
LPS: lipopolysaccharide
d-Bac: di-*N*-acetyl d-bacillosamine
d-DATDG: d-2,4-diacetamido-2,4,6-trideoxy galactose
d-FucNAc: *N*-acetyl d-fucosamine
MOE: metabolic oligosaccharide engineering
*H. pylori*: *Helicobacter pylori*
*B. fragilis*: *Bacteroides fragilis*
Ac_4_GlcNAc: peracetylated *N*-acetylglucosamine
Ac_4_GlcNAz: peracetylated *N*-azidoacetylglucosamine
Ac_4_GalNAz: peracetylated *N*-azidoacetylgalactosamine
NO_2_^–^: nitrite
TBAN_3_: tetrabutyl ammonium azide
EAS: electrophilic aromatic substitution
NIS: *N*-iodosuccinimide
TfOH: triflic acid
AnthOH: 9-anthracenemethanol
MeCN: acetonitrile
Ac_2_O: acetic anhydride
PPh_3_: triphenylphosphine
THF: tetrahydrofuran
H_2_O: water
Phos-FLAG: phosphine–FLAG conjugate
ConA: concanavalin A
OD_600_: optical density at 600 nm
CO_2_: carbon dioxide
K_2_CO_3_: potassium carbonate
ATCC: American Type Culture Collection
PBS: phosphate buffered saline
SDS: sodium dodecyl sulfate
NMR: nuclear magnetic resonance
HRP: horse radish peroxidase
EDTA: ethylenediaminetetraacetic acid
FBS: fetal bovine serum
