# Supplementary material for: Assessing O‑Naphthylmethyl and O‑Anthracenemethyl Glycosides as Metabolic Inhibitors of Bacterial Glycan Biosynthesis
Source: ACS Infect Dis. 2025 Sep 22;12(2):544–54. doi: 10.1021/acsinfecdis.5c00559 (PMC12910604; doi:10.1021/acsinfecdis.5c00559)
Supplement: Supplementary file 1 [file id5c00559_si_001.pdf]

Supporting Information

Assessing O-naphthylmethyl and O-anthracenemethyl glycosides as metabolic inhibitors of bacterial glycan biosynthesis

Panhasith Ung,[1#] Ankita Paul,[2#] Soumyakanta Maji,[2#] Pilar Saavedra-Weis,[1] Karen D. Moulton,[1] Suvarn S. Kulkarni,[2*] and Danielle H. Dube[1*]

[1]Department of Chemistry & Biochemistry, Bowdoin College,
2 Polar Loop, Brunswick, ME 04011, USA.
[2]Department of Chemistry, Indian Institute of Technology Bombay,
Powai, Mumbai 400-076, India.
*Corresponding authors: ddube@bowdoin.edu, suvarn@chem.iitb.ac.in

[#]These authors contributed equally to this work.

Table of Contents

Chemistry ***s-3***

General: **s-3**

Compound 16 .... **s-3**

Compound 6 .... **s-5**

Compound 17 .... **s-6**

Compound 7 .... **s-7**

Compound 18 .... **s-8**

Compound 8 .... **s-9**

Compound 19 .... **s-10**

Coumpound 9 .... **s-11**

Compound 20 .... **s-12**

Compound 10 .... **s-13**

Compound 21 .... **s-14**

Compound 11 .... **s-15**

Biology ***s-16***

Bacterial Strains and Reagents s-16
Bacterial Culture Conditions s-16
Metabolic Labeling of *H. pylori* s-16
SDS-PAGE and Western Blot Analysis s-16
Lectin Binding Flow Cytometry s-17
Growth Curve Analysis of *H. pylori* s-17
Viability Assays s-18
Motility Assays s-18
Biofilm Formation Assays s-18
Metabolic Labeling of *B. fragilis* s-19
Growth Curve Analysis of *B. fragilis* s-19
Crude LPS isolation and Pro-Q Emerald polysaccharide staining s-19
Supplemental Figures *s-20*
Figure S1 .... s-20
Figure S2 .... s-21
Figure S3 .... s-22
Supplemental References *s-23*
NMRs and MSs *s-24*

Chemistry

General:

All reactions were conducted under the dry nitrogen atmosphere. Solvents (CH_2Cl_2 >99%, THF 99.5%, acetonitrile 99.8%, DMF 99.5%) were purchased in capped bottles and dried under sodium or CaH_2. All other solvents and reagents were used without further purification. Glassware was oven dried before use. Reactions under heating condition were performed using paraffin oil bath. TLC was performed on pre-coated Aluminium plates of Silica Gel 60 F254 (0.25 mm, E. Merck). Developed TLC plates were visualized under a short-wave UV lamp and by heating plates that were dipped in ammonium molybdate/cerium (IV) sulphate solution. Silica gel column chromatography was performed using Silica Gel (100-200 mesh, as well as 230-400 mesh) and employed a solvent polarity correlated with TLC mobility. We have used 3 Angstrom powdered molecular sieves in our study. The powdered MS were weighed in a dried pear-shaped flask and activated by periodic heating of flask by using flame over a period of 15 minutes. NMR experiments were conducted on 500 and 400 MHz instrument using $CDCl_3$ (D, 99.8%), CD_3OD (D, 99.8%) or D_2O (D, 99.9%) as solvents. Chemical shifts are relative to the deuterated solvent peaks and are in parts per million (ppm). Structural assignments were made with additional information from gCOSY and gHSQC experiments. gCOSY was used to confirm proton assignments and gHSQC experiment was done to confirm proton carbon correlation. In the ^{1}H NMR spectrum, data are reported as follows: chemical shift (δ ppm), multiplicity (s: singlet, d: doublet, dd: doublet of doublet, t: triplet, q: quartet, m: multiplet, bs: broad singlet and ABq: AB quartet), coupling constant (J in Hz), integration and respective assigned proton(s). Mass spectra were acquired in the ESI-TOF mode. Specific rotation experiments were measured at 589 nm (Na) and 25 °C. IR spectra were recorded on an FT-IR spectrometer.

Compound 16:

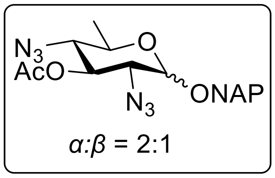

NBS (0.139 g, 0.784 mmol) was added to the stirred solution of compound **13**[1] (0.091 g, 0.261 mmol) in 4 mL THF/H_2O (5:1) at 0 °C. After 15 min, the reaction mixture was diluted with ethyl acetate and washed with aqueous $Na_2S_2O_3$ solution. The separated organic layer was dried over anhydrous Na_2SO_4, filtered, and concentrated. The crude compound was washed with hexane to remove the non-polar impurity and taken to next step. K_2CO_3 (0.180 g, 1.305 mmol), CCl_3CN (1.3 mL, 1.305 mmol) was added to the hemiacetal (0.067 g, 0.261 mmol) in dry CH_2Cl_2 and stirred at rt for 8 h. After completion of reaction, K_2CO_3 is filtered through celite, the filtrate is concentrated and crude imidate compound was taken for next step.

Crude imidate donor (0.261 mmol), 2-naphthylmethylalcohol (0.062 g, 0.391 mmol) and molecular sieves (3Å, 150 mg) were dissolved in dry CH_2Cl_2 (3 mL). Then, TfOH (0.04 mL, 0.444 mmol) was added dropwise to the solution at 0 ºC. After 2 h, with completion of reaction, mixture was quenched by Et_3N and molecular sieves were filtered out through Celite. Then crude was concentrated and purified by flash column chromatography (15% ethyl acetate: petroleum ether) to afford compound **16** as α:β (2:1) (0.051 g, 50% over three steps) as sticky colorless gum.

$[\alpha]_D^{25}$ +5.20 (c = 0.2, $CHCl_3$).

IR (cm^{-1}, $CHCl_3$) ν 3500, 2109, 1723, 1463, 1036, 758.

^{1}H NMR (500 MHz, $CDCl_3$) δ 7.90-7.84 (m, 7H, ArH), 7.54-7.42 (m, 5H, ArH), 5.42 (t, J = 10 Hz, 1H, H-3α), 5.10 (t, J = 15 Hz, 1H, H-3β), 4.93, 4.79 (ABq(β), J = 12 Hz, 2H, CH_2ONAP), 5.03 (d, J = 3.5 Hz, 1H, H-1α), 4.97, 4.86 (ABq (α), J = 12Hz, 2H, CH_2ONap), 4.48 (d, J = 8 Hz, 1H, H-1β), 3.83-3.80 (m, 1H, H-5α), 3.52 (dd, J = 10, 1.0 Hz, 1H, H-2β), 3.37-3.54 (m, 1H, H-5β), 3.52-3.17 (m, 3H, H-2α, H-4 α, H-4β), 2.21 (s, 3H, OAcα), 2.20 (s, 3H, OAcβ), 1.44 (d, J = 5 Hz, 3H, H-6β), 1.35 (d, J = 5 Hz, 3H, H-6α).

^{13}C{1H} NMR (125 MHz, $CDCl_3$) δ 169.9, 133.7, 133.6, 133.2, 128.5, 128.46, 128.0, 127.6, 127.3, 127.1, 126.3, 126.29, 126.2, 125.9, 125.8, 100.2, 96.6, 72.8, 71.4, 70.7, 69.8, 66.42, 66.38, 65.8, 64.2, 61.3, 20.8, 18.3, 18.2.

HRMS (ESI-TOF) (m/z): $[M+Na]^+$ calcd. for $C_{19}H_{20}N_6NaO_4$ 419.1432; found, 419.1433.

Compound 6:

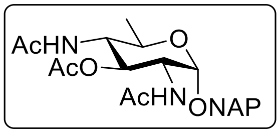

To compound **16** (0.040 g, 0.101 mmol) in THF (1.5 mL), activated Zn dust (60 mg) was added followed by dropwise addition of AcOH (0.3 mL) at rt. Mixture was allowed to stir at same temperature for 8 h. After complete conversion of azide to amine, zinc was filtered through celite pad, concentrated and dried under high *vacuum* for 30 min.

Crude amine compound was dissolved in THF (2 mL). To the clear solution, Ac_2O (0.04 mL, 0.404 mmol), Et_3N (0.04 mL) and DMAP (5 mg, 0.040 mmol) were added sequentially at 0 °C, mixture was allowed to stir at room temperature for 2 h. After completion of reaction, solvents were removed in *vacuo* and the crude product was purified by column chromatography over silica gel (40% ethyl acetate: petroleum ether) to furnish desired substrate **6** as colourless viscous liquid (0.027 g, 63%).

$[\alpha]_D^{25}$ +40.12 (c = 0.2, $CHCl_3$).

IR (cm^{-1}, $CHCl_3$) ν 3031, 2524, 1722, 1364, 1227, 1045, 753.

^{1}H NMR (400 MHz, $CDCl_3$) δ 7.88-7.78 (m, 4H, ArH), 7.54-7.28 (m, 3H, ArH), 5.69 (d, J = 9.6 Hz, 1H, NH), 5.45 (d, J = 9.2 Hz, 1H, NH), 5.06 (t, J = 10.4 Hz, 1H, H-3), 4.95 (d, J = 3.6 Hz, 1H, H-1), 4.86, 4.67 (ABq, J = 12 Hz, 2H, CH_2ONAP), 4.42-4.37 (m, 1H, H-2), 4.03-4.98 (m, 1H, H-4), 3.82-3.77 (m, 1H, H-5), 2.04 (s, 3H, OAc), 1.95(s, 3H, NHAc), 1.90 (s, 3H, NHAc), 1.25 (d, J = 6.0 Hz, 3H, H-6).

^{13}C{1H} NMR (100 MHz, $CDCl_3$) δ 172.3, 169.9, 169.7, 134.2, 133.2, 133.1, 128.5, 127.8, 127.7, 127.2, 126.5, 126.4, 125.8, 96.7, 71.6, 70.0, 67.6, 54.9, 51.7, 23.3, 23.2, 20.8, 17.8.

HRMS (ESI-TOF) (m/z): $[M+Na]^+$ calcd. for $C_{23}H_{28}N_2NaO_6$ 451.1805; found, 451.1800.

Compound 17:

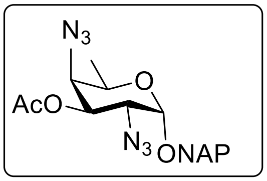

NBS (0.069 g, 0.392 mmol) was added to the stirred solution of compound **14**[1] (0.045 g, 0.130 mmol) in 3 mL THF/H_2O (5:1) at 0 °C. After 15 min, the reaction mixture was diluted with ethyl acetate and washed with aqueous $Na_2S_2O_3$ solution. The separated organic layer was dried over anhydrous Na_2SO_4, filtered, and concentrated. The crude compound was washed with hexane to remove the non-polar impurity and taken to next step. K_2CO_3 (0.090 g, 0.652 mmol), CCl_3CN (0.6 mL, 0.652 mmol) was added to the hemiacetal (0.033 g, 0.130 mmol) in dry CH_2Cl_2 and stirred at rt for 8 h. After completion of reaction, K_2CO_3 is filtered through celite, the filtrate is concentrated and crude imidate compound was taken for next step.

Crude imidate donor (0.130 mmol), 2-naphthylmethylalcohol (0.031 g, 0.195 mmol) and molecular sieves (3Å, 100 mg) were dissolved in dry CH_2Cl_2 (3 mL). Then, TfOH (0.02 mL, 0.222 mmol) was added dropwise to the solution at 0 ºC. After 2 h, with completion of reaction, mixture was quenched by Et_3N and molecular sieves were filtered out through Celite. Then crude was concentrated and purified by flash column chromatography (20% ethyl acetate: petroleum ether) to afford compound **17** (0.028 g, 55% over three steps) as sticky colorless gum.

$[\alpha]_D^{25}$ +4.64 (c = 0.15, $CHCl_3$).

IR (cm^{-1}, $CHCl_3$) *ν* 2924, 2115, 1721, 1633, 1205, 1034, 745, 694.

^{1}H NMR (500 MHz, $CDCl_3$) *δ* 7.89-7.84 (m, 4H, ArH), 7.53-7.51 (m, 3H, ArH), 5.49 (dd, *J* = 11, 3.5 Hz, 1H, H-3), 5.05 (d, *J* = 3.5 Hz, 1H, H-1), 4.89, 4.78 (ABq, *J* = 12 Hz, 2H, CH_2ONAP), 4.18-4.15 (m, 1H, H-5), 3.96 (d, *J* = 2.5 Hz, 1H, H-4), 3.75 (dd, *J* = 11, 3.4 Hz, 1H, H-2), 2.22 (s, 3H, OAc), 1.27 (d, *J* = 6.5 Hz, 3H, H-6).

^{13}C{1H} NMR (125 MHz, $CDCl_3$) *δ* 170.2, 134.0,133.3, 128.5, 128.0, 127.8, 127.2, 126.4, 126.3, 125.9, 97.0, 71.2, 70.1, 74.9, 74.1, 57.6, 20.7, 17.2.

HRMS (ESI-TOF) (m/z): $[M+Na]^+$ calcd. for $C_{19}H_{20}N_6NaO_4$ 419.1432; found, 419.1432.

Compound 7:

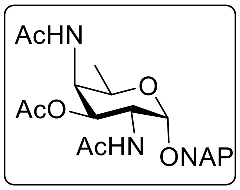

To compound **17** (0.045 g, 0.114 mmol) in THF (2 mL), activated Zn dust (40 mg) was added followed by dropwise addition of AcOH (0.2 mL) at rt. Mixture was allowed to stir at same temperature for 8 h. After complete conversion of azide to amine, zinc was filtered through celite pad, concentrated and dried under high *vacuum* for 30 min.

Crude amine compound was dissolved in THF (1 mL). To the clear solution, Ac_2O (0.05 mL, 0.456 mmol), Et_3N (0.05 mL) and DMAP (4 mg, 0.045 mmol) were added sequentially at 0 °C, mixture was allowed to stir at room temperature for 2 h. After completion of reaction, solvents were removed in *vacuo* and the crude product was purified by column chromatography over silica gel (40% ethyl acetate: petroleum ether) to furnish desired substrate **7** as colourless viscous liquid (0.035 g, 73%).

$[\alpha]_D^{25}$ +42.60 (c = 0.4, $CHCl_3$).

IR (cm^{-1}, $CHCl_3$) ν 3378, 2946, 2835, 1723, 1652, 1252, 1110, 1024, 920.

^{1}H NMR (400 MHz, $CDCl_3$) δ 7.85-7.71 (m, ArH, 4H), 7.5 -7.37 (m, ArH, 3H), 6.36 (d, J = 9.4 Hz, 1H, NH), 5.86 (d, J = 9 Hz, 1H, NH), 5.16 (dd, J = 12.0, 4.0 Hz, 1H, H-3), 4.90 (d, J = 4.0 Hz, 1H, H-1), 4.79, 4.57 (ABq, J = 12.5 Hz, 2H, CH_2ONAP), 4.47-4.37 (m, 2H, H-2, H-4), 4.25-4.19 (m, 1H, H-5), 2.07 (s, 3H, OAc), 1.99 (s, 3H, NHAc), 1.90 (s, 3H, NHAc), 1.10 (d, 3H, J = 6.5 Hz, H-6).

^{13}C{1H} NMR (100 MHz, $CDCl_3$) δ 171.4, 171.2, 170.5, 134.3, 133.25, 133.17, 128.6, 127.94, 127.87, 127.1, 126.6, 126.4, 125.7, 97.0, 70.2, 69.5, 65.2, 50.8, 48.2, 23.3, 21.1, 16.5.

HRMS (ESI-TOF) (m/z): $[M+Na]^+$ calcd. for $C_{23}H_{28}N_2NaO_6$ 451.1805; found, 451.1804.

Compound 18:

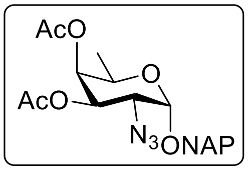

NBS (0.234 g, 1.314 mmol) was added to the stirred solution of compound **15**[1] (0.160 g, 0.438 mmol) in 5 mL THF/H_2O (5:1) at 0 °C. After 15 min, the reaction mixture was diluted with ethyl acetate and washed with aqueous $Na_2S_2O_3$ solution. The separated organic layer was dried over anhydrous Na_2SO_4, filtered, and concentrated. The crude compound was washed with hexane to remove the non-polar impurity and taken to next step. K_2CO_3 (0.302 g, 2.19 mmol), CCl_3CN (0.2 mL, 2.19 mmol) was added to the hemiacetal (0.119 g, 0.438 mmol) in dry CH_2Cl_2 and stirred at rt for 8 h. After completion of reaction, K_2CO_3 is filtered through celite, the filtrate is concentrated and crude imidate compound was taken for next step.

Crude imidate donor (0.438 mmol), 2-naphthylmethylalcohol (0.134 g, 0.657 mmol) and molecular sieves (3Å, 200 mg) were dissolved in dry CH_2Cl_2 (3 mL). Then, TfOH (6 µl, 0.065 mmol) was added dropwise to the solution at 0 ºC. After 2 h, with completion of reaction, mixture was quenched by Et_3N and molecular sieves were filtered out through Celite. Then crude was concentrated and purified by flash column chromatography (20% ethyl acetate: petroleum ether) to afford compound **18** (0.097 g, 54% over three steps) as sticky colorless gum.

$[\alpha]_D^{25}$ +3.60 (c = 0.2, $CHCl_3$).

IR (cm^{-1}, $CHCl_3$) *ν* 2923, 2112, 1723, 1582, 1271, 1033, 745, 694.

^{1}H NMR (400 MHz, $CDCl_3$) *δ* 7.89-7.86 (m, 4H, ArH), 7.53-7.50 (m, 3H, ArH), 5.46 (dd, *J* = 11.2, 3.2 Hz, 1H, H-3), 5.33 (d, *J* = 2.4 Hz, 1H, H-4), 5.09 (d, *J* = 3.6 Hz, 1H, H-1), 4.93, 4.80 (ABq, *J* = 12 Hz, 2H, CH_2NAP), 4.23-4.21 (m, 1H, H-5), 3.69 (dd, *J* = 11.2, 3.6 Hz, 1H, H-2), 2.18 (s, 3H, OAc), 2.08 (s, 3H, OAc), 1.15 (d, *J* = 6.4 Hz, 3H, H-6).

^{13}C{1H} NMR (100 MHz, $CDCl_3$) *δ* 170.4, 169.9, 133.9, 133.2, 133.1, 128.4, 127.9, 127.7, 127.1, 126.3, 126.2, 125.8, 97.0, 70.3, 70.0, 68.7, 65.0, 57.5, 20.7, 20.6, 15.9.

HRMS (ESI-TOF) (m/z): $[M+Na]^+$ calcd. for $C_{21}H_{23}NaN_3O_6$ 436.1485; found, 436.1481.

Compound 8:

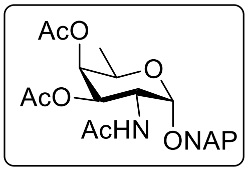

To compound **18** (0.050 g, 0.120 mmol) in THF (2 mL), activated Zn dust (75 mg) was added followed by dropwise addition of AcOH (0.4 mL) at rt. Mixture was allowed to stir at same temperature for 8 h. After complete conversion of azide to amine, zinc was filtered through celite pad, concentrated and dried under high *vacuum* for 30 min.

Crude amine compound was dissolved in THF (2 mL). To the clear solution, Ac_2O (0.05 mL, 0.480 mmol), Et_3N (0.05 mL) and DMAP (2 mg, 0.012 mmol) were added sequentially at 0 °C, mixture was allowed to stir at room temperature for 2 h. After completion of reaction, solvents were removed in *vacuo* and the crude product was purified by column chromatography over silica gel (30% ethyl acetate: petroleum ether) to furnish desired substrate **8** as yellow viscous liquid (0.042 g, 81%).

$[\alpha]_D^{25}$ +20.01 (c = 0.15, $CHCl_3$).

IR (cm^{-1}, $CHCl_3$) *v* 2944, 2553, 2320, 1723, 1246, 1025, 920.

^{1}H NMR (400 MHz, $CDCl_3$) δ 7.88-7.78 (m, 4H, ArH), 7.54-7.43 (m, 3H, ArH), 5.65 (m, J = 9.6 Hz, 1H, NH), 5.26-5.22 (m, 2H, H-3, H-4), 5.00 (d, J = 3.6 Hz, 1H, H-1), 4.87, 4.68 (ABq, J = 12 Hz, 2H, CH_2NAP), 4.63-4.19 (m, 1H, H-2), 4.19-4.17 (m, 1H, H-5), 2.20 (s, 3H, OAc), 2.00 (s, 3H, OAc), 1.90 (s, 3H, NHAc), 1.15 (d, J = 6.4 Hz, 3H, H-6).

^{13}C{1H} NMR (100 MHz, $CDCl_3$) δ 171.1, 170.8, 169.9, 134.3, 133.2, 133.1, 128.5, 127.8, 127.7, 127.2, 126.5, 126.3, 125.8, 97.2, 70.5, 70.2, 68.9, 65.2, 47.7, 23.3, 20.8, 20.79, 16.1.

HRMS (ESI-TOF) (m/z): $[M+H]^+$ calcd. for $C_{23}H_{28}NO_7$ 430.1866; found, 430.1863.

Compound 19:

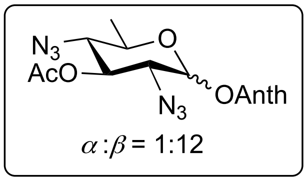

NBS (0.15 g, 0.64 mmol) was added to the stirred solution of compound **13**[1] (0.15 g, 0.43 mmol) in 4 mL THF/H_2O (5:1) at 0 °C. After 15 min, the reaction mixture was diluted with ethyl acetate and washed with aqueous $Na_2S_2O_3$ solution. The separated organic layer was dried over anhydrous Na_2SO_4, filtered, and concentrated. The crude compound was washed with hexane to remove the non-polar impurity and taken to next step. K_2CO_3 (0.59 g, 4.30 mmol), CCl_3CN (0.06 mL, 0.64 mmol) was added to the hemiacetal (0.067 g, 0.261 mmol) in dry CH_2Cl_2 and stirred at rt for 8 h. After completion of reaction, K_2CO_3 is filtered through celite, the filtrate is concentrated, kept in vacuum for 15 min and crude imidate compound was taken for next step.

Crude imidate donor, 9-anthracenemethylalcohol (0.13 g, 0.64 mmol) and molecular sieves (3Å, 150 mg) were dissolved in dry CH_2Cl_2 (3 mL). Then, TfOH (7 µL, 0.08 mmol) was added dropwise to the solution at 0 ºC. After 1 h, with completion of reaction, mixture was quenched by Et_3N and molecular sieves were filtered out through Celite. Then crude was concentrated and purified by flash column chromatography (10% Ethyl acetate-petroleum ether) to afford compound **19** as *α:β* (1:12) (0.152 g, 74% over three steps) as sticky colorless gum.

$[\alpha]_D^{25}$ +16.78 (c = 0.22, $CHCl_3$).

IR (cm^{-1}, $CHCl_3$) *ν* 3217, 2876, 2109, 1733, 1473, 1234, 1025, 767.

^{1}H NMR (500 MHz, $CDCl_3$) (for *β* isomer) *δ* 8.53 (s, 1H, ArH), 8.45-8.43 (m, 2H, ArH), 8.06-8.04 (m, 2H ArH), 7.61-7.58 (m, 2H, ArH), 7.53-7.50 (m, 2H, ArH), 5.85, 5.79 (ABq, *J* = 12.0 Hz, 2H, CH_2OAnth), 4.71 (t, *J* = 9.8 Hz, 1H, H-3), 4.25 (d, *J* = 8.1 Hz, 1H, H-1), 3.41 (dd, *J* = 9.8, 8.1 Hz, 1H, H-2), 3.19-3.10 (m, 2H, H-4, H-5), 2.13 (s, 3H, OAc), 1.48 (d, *J* = 5.8 Hz, 3H, H-6).

^{13}C{1H} NMR (125 MHz, $CDCl_3$) *δ* 169.7, 131.6, 131.4, 129.4, 129.2, 129.1, 126.6, 126.1, 125.2, 123.9, 99.0, 72.7, 70.7, 65.7, 64.0, 62.5, 20.8, 18.3.

HRMS (ESI-TOF) (m/z): $[M+NH_4]^+$ calcd. for $C_{23}H_{26}N_7O_4$ 464.2041; found, 464.1936.

Compound 9:

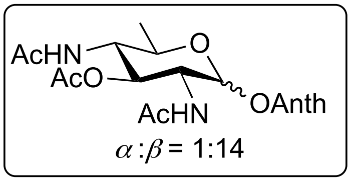

To a solution of compound **19** (0.05 g, 0.11 mmol) in 9:1 ratio of THF(1.8 mL) and H_2O (0.2 mL), Py (0.18 mL, 2.24 mmol) was added , Mixture was allowed to stir at same temperature for 12 h at 0 °C. After complete conversion of azide to amine, the reaction mixture was concentrated and dried under high vacuum for 30 min.

Crude amine compound was dissolved in Py (2 mL). To the clear solution, Ac_2O (0.05 mL, 0.55 mmol)was added, mixture was allowed to stir at room temperature for 4 h. After completion of reaction, solvents were removed in *vacuo* and the crude product was purified by column chromatography over silica gel (5% methanol:ethyl acetate) to furnish desired substrate **9** as brownish viscous liquid (39 mg, 74%).

$[\alpha]_D^{25}$ +31.12 (c = 0.16, $CHCl_3$).

IR (cm^{-1}, $CHCl_3$) ν 3056, 3016, 3032, 1745, 1363, 1227, 1049, 768.

^1H NMR (400 MHz, $CDCl_3$:MeOD = 2:1) (for β isomer) δ 8.34 (s, 1H, ArH), 8.23 (d, J = 8.7 Hz, 2H, ArH), 7.87 (d, J = 8.2 Hz, 2H, ArH), 7.39 (t, J = 8.2 Hz, 2H, ArH), 7.32 (d, J = 7.2 Hz, 2H, ArH), 5.67, 5.53 (ABq , J = 12.3 Hz, 2H, CH_2OAnth), 4.74 (t, J = 10.3 Hz, 1H, H-3), 4.28 (d, J = 8.4 Hz, 1H, H-1), 3.60-3.54 (m, 2H, H-2, H-4), 3.25-3.33 (m, 1H, H-5), 1.77 (s, 3H, OAc), 1.70 (s, 3H, NHAc), 1.39 (s, 3H, NHAc), 1.22 (d, J = 6.1 Hz, 3H, H-6).

$^{13}C\{1H\}$ NMR (100 MHz, $CDCl_3$:MeOD = 2:1) (for β isomer) δ 171.51, 171.45, 171.4, 131.4, 131.2, 128.8, 128.6, 126.9, 126.2, 125.0, 124.0, 97.2, 72.1, 70.6, 61.5, 55.3, 54.6, 22.3, 22.2, 20.2, 17.6.

HRMS (ESI-TOF) (m/z): $[M+Na]^+$ calcd. for $C_{27}H_{30}N_2O_6Na$ 501.1996; found, 501.2003.

Compound 20:

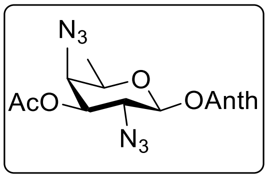

NBS (0.15 g, 0.64 mmol) was added to the stirred solution of compound **14**[1] (0.15 g, 0.43 mmol) in 4 mL THF/H_2O (5:1) at 0 °C. After 15 min, the reaction mixture was diluted with ethyl acetate and washed with aqueous $Na_2S_2O_3$ solution. The separated organic layer was dried over anhydrous Na_2SO_4, filtered, and concentrated. The crude compound was washed with hexane to remove the non-polar impurity and taken to next step. K_2CO_3 (0.59 g, 4.30 mmol), CCl_3CN (0.06 mL, 0.64 mmol) was added to the hemiacetal (0.067 g, 0.261 mmol) in dry CH_2Cl_2 and stirred at rt for 8 h. After completion of reaction, K_2CO_3 is filtered through celite, the filtrate is concentrated, kept in vacuum for 15 min, and crude imidate compound was taken for next step.

Crude imidate donor, 9-anthracenemethylalcohol (0.13 g, 0.64 mmol) and molecular sieves (3Å, 150 mg) were dissolved in dry CH_2Cl_2 (3 mL). Then, TfOH (7 µL, 0.08 mmol) was added dropwise to the solution at 0 ºC. After 1 h, with completion of reaction, mixture was quenched by Et_3N and molecular sieves were filtered out through Celite. Then crude was concentrated and purified by flash column chromatography (10% EtOAc-petroleum ether) to afford compound **20** (0.146 g, 76% over three steps) as reddish gum.

$[\alpha]_D^{25}$ +9.56 (c = 0.28, $CHCl_3$).

IR (cm^{-1}, $CHCl_3$) *ν* 3228, 2937, 2118, 1734, 1656, 1367, 1205, 1036, 747, 695.

^{1}H NMR (400 MHz, $CDCl_3$) *δ* 8.49 (s, 1H, ArH), 8.44 (d, *J* = 8.8 Hz, 2H, ArH), 8.02 (d, *J* = 8.4 Hz, 2H, ArH), 7.58-7.53 (m, 2H ArH), 7.50-7.46 (m, 2H, ArH), 5.87, 5.75 (ABq, *J* = 12.4 Hz, 2H, CH_2OAnth), 4.54 (dd, *J* = 10.7, 3.7 Hz, 1H, H-3), 4.10 (d, *J* = 8.0 Hz, 1H, H-1), 3.74-3.68 (m, 2H, H-2, H-4), 3.41 (qd, *J* = 6.3, 1.2 Hz, 1H, H-5), 2.10 (s, 3H, OAc), 1.43 (d, *J* = 6.3 Hz, 3H, H-6).

^{13}C{1H} NMR (100 MHz, $CDCl_3$) *δ* 170.1, 131.7, 131.4, 129.3, 129.2, 126.5, 126.3, 125.2, 124.2, 99.0, 73.4, 69.3, 63.0, 62.1, 60.7, 20.6, 17.4.

HRMS (ESI-TOF) (m/z): $[M+NH_4]^+$ calcd. for $C_{23}H_{26}N_7O_4$ 464.2041; found, 464.1893.

Compound 10:

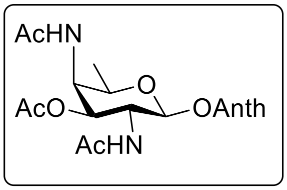

To compound **20** (0.05 g, 0.11 mmol) in THF (2 mL), activated Zn dust (40 mg) was added followed by dropwise addition of AcOH (0.2 mL) at rt. Mixture was allowed to stir at same temperature for 2 h. After complete conversion of azide to amine, zinc was filtered through celite pad, concentrated and dried under high *vacuum* for 30 min.

Crude amine compound was dissolved in THF (1 mL). To the clear solution, Ac_2O (0.05 mL, 0.66 mmol), Et_3N (0.05 mL) and DMAP (4 mg, 0.045 mmol) were added sequentially at 0 °C, mixture was allowed to stir at room temperature for 2 h. After completion of reaction, solvents were removed in *vacuo* and the crude product was purified by column chromatography over silica gel (60% ethyl acetate: petroleum ether) to furnish desired substrate **10** as red colour viscous liquid (43 mg, 81%).

$[\alpha]_D^{25}$ +16.54 (c = 0.23, $CHCl_3$).

IR (cm^{-1}, $CHCl_3$) ν 3378, 3154, 2956, 1734, 1652, 1487, 1252, 1110, 1024, 920, 767.

^{1}H NMR (500 MHz, $CDCl_3$:MeOD = 2:1) δ 8.29 (s, 1H, ArH), 8.17 (d, J = 8.8 Hz, 2H, ArH), 8.02 (d, J = 8.3 Hz, 2H, ArH), 7.35 (t, J = 6.9 Hz, 2H, ArH), 7.30 (t, J = 8.0 Hz, 2H, ArH), 5.64, 5.46 (ABq, J = 12.5 Hz, 2H, CH_2OAnth), 4.40 (dd, J = 11.2, 4.2 Hz, 1H, H-3), 4.09-4.06 (m, 1H, H-4), 3.94 (d, J = 8.2 Hz, 1H, H-1), 3.77 (t, J = 10.5 Hz, 1H, H-2), 3.36 (q, J = 6.2 Hz, 1H, H-5), 1.87 (s, 3H, NHAc), 1.69 (s, 3H, NHAc), 1.38 (s, 3H, OAc), 1.11 (d, J = 6.2 Hz, 3H, H-6).

^{13}C{1H} NMR (125 MHz, $CDCl_3$:MeOD = 2:1) δ 172.6, 171.8, 170.9, 131.3, 131.2, 128.8, 126.6, 126.2, 124.9, 123.8, 98.6, 71.4, 69.1, 61.5, 50.2, 49.7, 22.1, 22.0, 20.2, 16.3.

HRMS (ESI-TOF) (m/z): $[M+Na]^+$ calcd. for $C_{27}H_{30}N_2O_6Na$ 501.1996; found, 501.2002.

Compound 21:

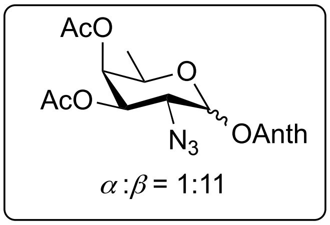

NBS (0.1 g, 0.61 mmol) was added to the stirred solution of compound **15**[1] (0.15 g, 0.41 mmol) in 4 mL THF/H_2O (5:1) at 0 °C. After 15 min, the reaction mixture was diluted with ethyl acetate and washed with aqueous $Na_2S_2O_3$ solution. The separated organic layer was dried over anhydrous Na_2SO_4, filtered, and concentrated. The crude compound was washed with hexane to remove the non-polar impurity and taken to next step. K_2CO_3 (0.56 g, 4.10 mmol), CCl_3CN (0.06 mL, 0.61 mmol) was added to the above generated hemiacetal in dry CH_2Cl_2 and stirred at rt for 8 h. After completion of reaction, K_2CO_3 is filtered through celite, the filtrate is concentrated, kept in vacuum for 15 min and crude imidate compound was taken for next step.

Crude imidate donor, 9-anthracenemethylalcohol (0.13 g, 0.61 mmol) and molecular sieves (3Å, 150 mg) were dissolved in dry CH_2Cl_2 (3 mL). Then, TfOH (7 µL, 0.08 mmol) was added dropwise to the solution at 0 ºC. After 1 h, with completion of reaction, mixture was quenched by Et_3N and molecular sieves were filtered out through Celite. Then crude was concentrated and purified by flash column chromatography (10% ethyl acetate-petroleum ether) to afford compound **21** as *α*:*β* (1:11) (0.135 g, 71% over three steps) as colorless gum.

$[\alpha]_D^{25}$ +8.85 (c = 0.62, $CHCl_3$).

IR (cm^{-1}, $CHCl_3$) *ν* 3134, 2923, 2119, 1749, 1630, 1576, 1271, 1033, 748, 686.

^{1}H NMR (400 MHz, $CDCl_3$) (for *β* isomer) 8.52-8.45 (m, 3H, ArH), 8.03 (d, *J* = 8.4 Hz, 2H, ArH), 7.58-7.47 (m, 4H, ArH), 5.92, 5.80 (ABq, *J* = 12.2 Hz, 2H, CH_2OAnth), 5.08 (d, *J* = 2.4 Hz, 1H, H-4), 4.50 (dd, *J* = 10.7, 2.4 Hz, 1H, H-3), 4.19 (d, *J* = 8.0 Hz, 1H, H-1), 3.67 (dd, *J* = 10.7, 8.0 Hz, 1H, H-2), 3.52 (q, *J* = 5.9 Hz, 1H, H-5), 2.17 (s, 3H, OAc), 1.96 (s, 3H, OAc), 1.31 (d, *J* = 5.9 Hz, 3H, H-6).

^{13}C{1H} NMR (100 MHz, $CDCl_3$) (for *β* isomer) *δ* 170.7, 169.9, 131.8, 131.5, 129.4, 129.2, 126.6, 125.3, 124.2, 99.2, 71.3, 69.8, 69.3, 62.4, 60.9, 20.8, 20.7, 16.3.

HRMS (ESI-TOF) (m/z): $[M+H]^+$ calcd. for $C_{25}H_{26}N_3O_6$ 486.1636; found, 486.1637.

Compound 11:

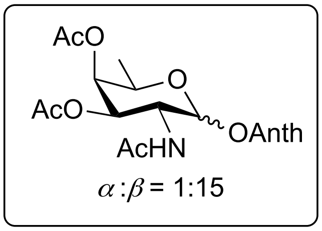

To compound **21** (0.05 g, 0.10 mmol) in THF (2 mL), activated Zn dust (40 mg) was added followed by dropwise addition of AcOH (0.2 mL) at rt. Mixture was allowed to stir at same temperature for 2 h. After complete conversion of azide to amine, zinc was filtered through celite pad, concentrated and dried under high *vacuum* for 30 min.

Crude amine compound was dissolved in THF (1 mL). To the clear solution, Ac_2O (0.05 mL, 0.53 mmol), Et_3N (0.05 mL) and DMAP (4 mg, 0.045 mmol) were added sequentially at 0 °C, mixture was allowed to stir at room temperature for 2 h. After completion of reaction, solvents were removed in *vacuo* and the crude product was purified by column chromatography over silica gel (5% methanol:ethyl acetate) to furnish desired substrate **11** as α:β (1:15) as red colour viscous liquid (43 mg, 75%).

$[\alpha]_D^{25}$ +14.23 (c = 0.18, $CHCl_3$).

IR (cm^{-1}, $CHCl_3$) ν 3567, 3112, 2948, 2553, 2220, 1723, 1246, 1038, 920, 735.

^{1}H NMR (400 MHz, $CDCl_3$) (for β isomer) δ 8.52 (s, 1H, ArH), 8.42 (d, J = 8.8 Hz, 2H, ArH), 8.04 (d, J = 8.3 Hz, 2H, ArH), 7.58-7.48 (m, 4H, ArH), 5.92, 5.68 (ABq, J = 12.8 Hz, 2H, CH_2OAnth), 5.06 (d, J = 3.1 Hz, 1H, H-4), 4.74-4.72 (m, 2H, H-3), 4.20-4.14 (m, 2H, H-1, H-2), 3.51 (q, J = 6.3 Hz, 1H, H-5), 2.20 (s, 3H, NHAc), 1.90 (s, 3H, NHAc), 1.55 (s, 3H, OAc), 1.34 (d, J = 6.3 Hz, 3H, H-6).

^{13}C{1H} NMR (100 MHz, $CDCl_3$) (for β isomer) δ 170.9, 170.8, 170.3, 131.7, 131.5, 129.3, 129.2, 127.0, 126.6, 125.3, 124.2, 97.8, 70.8, 70.0, 69.4, 61.6, 50.6, 23.3, 20.9, 20.7, 16.5.

HRMS (ESI-TOF) (m/z): $[M+H]^+$ calcd. for $C_{27}H_{30}NO_7$, 480.2017; found, 480.2008.

Biology

Bacterial Strains and Reagents

Helicobacter pylori strain G27,[2] generously provided by Manuel Amieva (Stanford University), and *Bacteroides fragilis* strain ATCC 23745 were utilized in this study. Bowdoin's Institutional Review Board deemed the studies performed in this work exempt from review. All biological reagents were procured from commercial suppliers including MilliporeSigma, Fisher Scientific, Thermo Fisher Scientific, Bio-Rad, and Click Chemistry Tools. No additional purification of reagents was performed prior to use.

Bacterial Culture Conditions

H. pylori strain G27 was streaked from frozen stocks onto horse blood agar plates (4% Columbia agar supplemented with 5% horse blood, 10 µg/mL vancomycin, 5 µg/mL cefsulodin, 0.3 µg/mL polymyxin B, 5 µg/mL trimethoprim, and 8 µg/mL amphotericin B) and incubated under microaerophilic conditions (14% CO_2, 37 °C) for 3–4 days. Liquid cultures were prepared in Brucella broth (pH 7.0) supplemented with 10% fetal bovine serum and 10 µg/mL vancomycin.

B. fragilis strain ATCC 23745 was cultivated on brain-heart infusion (BHI) agar plates (1.5% Bacto agar, 3.7% BHI medium, 0.5% yeast extract, 15 µg/mL porcine hematin) for one day under anaerobic conditions (Thermo Scientific Oxoid AnaeroGen sachet, 37 °C). Liquid cultures were prepared in BHI broth.

Metabolic Labeling of *H. pylori*

H. pylori grown on horse blood agar plates (4% Columbia agar supplemented with 5% horse blood, 10 µg/mL vancomycin, 5 µg/mL cefsulodin, 0.3 µg/mL polymyxin B, 5 µg/mL trimethoprim, and 8 µg/mL amphotericin B) were inoculated at an OD_{600} of 0.1–0.4 into Brucella broth supplemented with 0.5 mM peracetylated *N*-acetylglucosamine ($Ac_4GlcNAc$), 0.5 mM peracetylated *N*-azidoacetylglucosamine ($Ac_4GlcNAz$), or 0.5 mM $Ac_4GlcNAz$ alongside varying concentrations (0.01–2 mM) of O-naphthylmethyl and O-anthracenemethyl glycosides **6 – 11**. For a head-to-head comparison of FucNAcONAP (**8**) with the previously reported O-benzyl glycoside FucOBn (**4**), *H. pylori* were inoculated in Brucella broth supplemented with 0.5 mM $Ac_4GlcNAc$, 0.5 mM $Ac_4GlcNAz$, or 0.5 mM $Ac_4GlcNAz$ alongside varying concentrations (0.1–1 mM) of FucNAcONAP (**8**) and FucOBn (**4**). Cultures were incubated under microaerophilic conditions for 3-4 days, harvested by centrifugation at 3500 rpm (Eppendorf 5804R), and washed three times with phosphate-buffered saline (PBS).

SDS-PAGE and Western Blot Analysis

To detect azide-labeled glycans in cell lysates, washed metabolically labeled cells were incubated in lysis buffer (20 mM Tris-HCl, pH 7.4; 1% Igepal; 150 mM NaCl; 1 mM EDTA) containing a protease inhibitor cocktail (MilliporeSigma) for 30 minutes at -20°C. Lysates were clarified by centrifugation at 10,000 × *g* using a microcentrifuge. *B. fragilis* lysates underwent additional freeze-thaw and sonication (1.9 L ultrasonic bath, Fisher Scientific) for 20 minutes at room temperature. Protein concentrations of the supernatant were determined using the DC Protein Assay (Bio-Rad) and standardized to ~2.5 mg/mL. Standardized samples were incubated 1:1 (v/v) with 500 µM Phos-FLAG overnight at room temperature with shaking, followed by mixing 1:1 (v/v) with 2x SDS reducing loading buffer and boiling at 95°C for 5–10 minutes. EZ-Run protein ladder (15 µg, Fisher Scientific) and protein samples (37.5 µg) were loaded into Bio-Rad 12% Tris-Glycine or (TGX™) SDS-PAGE gels with a 4% stacking layer and electrophoresed at 200 V for 45-60 minutes with 1X SDS running buffer (H_2O, 3.47 mM SDS, 24.71 mM Tris-base, 191.95 mM glycine) in a Mini PROTEAN Tetra Cell (Bio-Rad). Electrophoresed gels were subsequently transferred to nitrocellulose membranes (Amersham™ Protran™ 0.45 NC) at 100 V for 1 hour or stained with Coomassie brilliant blue (Stain: 0.25% of Coomassie brilliant blue in 45% water, 45% methanol, 10% acetic acid/ Destain: 50% deionized water, 40% methanol, 10% acetic acid,) to visualize protein loading. Immunoblots were blocked with 5% non-fat dry milk in 0.05% TBS-T buffer (5 mM Tris-HCl, 0.05% Tween-20, pH 7.4) for 1 hour. Detection was performed using anti-FLAG-HRP (MilliporeSigma); 1:1000 dilution in TBS-T) and chemiluminescence (SuperSignal West Pico, Thermo Fisher Scientific) was visualized using a G:BOX Chemi XRQ system (Syngene).

Lectin Binding Flow Cytometry

H. pylori were cultured in rich liquid media (Brucella Broth) for 3 days treated with or without O-naphthylmethyl glycosides (0.1–1.0 mM) **6 – 8**. After incubation, cells were harvested, washed in PBS, and incubated with Alexa Fluor 488-conjugated Concanavalin A (ConA; 15 µg/mL; Thermo Fisher Scientific, λ_{ex} 488 nm, λ_{em} 519 nm) at 37°C under microaerophilic conditions for 60 minutes. Control samples were pre-incubated with 400 mM mannose for 60 minutes at 37 °C prior to lectin labeling. After washing three times with PBS, cells were analyzed on a BD Accuri C6$^+$ flow cytometer (BD Biosciences), with 10,000 live cells gated for each sample. Data were analyzed using FlowJo software (Ashland, OR). The labeling experiment was performed in triplicates and the results were expressed as number of cells versus fluorescence intensity in histogram plots. Alternatively, to compliment the histograms, results were reported as mean fluorescence intensity (MFI) values of a population of cells of each replicate.

Growth Curve Analysis of *H. pylori*

H. pylori were grown on horse blood agar plates (4% Columbia agar supplemented with 5% horse blood, 10 µg/mL vancomycin, 5 µg/mL cefsulodin, 0.3 µg/mL polymyxin B, 5 µg/mL trimethoprim, and 8 µg/mL amphotericin B) and subcultured into Brucella broth (10% fetal bovine

serum and 10 μg/mL) at $OD_{600} \approx 0.1$ with or without O-naphthylmethyl glycosides (0.1–1.0 mM) **6 – 8**. Cells were incubated under microaerophilic conditions with gentle shaking, and OD_{600} readings were recorded daily using a SPECTROStar Nano plate reader (Thermo Fisher Scientific) for up ten days.

Viability Assays

H. pylori were grown on horse blood agar plates (4% Columbia agar supplemented with 5% horse blood, 10 μg/mL vancomycin, 5 μg/mL cefsulodin, 0.3 μg/mL polymyxin B, 5 μg/mL trimethoprim, and 8 μg/mL amphotericin B) and subcultured into Brucella broth (10% fetal bovine serum and 10 μg/mL) at $OD_{600} \approx 0.4$. After standardization, cells were treated with 0.1–10 mM O-naphthylmethyl glycosides **6 – 8** or left untreated. Viability was assessed at the start of the experiment (day 0) and after 4 days (day 4). The LIVE/DEAD BacLight Bacterial Viability and Counting Kit (Invitrogen) was used according to manufacturer's instructions, followed flow cytometry analysis of 10,000 gated cells for each replicate (BD Accuri $C6^{+}$). Live and dead controls were prepared by suspending cells in 0.85% NaCl or 70% isopropanol, respectively, for 5 minutes at 37°C, following a rinse with 0.85% NaCl before straining with LIVE/DEAD BacLight Bacterial Viability and Counting Kit (Invitrogen). The percentage of live cells was calculated as:

$$\% \text{ Live} = 100 \times [\# \text{ live cells} / (\# \text{ live cells} + \# \text{ dead cells})].$$

Motility Assays

H. pylori cultures were standardized to $OD_{600} = 0.3–0.4$ in brucella broth before being treated with or without O-naphthylmethyl glycosides (0.1 mM – 1.0 mM) **6 – 8** under microaerophilic condition for one day. Cells were then concentrated by centrifugation, resuspended in 60 μL Brucella broth, and inoculated onto soft agar plates (Brucella broth with 10% fetal bovine serum (v/v), 6 μg/mL vancomycin, and 0.4% Difco agar). Plates were incubated under microaerophilic conditions, and colony diameters were measured daily for 10 days.

Biofilm Formation Assays

Biofilm formation by *H. pylori* was assessed in the outer edges of 96-well flat bottom plates following O'Toole's protocol.[3] Cultures ($OD_{600} = 0.3–0.4$) were incubated with or without O-naphthylmethyl glycosides (0.1–1.0 mM) **6 – 8** for 3-4 days. Bacterial cultures were carefully removed, carefully rinsed, and the biofilms were stained with 0.15% crystal violet and photographed. To quantitatively assess the biofilm formation, the biofilm was destained with 30% acetic acid and the absorbance was measured at 562 nm (SPECTROStar Nano, Thermo Fisher Scientific). Relative biofilm formation (%) was calculated as: 100 × [(A_{562} of biofilm from treated *H. pylori*)/(A_{562} of biofilm untreated *H. pylori*)].

Metabolic Labeling of *B. fragilis*

B. fragilis cultures were grown anaerobically on BHI agar plate (1.5% Bacto agar, 3.7% BHI medium, 0.5% yeast extract, 15 µg/mL porcine hematin), then inoculated from plates into BHI broth (OD_{600} = 0.1–0.4) supplemented with 0.5 mM Ac_4GlcNAc, 0.5 mM Ac_4GalNAz, or 0.5 mM Ac_4GalNAz and varying concentrations (0.01–0.25 mM) of FucNAcONAP. After 2 days of incubation, cultures were harvested and washed as above.

Growth Curve Analysis of *B. fragilis*

B. fragilis cultures (OD_{600} ≈ 0.1) in BHI broth with or without 0.25 mM FucNAcONAP were incubated under anaerobic conditions with shaking, and OD_{600} readings were recorded daily using a SPECTROStar Nano plate reader (Thermo Fisher Scientific) for up to ten days.

Crude LPS isolation and Pro-Q Emerald polysaccharide staining

H. pylori were grown on HBA plates as described above. After three days of incubation, bacterial cells were rinsed in 1X PBS, centrifuged at 3500 x *g* for 15 minutes, and the pellets were lysed with lysis buffer (20 mM Tris-HCl, pH 7.4; 1% Igepal; 150 mM NaCl; 1 mM EDTA) containing protease inhibitor cocktail (MilliporeSigma) at room temperature to yield clarified lysates. Soluble contents were obtained by centrifugation at 10,000 x *g* for 10 minutes.

To selectively isolate crude LPS from *H. pylori,* pellets and supernatant were treated with LPS lysis buffer (1:1 v/v) (4 mL 10% SDS, 800 µL β-mercaptoethanol, 1.2 mg bromophenol blue, 2 mL glycerol, 10 mL 1.5 M Tris-HCl, 5 mL H_2O) and heated at 100°C for 10 minutes. After heating, samples were treated with Proteinase K (20 mg/mL, 1:30 v/v) and incubated at 55 °C overnight.

Crude LPS samples from the pellet and supernatant were boiled at 95 °C for 10 minutes before loading onto a Bio-Rad 4%–20% Tris-TGX^{TM} polyacrylamide gel with a 4% stacking layer. A 10 µL molecular weight ladder (EZ-Run Prestained Rec Protein Ladder), 10 µL CandyCane™ glycoprotein molecular weight standard (1:6 v/v of 5 mg/mL CandyCane™ stock solution) (Thermo Fisher Scientific), and 10 µL LPS standard (MCE HY-D1056, 2 mg/mL) were loaded alongside the samples. The gel was electrophoresed for 50 minutes at 200 V in 1X SDS running buffer (H_2O, 3.47 mM SDS, 24.71 mM Tris base, 191.95 mM glycine) in a Mini PROTEAN Tetra Cell (Bio-Rad, Hercules, CA). Following electrophoresis, the gel was stained using the Pro-Q™ Emerald 300 Glycoprotein Stain Kit (Thermo Fisher Scientific) according to the manufacturer's instructions: The gel was fixed twice for 45 minutes each in a fix solution (50% ethanol, 5% acetic acid), washed twice in a wash solution containing 3% acetic acid. The carbohydrates in the gel were oxidized for 30 minutes in an oxidizing solution (0.04 M periodic acid in wash solution). The gel was washed three times before being incubated in a staining solution (500 µL of Pro-Q® Emerald 300 stock solution and 25 mL of Pro-Q® Emerald 300 staining buffer) for 100 minutes

in complete darkness. The gel was then washed twice with wash solution before viewing the stained polysaccharides using a 300 nm UV transilluminator.

Supplemental Figures

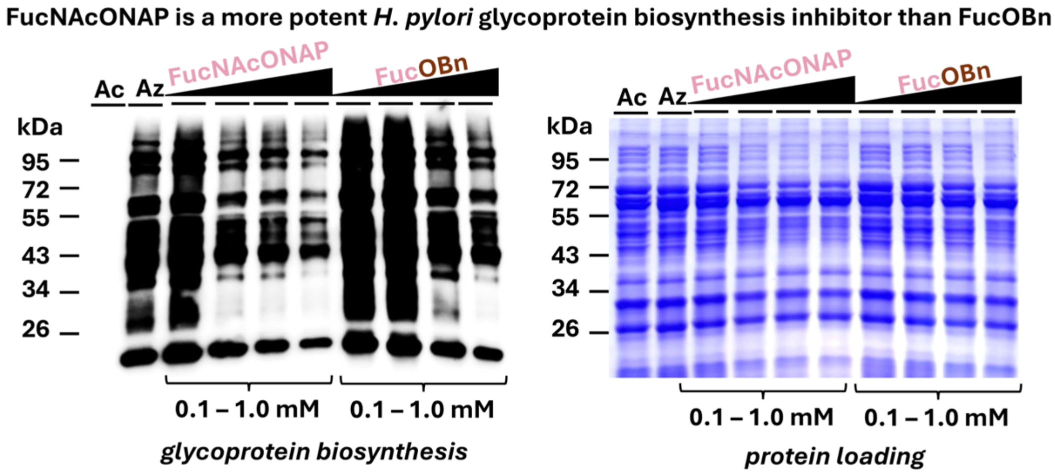

Figure S1. A head-to-head comparison of *H. pylori* glycoprotein biosynthesis following treatment with FucNAcONAP (**8**) or FucOBn (**4**) at a range of concentrations (0.1, 0.25, 0.5, 1.0 mM) revealed that FucNAcONAP is more potent. Coomassie staining of *H. pylori* samples treated with FucNAcONAP (**8**) or FucOBn (**4**) at a range of concentrations (0.1, 0.25, 0.5, 1.0 mM) confirmed uniform protein loading across samples.

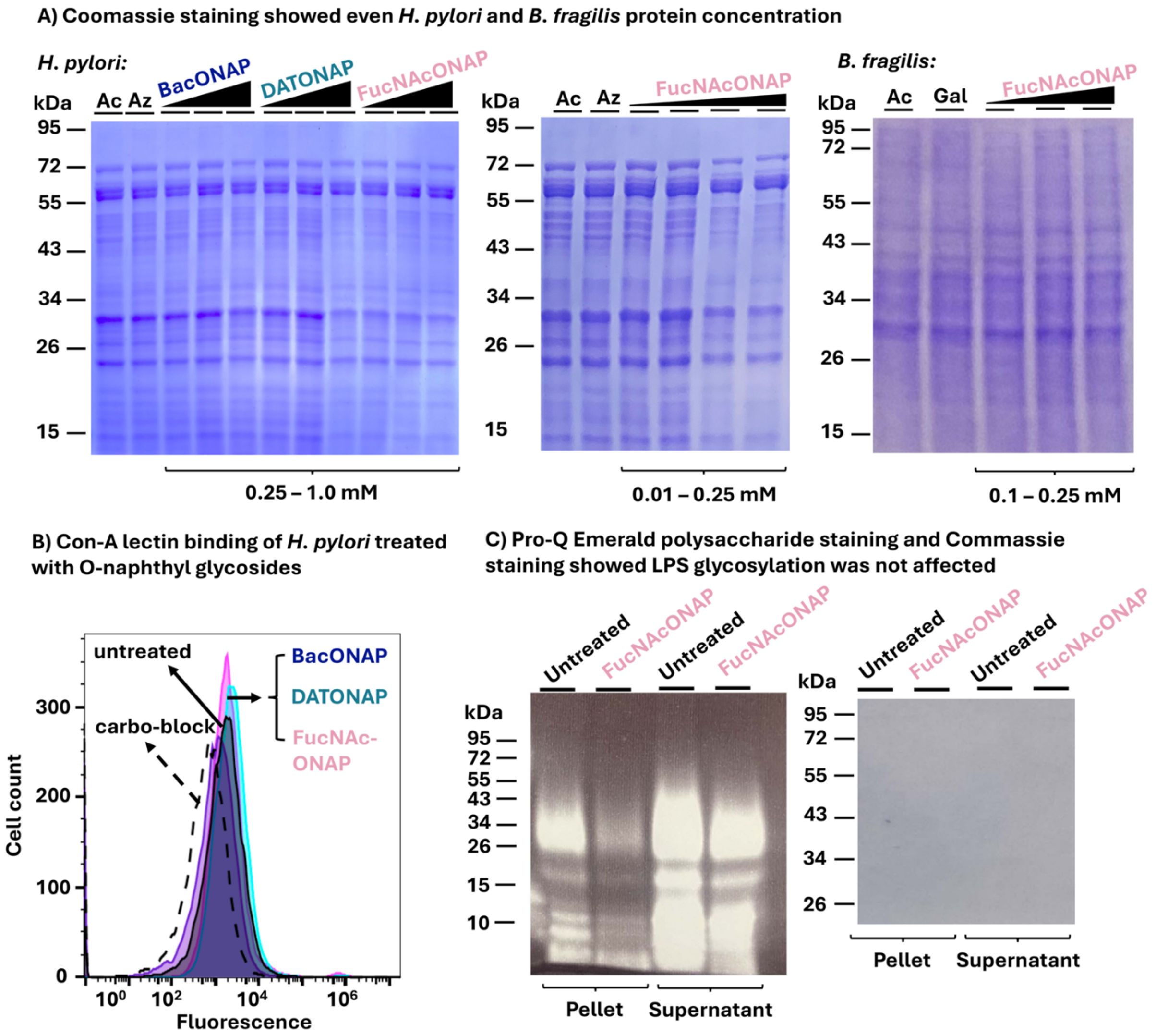

Figure S2. (A) Coomassie staining of *H. pylori* and *B. fragilis* treated with *O-naphthylmethyl* glycosides confirmed uniform protein concentration across samples. **(B)** Flow cytometry histogram showing a shift in ConA fluorescence intensity in *H. pylori* treated with *O*-naphthylmethyl glycosides compared to the untreated control, indicating alterations in bacterial surface glycan architecture. **(C)** Pro-Q Emerald polysaccharide staining of *H. pylori* lipopolysaccharide (LPS) following FucNAcONAP treatment demonstrated that FucNAcONAP did not disrupt LPS glycosylation. Coomassie staining confirmed the absence of protein contamination in the loaded samples.

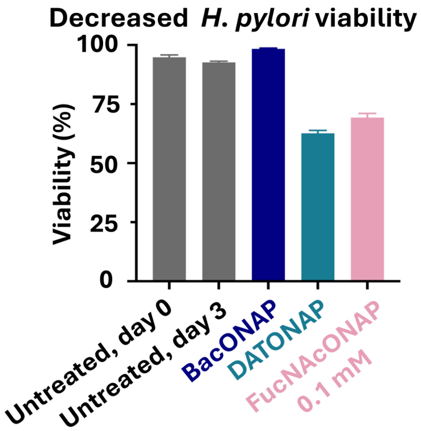

Figure S3. DATONAP and FucNAcONAP impacted *H. pylori* viability. Live/Dead scoring of viability revealed that 1.0 mM DATONAP (**7**) and 0.1 mM FucNAcONAP (**8**) led to decreased viability relative to untreated *H. pylori*, whereas BacONAP (**6**) did not significantly influence viability.

Supplemental References

(1) Williams, D. A.; Pradhan, K.; Paul, A.; Olin, I. R.; Tuck, O. T.; Moulton, K. D.; Kulkarni, S. S.; Dube, D. H. Metabolic inhibitors of bacterial glycan biosynthesis. *Chem. Sci.* **2020**, *11* (7), 1761–1774. https://doi.org/10.1039/C9SC05955E.

(2) Baltrus, D. A.; Amieva, M. R.; Covacci, A.; Lowe, T. M.; Merrell, D. S.; Ottemann, K. M.; Stein, M.; Salama, N. R.; Guillemin, K. The complete genome sequence of *Helicobacter pylori* strain G27. *J. Bacteriol.* **2009**, *191* (1), 447–448. https://doi.org/10.1128/JB.01416-08.

(3) O'Toole, G. A. Microtiter dish biofilm formation assay. *J. Vis. Exp. JoVE* **2011**, No. 47, 2437. https://doi.org/10.3791/2437.

SSK-23-AP-1265-1H

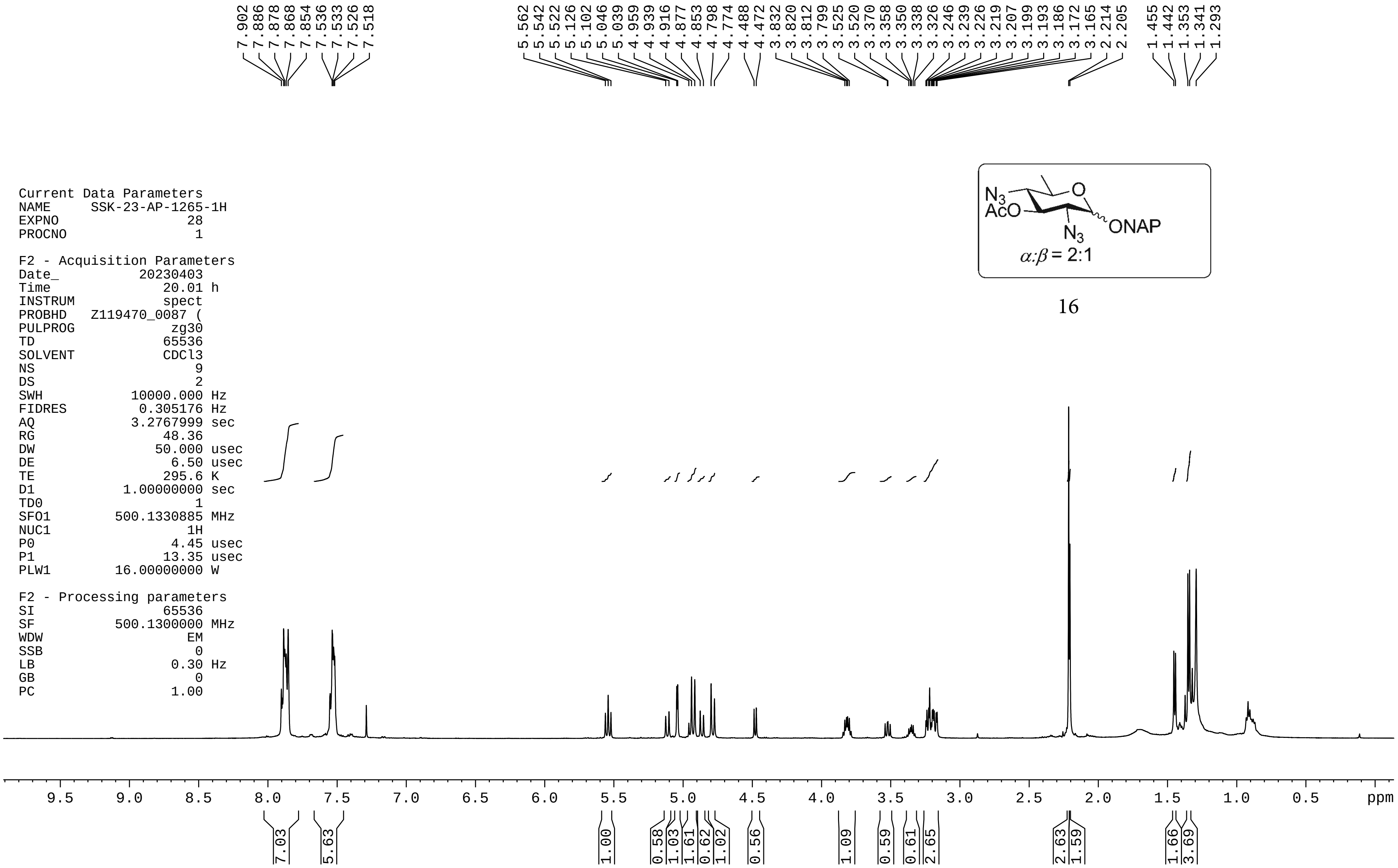

Current Data Parameters
NAME SSK-23-AP-1265-1H
EXPNO 28
PROCNO 1

F2 - Acquisition Parameters
Date_ 20230403
Time 20.01 h
INSTRUM spect
PROBHD Z119470_0087 (
PULPROG zg30
TD 65536
SOLVENT CDCl3
NS 9
DS 2
SWH 10000.000 Hz
FIDRES 0.305176 Hz
AQ 3.2767999 sec
RG 48.36
DW 50.000 usec
DE 6.50 usec
TE 295.6 K
D1 1.00000000 sec
TD0 1
SFO1 500.1330885 MHz
NUC1 1H
P0 4.45 usec
P1 13.35 usec
PLW1 16.00000000 W

F2 - Processing parameters
SI 65536
SF 500.1300000 MHz
WDW EM
SSB 0
LB 0.30 Hz
GB 0
PC 1.00

α:β = 2:1

16

SSK-23-AP-1265-13C

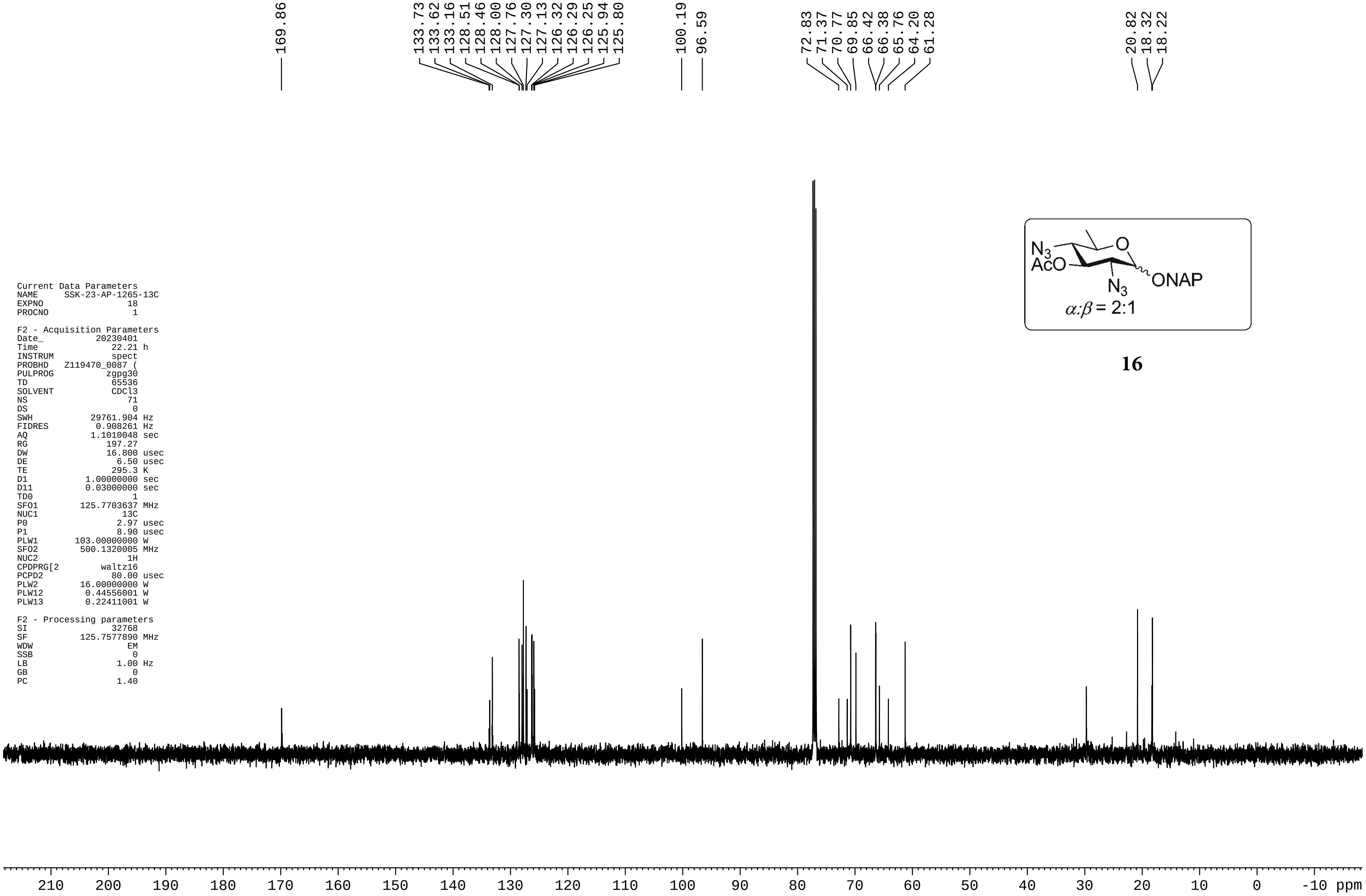


```
Current Data Parameters
NAME      SSK-23-AP-1265-13C
EXPNO                   18
PROCNO                   1

F2 - Acquisition Parameters
Date_           20230401
Time               22.21 h
INSTRUM            spect
PROBHD   Z119470_0087 (
PULPROG           zgpg30
TD                 65536
SOLVENT            CDCl3
NS                    71
DS                     0
SWH            29761.904 Hz
FIDRES          0.908261 Hz
AQ             1.1010048 sec
RG                197.27
DW                16.800 usec
DE                  6.50 usec
TE                 295.3 K
D1            1.00000000 sec
D11           0.03000000 sec
TD0                    1
SFO1         125.7703637 MHz
NUC1                 13C
P0                  2.97 usec
P1                  8.90 usec
PLW1        103.00000000 W
SFO2         500.1320005 MHz
NUC2                  1H
CPDPRG[2         waltz16
PCPD2              80.00 usec
PLW2         16.00000000 W
PLW12         0.44556001 W
PLW13         0.22411001 W

F2 - Processing parameters
SI                 32768
SF           125.7577890 MHz
WDW                   EM
SSB                    0
LB                  1.00 Hz
GB                     0
PC                  1.40
```


16

SSK-23-AP-1265-DEPT

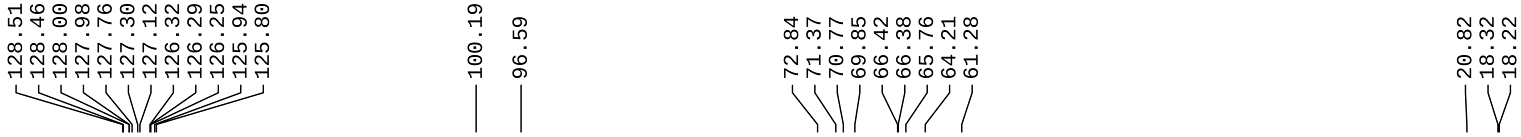

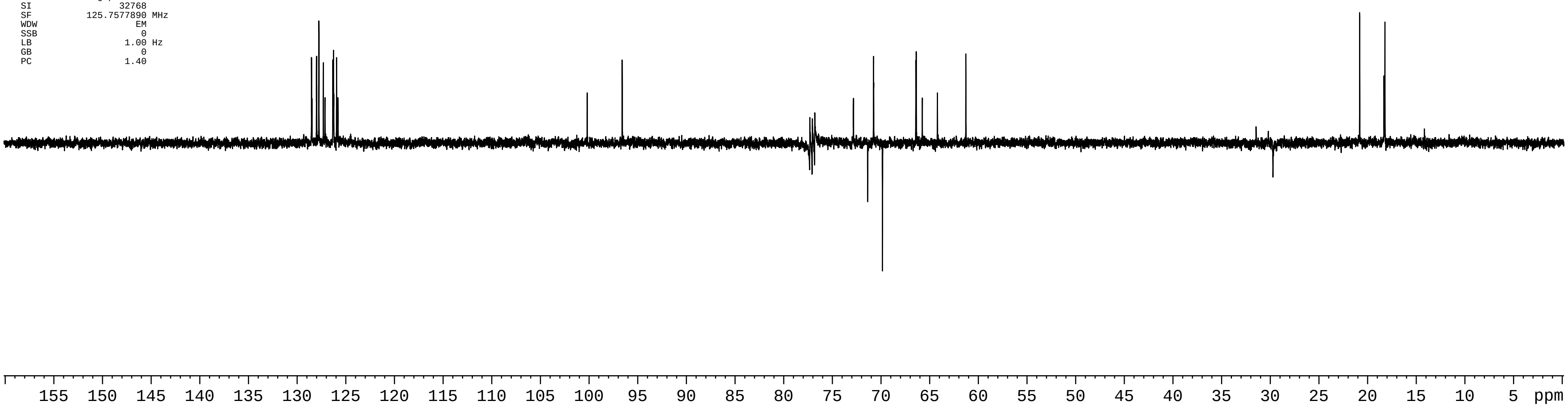


```
Current Data Parameters
NAME     SSK-23-AP-1265-DEPT
EXPNO                  29
PROCNO                  1

F2 - Acquisition Parameters
Date_          20230403
Time              20.03 h
INSTRUM           spect
PROBHD   Z119470_0087 (
PULPROG       deptsp135
TD                65536
SOLVENT           CDCl3
NS                   23
DS                    0
SWH           20161.291 Hz
FIDRES         0.615274 Hz
AQ            1.6252928 sec
RG               197.27
DW               24.800 usec
DE                 6.50 usec
TE                295.6 K
CNST2       145.0000000
D1           2.00000000 sec
D2           0.00344828 sec
D12          0.00002000 sec
TD0                   1
SFO1        125.7678486 MHz
NUC1                13C
P1                 8.90 usec
P13             2000.00 usec
PLW0                  0 W
PLW1       103.00000000 W
SPNAM[5]    Crp60comp.4
SPOAL5            0.500
SPOFFS5               0 Hz
SPW5        12.46500015 W
SFO2        500.1315995 MHz
NUC2                 1H
CPDPRG[2        waltz16
P3                13.35 usec
P4                26.70 usec
PCPD2             80.00 usec
PLW2        16.00000000 W
PLW12        0.44556001 W

F2 - Processing parameters
SI                32768
SF          125.7577890 MHz
WDW                  EM
SSB                   0
LB                 1.00 Hz
GB                    0
PC                 1.40
```

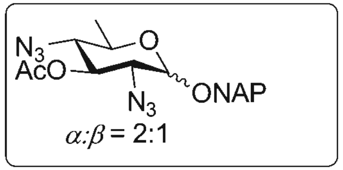

16

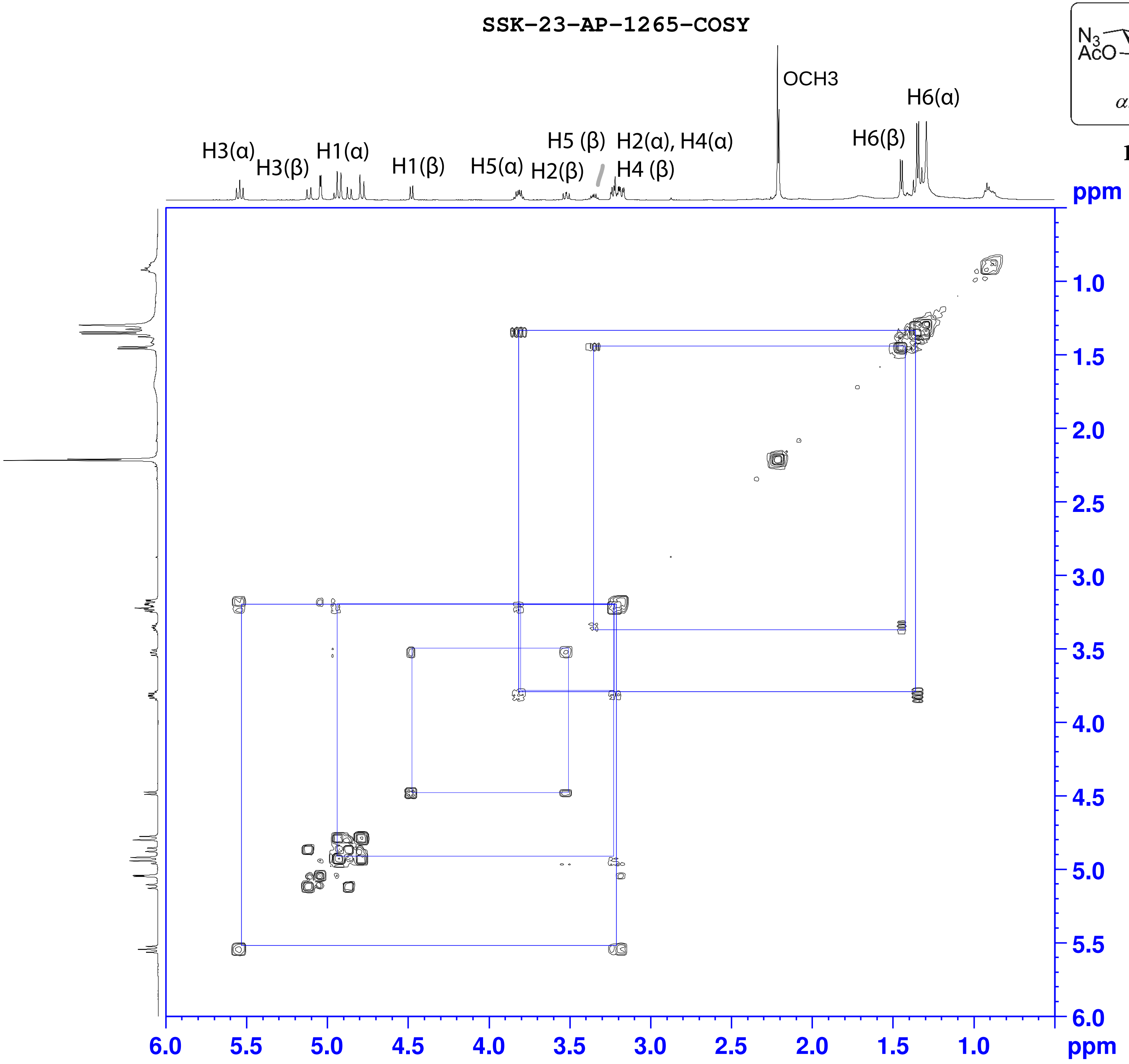

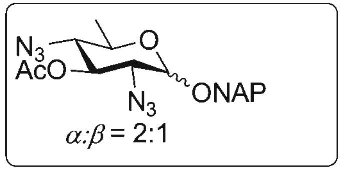

16

Current Data Parameters
NAME SSK-23-AP-1265-COSY
EXPNO 30
PROCNO 1

F2 - Acquisition Parameters
Date_ 20230403
Time 20.08 h
INSTRUM spect
PROBHD Z119470_0087 (
PULPROG cosygpppqf
TD 2048
SOLVENT CDCl3
NS 2
DS 0
SWH 6684.492 Hz
FIDRES 6.527824 Hz
AQ 0.1531904 sec
RG 61.42
DW 74.800 usec
DE 6.50 usec
TE 295.9 K
D0 0.00000300 sec
D1 1.00000000 sec
D11 0.03000000 sec
D12 0.00002000 sec
D13 0.00000400 sec
D16 0.00020000 sec
IN0 0.00014960 sec
TDav 1
SFO1 500.1330069 MHz
NUC1 1H
P0 13.35 usec
P1 13.35 usec
P17 2500.00 usec
PLW1 16.00000000 W
PLW10 3.16840005 W
GPNAM[1] SMSQ10.100
GPZ1 10.00 %
P16 1000.00 usec

F1 - Acquisition parameters
TD 128
SFO1 500.133 MHz
FIDRES 104.445190 Hz
SW 13.365 ppm
FnMODE QF

F2 - Processing parameters
SI 1024
SF 500.1300000 MHz
WDW QSINE
SSB 0
LB 0 Hz
GB 0
PC 1.40

F1 - Processing parameters
SI 1024
MC2 QF
SF 500.1300000 MHz
WDW QSINE
SSB 0
LB 0 Hz
GB 0

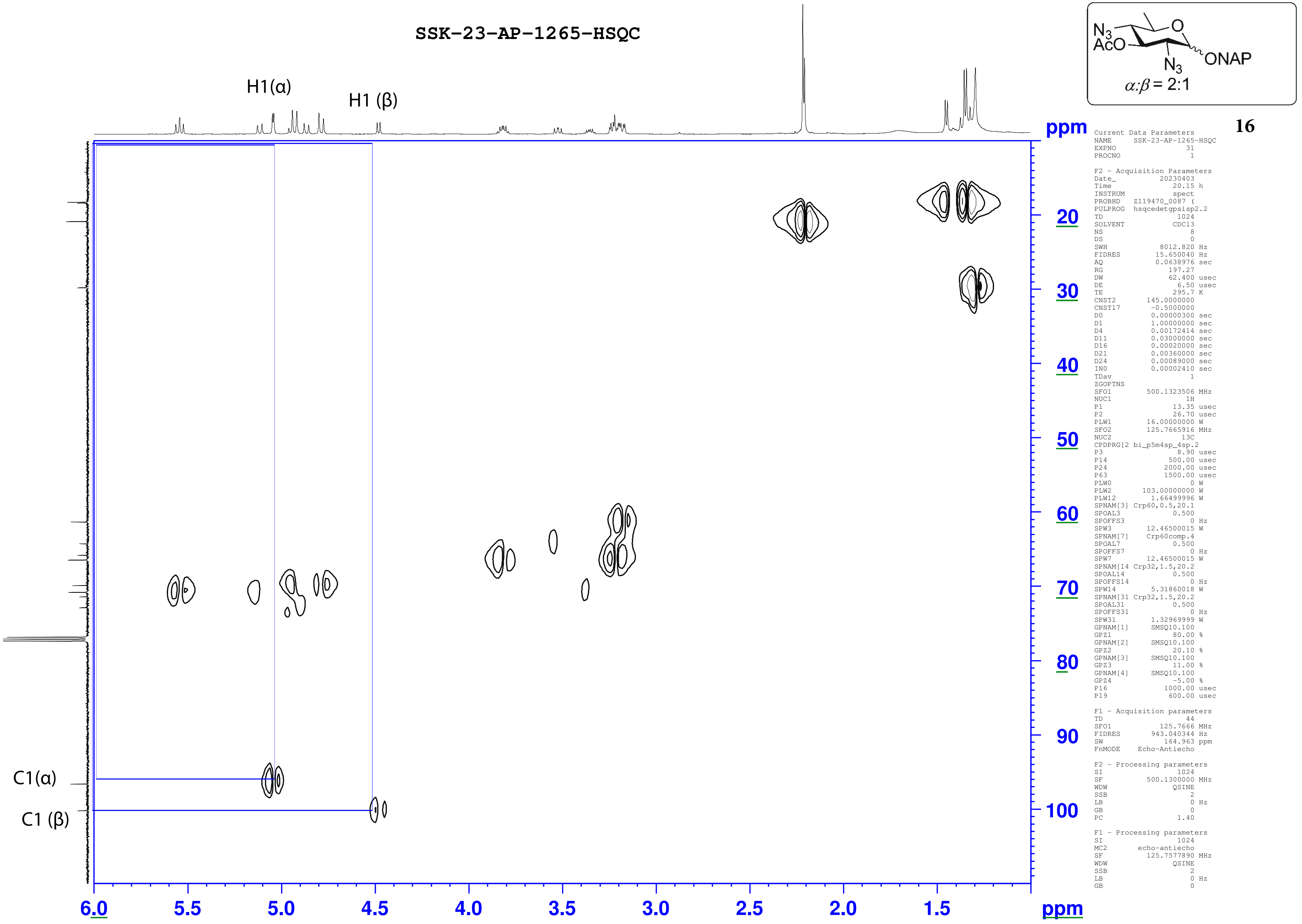

SSK-23-AP-1265-HSQC
H1(α)
H1 (β)
C1(α)
C1 (β)
ppm
20
30
40
50
60
70
80
90
100
6.0
5.5
5.0
4.5
4.0
3.5
3.0
2.5
2.0
1.5
ppm
N3
AcO
N3
O
ONAP
α:β = 2:1
16
Current Data Parameters
NAME SSK-23-AP-1265-HSQC
EXPNO 31
PROCNO 1
F2 - Acquisition Parameters
Date_ 20230403
Time 20.15 h
INSTRUM spect
PROBHD Z119470_0087 (
PULPROG hsqcedetgpsisp2.2
TD 1024
SOLVENT CDCl3
NS 8
DS 0
SWH 8012.820 Hz
FIDRES 15.650040 Hz
AQ 0.0638976 sec
RG 197.27
DW 62.400 usec
DE 6.50 usec
TE 295.7 K
CNST2 145.0000000
CNST17 -0.5000000
D0 0.00000300 sec
D1 1.00000000 sec
D4 0.00172414 sec
D11 0.03000000 sec
D16 0.00020000 sec
D21 0.00360000 sec
D24 0.00089000 sec
IN0 0.00002410 sec
TDav 1
ZGOPTNS
SFO1 500.1323506 MHz
NUC1 1H
P1 13.35 usec
P2 26.70 usec
PLW1 16.00000000 W
SFO2 125.7665916 MHz
NUC2 13C
CPDPRG[2 bi_p5m4sp_4sp.2
P3 8.90 usec
P14 500.00 usec
P24 2000.00 usec
P63 1500.00 usec
PLW0 0 W
PLW2 103.00000000 W
PLW12 1.66499996 W
SPNAM[3] Crp60,0.5,20.1
SPOAL3 0.500
SPOFFS3 0 Hz
SPW3 12.46500015 W
SPNAM[7] Crp60comp.4
SPOAL7 0.500
SPOFFS7 0 Hz
SPW7 12.46500015 W
SPNAM[14 Crp32,1.5,20.2
SPOAL14 0.500
SPOFFS14 0 Hz
SPW14 5.31860018 W
SPNAM[31 Crp32,1.5,20.2
SPOAL31 0.500
SPOFFS31 0 Hz
SPW31 1.32969999 W
GPNAM[1] SMSQ10.100
GPZ1 80.00 %
GPNAM[2] SMSQ10.100
GPZ2 20.10 %
GPNAM[3] SMSQ10.100
GPZ3 11.00 %
GPNAM[4] SMSQ10.100
GPZ4 -5.00 %
P16 1000.00 usec
P19 600.00 usec
F1 - Acquisition parameters
TD 44
SFO1 125.7666 MHz
FIDRES 943.040344 Hz
SW 164.963 ppm
FnMODE Echo-Antiecho
F2 - Processing parameters
SI 1024
SF 500.1300000 MHz
WDW QSINE
SSB 2
LB 0 Hz
GB 0
PC 1.40
F1 - Processing parameters
SI 1024
MC2 echo-antiecho
SF 125.7577890 MHz
WDW QSINE
SSB 2
LB 0 Hz
GB 0

SSK-23-AP-1267-1H

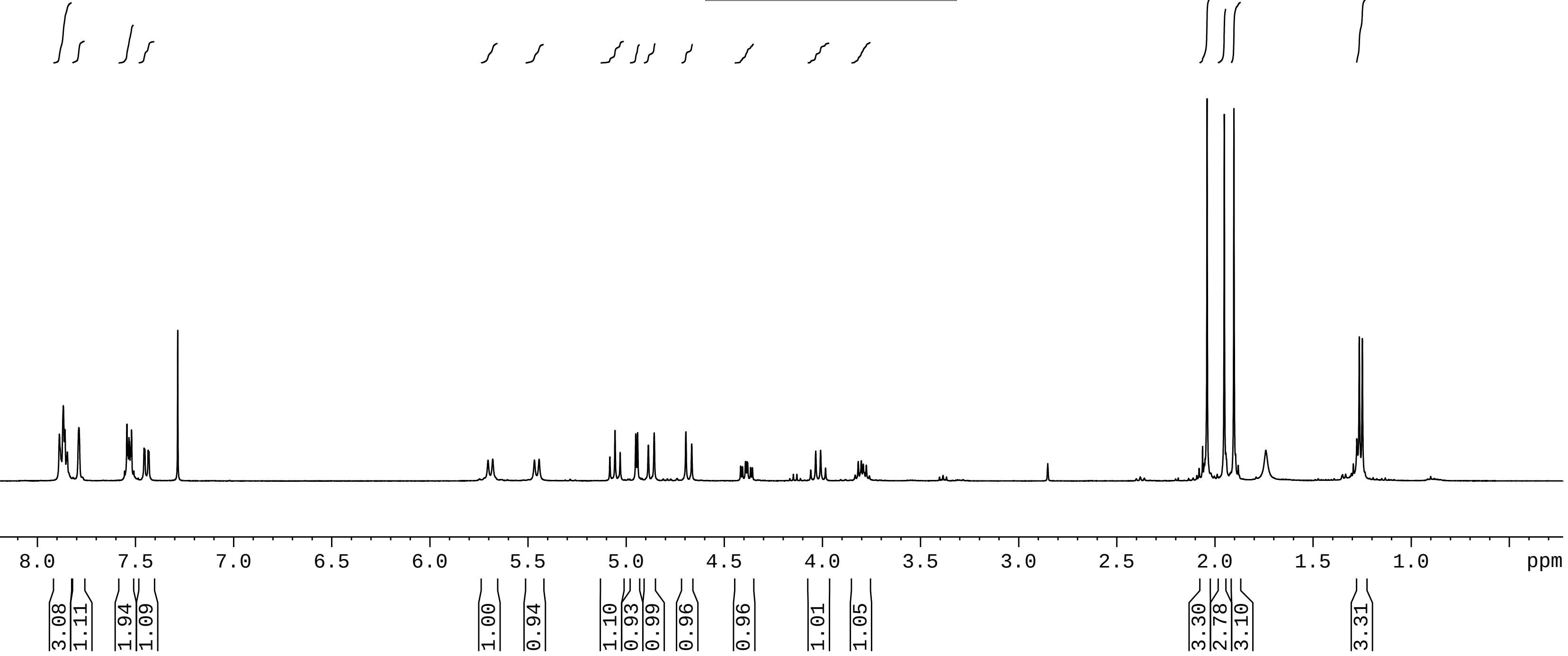

Current Data Parameters
NAME SSK-23-AP-1267-1H
EXPNO 10
PROCNO 1

F2 - Acquisition Parameters
Date_ 20230407
Time 15.57 h
INSTRUM Avance Neo 400
PROBHD Z163739_0226 (
PULPROG zg30
TD 51724
SOLVENT CDCl3
NS 9
DS 0
SWH 8620.689 Hz
FIDRES 0.333334 Hz
AQ 2.9999919 sec
RG 101
DW 58.000 usec
DE 13.14 usec
TE 297.0 K
D1 1.00000000 sec
TD0 1
SFO1 400.1324708 MHz
NUC1 1H
P0 2.67 usec
P1 8.00 usec
PLW1 25.07999992 W

F2 - Processing parameters
SI 65536
SF 400.1300000 MHz
WDW EM
SSB 0
LB 0.30 Hz
GB 0
PC 1.00

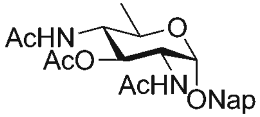

6

SSK-23-AP-1267-13C

```
Current Data Parameters
NAME     SSK-23-AP-1267-13C
EXPNO                  12
PROCNO                  1

F2 - Acquisition Parameters
Date_          20230407
Time              16.01 h
INSTRUM  Avance Neo 400
PROBHD   Z163739_0226 (
PULPROG          zgpg30
TD                65536
SOLVENT           CDCl3
NS                   86
DS                    2
SWH           27777.777 Hz
FIDRES         0.847710 Hz
AQ            1.1796480 sec
RG                  101
DW               18.000 usec
DE                 6.50 usec
TE                297.6 K
D1           1.00000000 sec
D11          0.03000000 sec
TD0                   1
SFO1        100.6242384 MHz
NUC1                13C
P0                 2.67 usec
P1                 8.00 usec
PLW1        99.33999634 W
SFO2        400.1316005 MHz
NUC2                 1H
CPDPRG[2        waltz65
PCPD2             90.00 usec
PLW2        25.07999992 W
PLW12        0.19815999 W
PLW13        0.09967500 W

F2 - Processing parameters
SI                32768
SF          100.6127685 MHz
WDW                  EM
SSB                   0
LB                 1.00 Hz
GB                    0
PC                 1.40
```

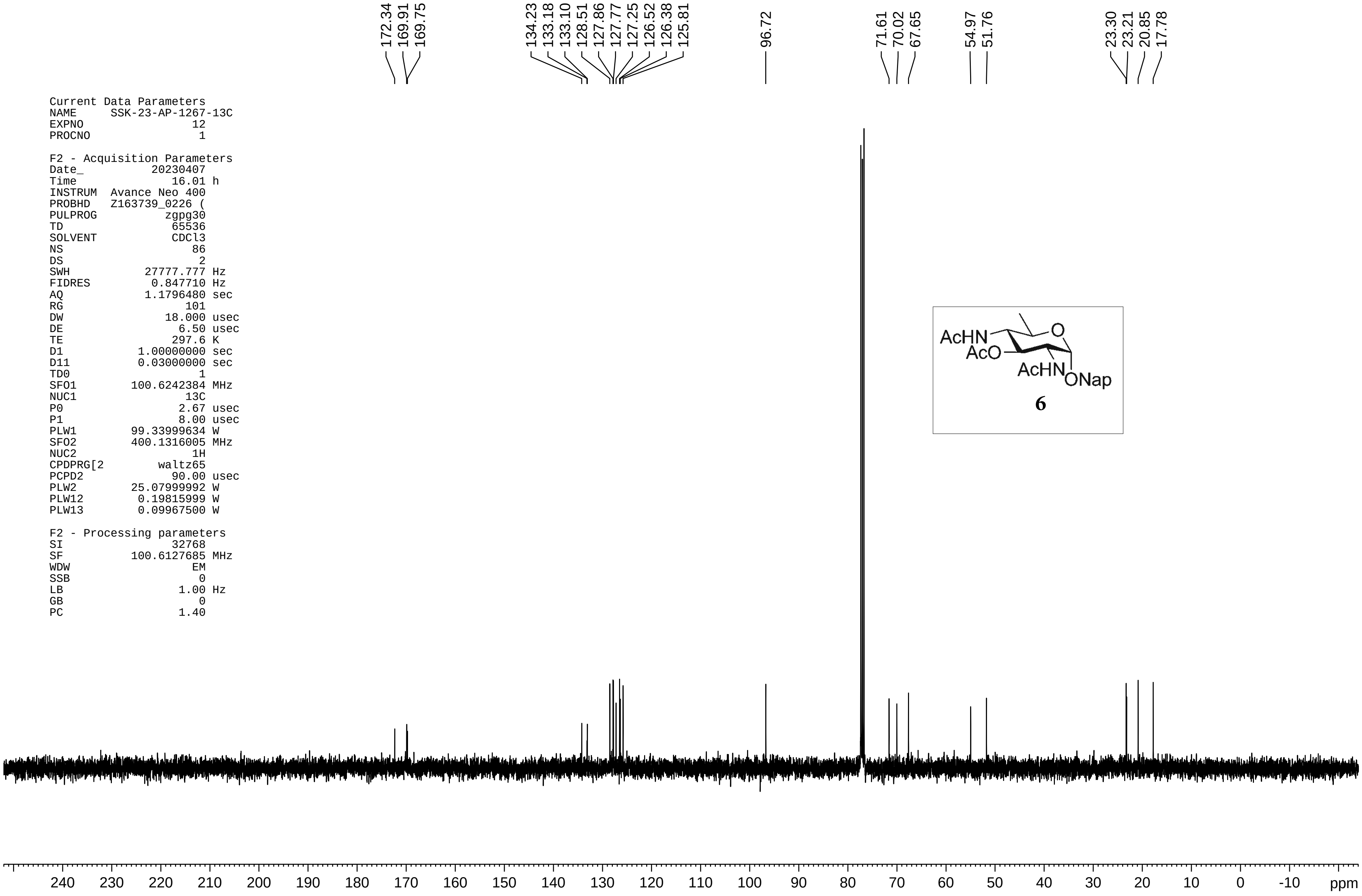

6

SSK-23-AP-1267-DEPT

Current Data Parameters
NAME SSK-23-AP-1267-DEPT
EXPNO 14
PROCNO 1

F2 - Acquisition Parameters
Date_ 20230407
Time 16.04 h
INSTRUM Avance Neo 400
PROBHD Z163739_0226 (
PULPROG deptsp135
TD 65536
SOLVENT CDCl3
NS 44
DS 4
SWH 27777.777 Hz
FIDRES 0.847710 Hz
AQ 1.1796480 sec
RG 101
DW 18.000 usec
DE 6.50 usec
TE 297.4 K
CNST2 145.0000000
D1 1.00000000 sec
D2 0.00344828 sec
D12 0.00002000 sec
TD0 1
SFO1 100.6242384 MHz
NUC1 13C
P1 8.00 usec
P13 2000.00 usec
PLW0 0 W
PLW1 99.33999634 W
SPNAM[5] Crp60comp.4
SPOAL5 0.500
SPOFFS5 0 Hz
SPW5 9.71399975 W
SFO2 400.1316005 MHz
NUC2 1H
CPDPRG[2 waltz65
P3 8.00 usec
P4 16.00 usec
PCPD2 90.00 usec
PLW2 25.07999992 W
PLW12 0.19815999 W

F2 - Processing parameters
SI 32768
SF 100.6127685 MHz
WDW EM
SSB 0
LB 1.00 Hz
GB 0
PC 1.40

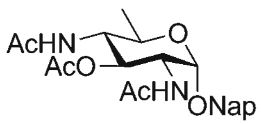

6

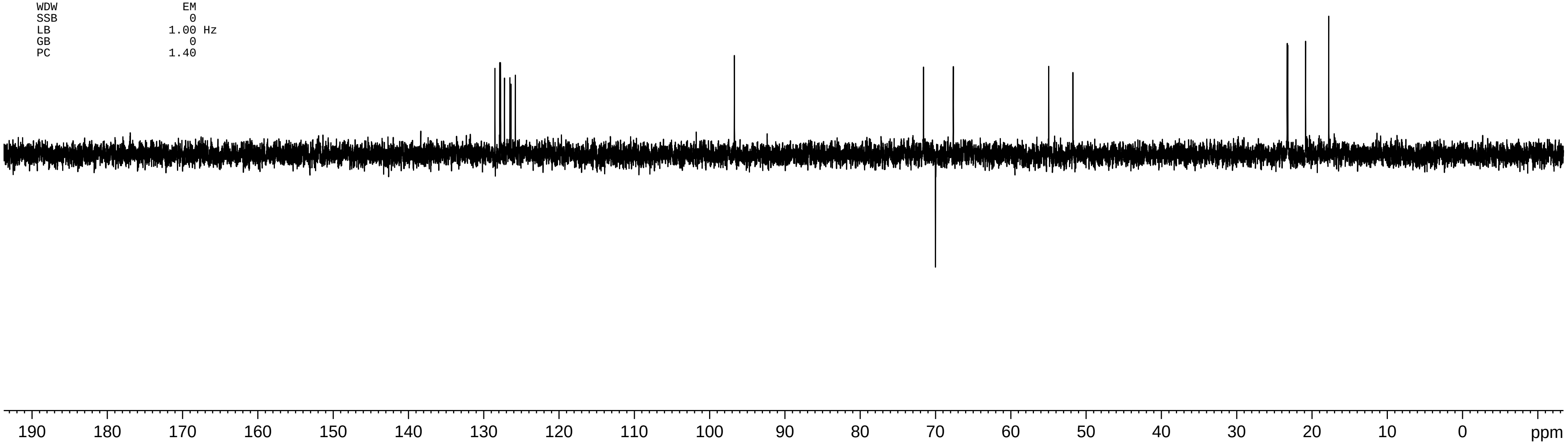

SSK-23-AP-1267-COSY

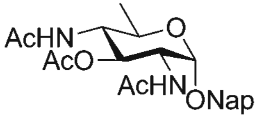

6

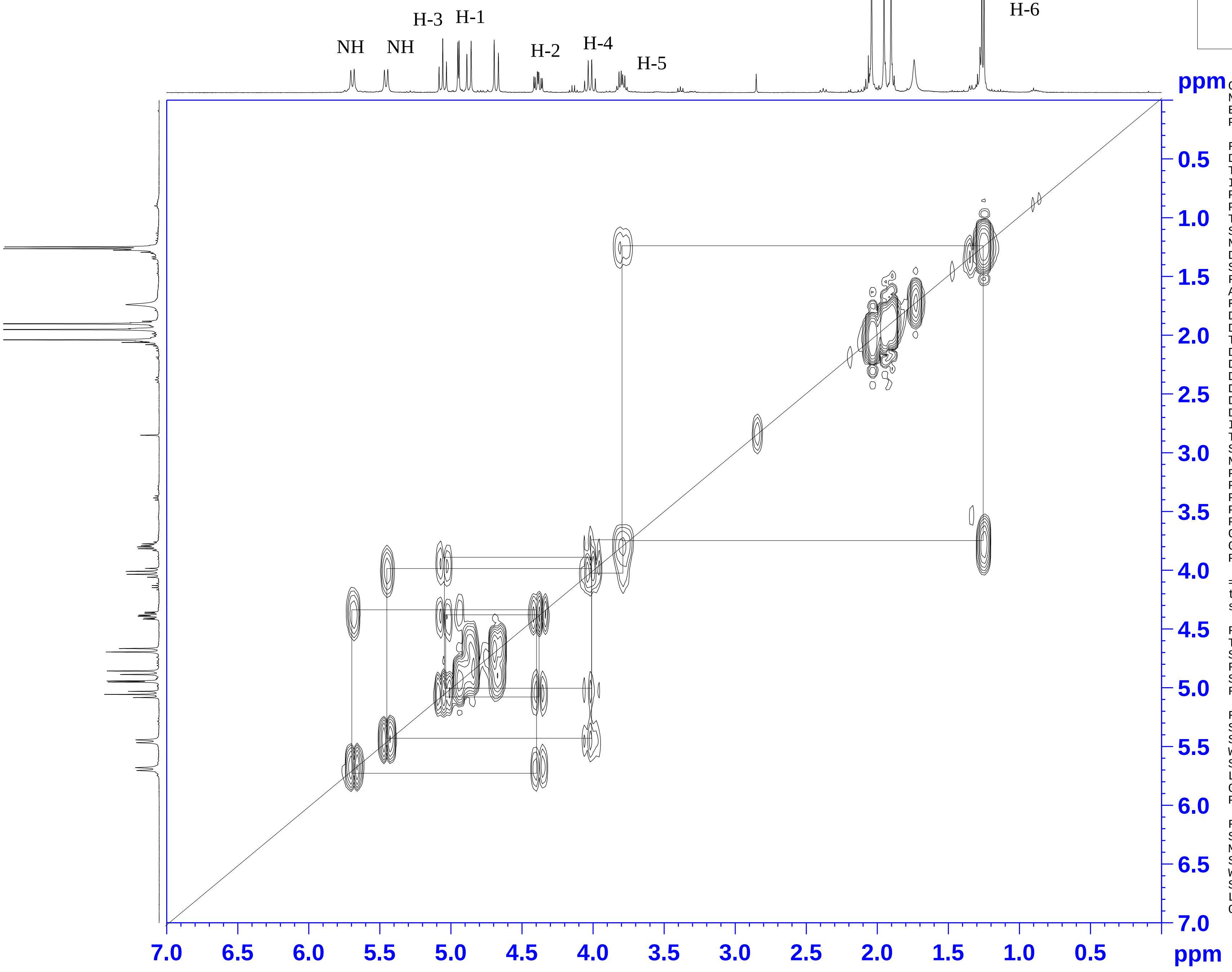


```
Current Data Parameters
NAME      SSK-23-AP-1267-COSY
EXPNO                      16
PROCNO                      1

F2 - Acquisition Parameters
Date_              20230407
Time                  16.12 h
INSTRUM   Avance Neo 400
PROBHD    Z163739_0226 (
PULPROG         cosygpppqf
TD                     2048
SOLVENT               CDCl3
NS                        4
DS                        0
SWH              8620.689 Hz
FIDRES           8.418642 Hz
AQ              0.1187840 sec
RG                      101
DW                   58.000 usec
DE                     6.50 usec
TE                    297.1 K
D0               0.00000300 sec
D1               1.00000000 sec
D11              0.03000000 sec
D12              0.00002000 sec
D13              0.00000400 sec
D16              0.00020000 sec
IN0              0.00011360 sec
TDav                      1
SFO1           400.1324708 MHz
NUC1                     1H
P0                     8.00 usec
P1                     8.00 usec
P17                 2500.00 usec
PLW1            25.07999992 W
PLW10            1.78349996 W
GPNAM[1]         SMSQ10.100
GPZ1                  10.00 %
P16                 1000.00 usec

======== F1 INDIRECT DIMENSION ========
td1        128
sw_F1      21.999996

F1 - Acquisition parameters
TD                       97
SFO1              400.1325 MHz
FIDRES          181.501373 Hz
SW                   22.000 ppm
FnMODE                   QF

F2 - Processing parameters
SI                     1024
SF              400.1300000 MHz
WDW                   QSINE
SSB                       0
LB                        0 Hz
GB                        0
PC                     1.40

F1 - Processing parameters
SI                     1024
MC2                      QF
SF              400.1300000 MHz
WDW                   QSINE
SSB                       0
LB                        0 Hz
GB                        0
```

SSK-23-AP-DAT-ONAP-1H

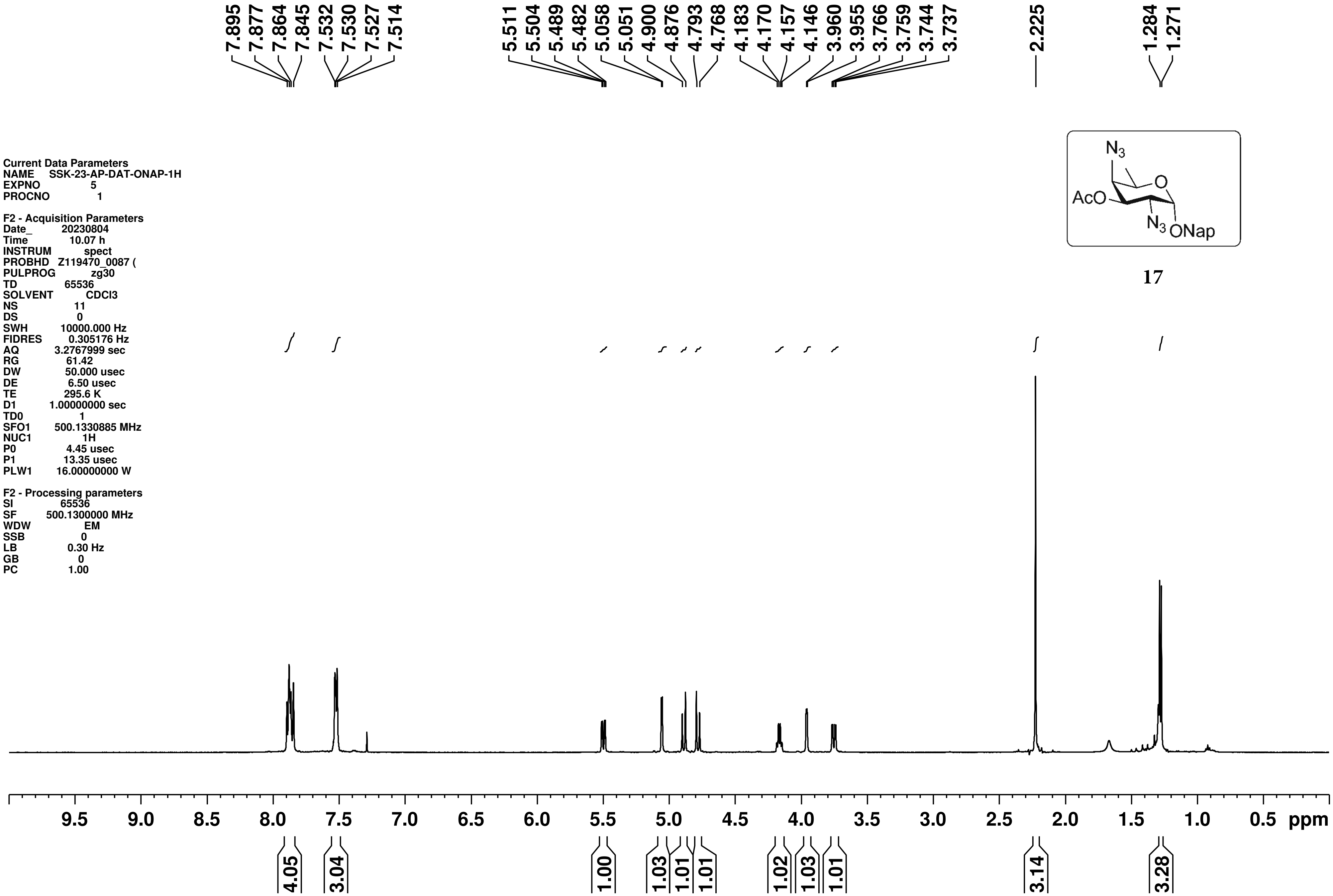

17

Current Data Parameters
NAME SSK-23-AP-DAT-ONAP-1H
EXPNO 5
PROCNO 1

F2 - Acquisition Parameters
Date_ 20230804
Time 10.07 h
INSTRUM spect
PROBHD Z119470_0087 (
PULPROG zg30
TD 65536
SOLVENT CDCl3
NS 11
DS 0
SWH 10000.000 Hz
FIDRES 0.305176 Hz
AQ 3.2767999 sec
RG 61.42
DW 50.000 usec
DE 6.50 usec
TE 295.6 K
D1 1.00000000 sec
TD0 1
SFO1 500.1330885 MHz
NUC1 1H
P0 4.45 usec
P1 13.35 usec
PLW1 16.00000000 W

F2 - Processing parameters
SI 65536
SF 500.1300000 MHz
WDW EM
SSB 0
LB 0.30 Hz
GB 0
PC 1.00

SSK-23-AP-DAT-ONAP-13C

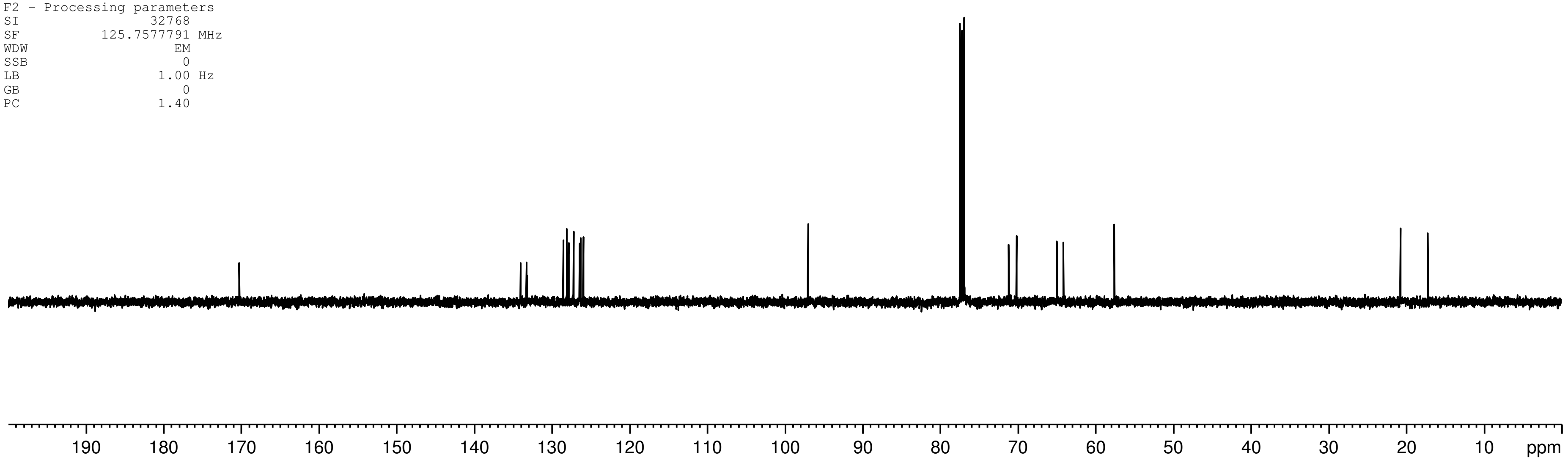


```
Current Data Parameters
NAME      SSK-23-AP-DAT-ONAP-13C
EXPNO                    6
PROCNO                   1

F2 - Acquisition Parameters
Date_             20230804
Time                 10.08 h
INSTRUM              spect
PROBHD   Z119470_0087 (
PULPROG            zgpg30
TD                   65536
SOLVENT              CDCl3
NS                      19
DS                       0
SWH              34722.223 Hz
FIDRES            1.059638 Hz
AQ               0.9437184 sec
RG                  197.27
DW                  14.400 usec
DE                    6.50 usec
TE                   296.0 K
D1              1.00000000 sec
D11             0.03000000 sec
TD0                      1
SFO1           125.7721254 MHz
NUC1                   13C
P0                    2.97 usec
P1                    8.90 usec
PLW1          103.00000000 W
SFO2           500.1320005 MHz
NUC2                    1H
CPDPRG[2           waltz16
PCPD2                80.00 usec
PLW2           16.00000000 W
PLW12           0.44556001 W
PLW13           0.22411001 W

F2 - Processing parameters
SI                   32768
SF             125.7577791 MHz
WDW                     EM
SSB                      0
LB                    1.00 Hz
GB                       0
PC                    1.40
```

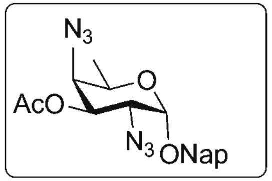

17

SSK-23-AP-DAT-ONAP-DEPT

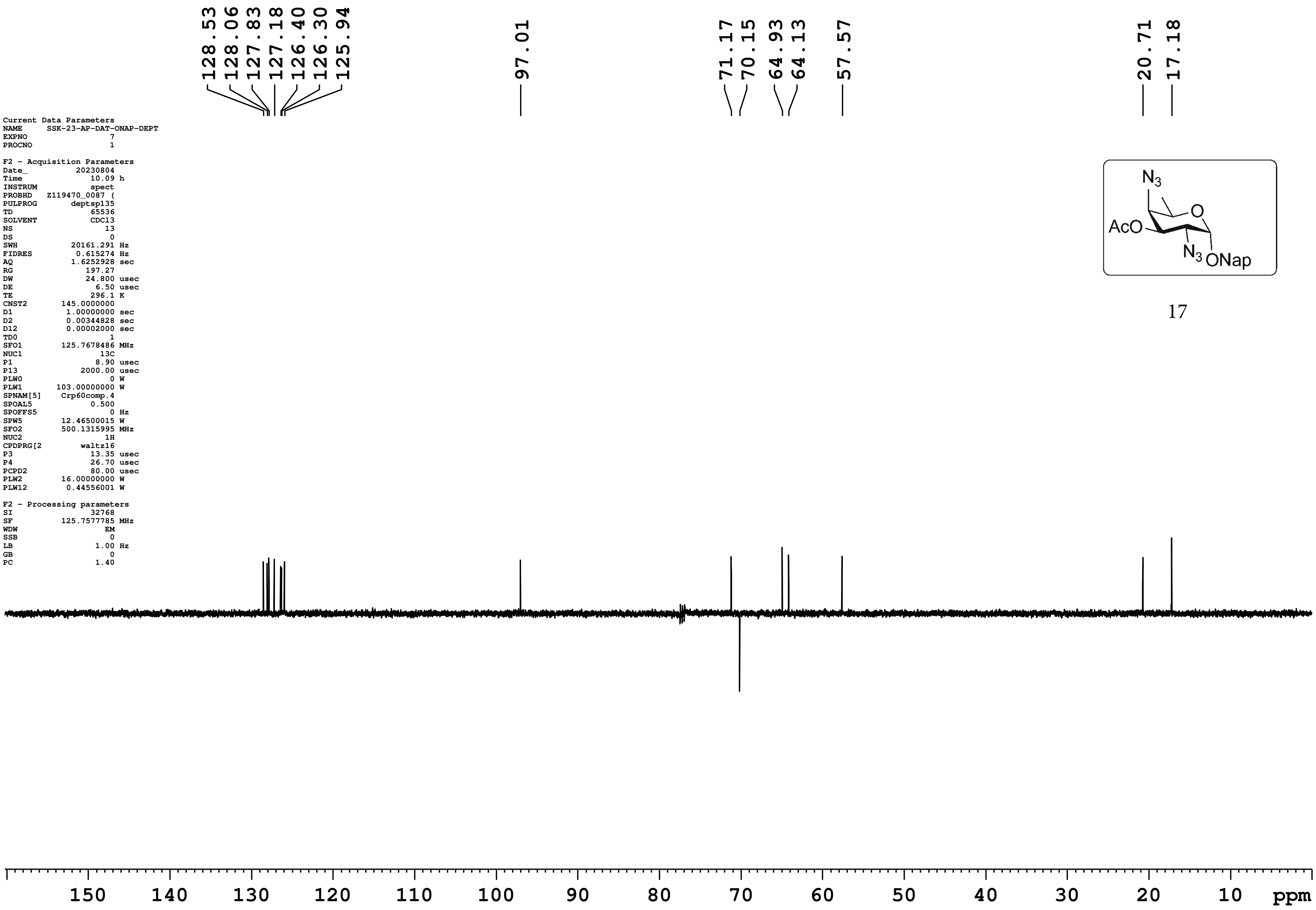


```
Current Data Parameters
NAME     SSK-23-AP-DAT-ONAP-DEPT
EXPNO                  7
PROCNO                 1

F2 - Acquisition Parameters
Date_          20230804
Time              10.09 h
INSTRUM           spect
PROBHD   Z119470_0087 (
PULPROG       deptsp135
TD                65536
SOLVENT           CDCl3
NS                   13
DS                    0
SWH           20161.291 Hz
FIDRES         0.615274 Hz
AQ            1.6252928 sec
RG               197.27
DW               24.800 usec
DE                 6.50 usec
TE                296.1 K
CNST2       145.0000000
D1           1.00000000 sec
D2           0.00344828 sec
D12          0.00002000 sec
TD0                   1
SFO1        125.7678486 MHz
NUC1                13C
P1                 8.90 usec
P13             2000.00 usec
PLW0                  0 W
PLW1       103.00000000 W
SPNAM[5]    Crp60comp.4
SPOAL5            0.500
SPOFFS5               0 Hz
SPW5        12.46500015 W
SFO2        500.1315995 MHz
NUC2                 1H
CPDPRG[2        waltz16
P3                13.35 usec
P4                26.70 usec
PCPD2             80.00 usec
PLW2        16.00000000 W
PLW12        0.44556001 W

F2 - Processing parameters
SI                32768
SF          125.7577785 MHz
WDW                  EM
SSB                   0
LB                 1.00 Hz
GB                    0
PC                 1.40
```


17

SSK-23-AP-DAT-ONAP-COSY

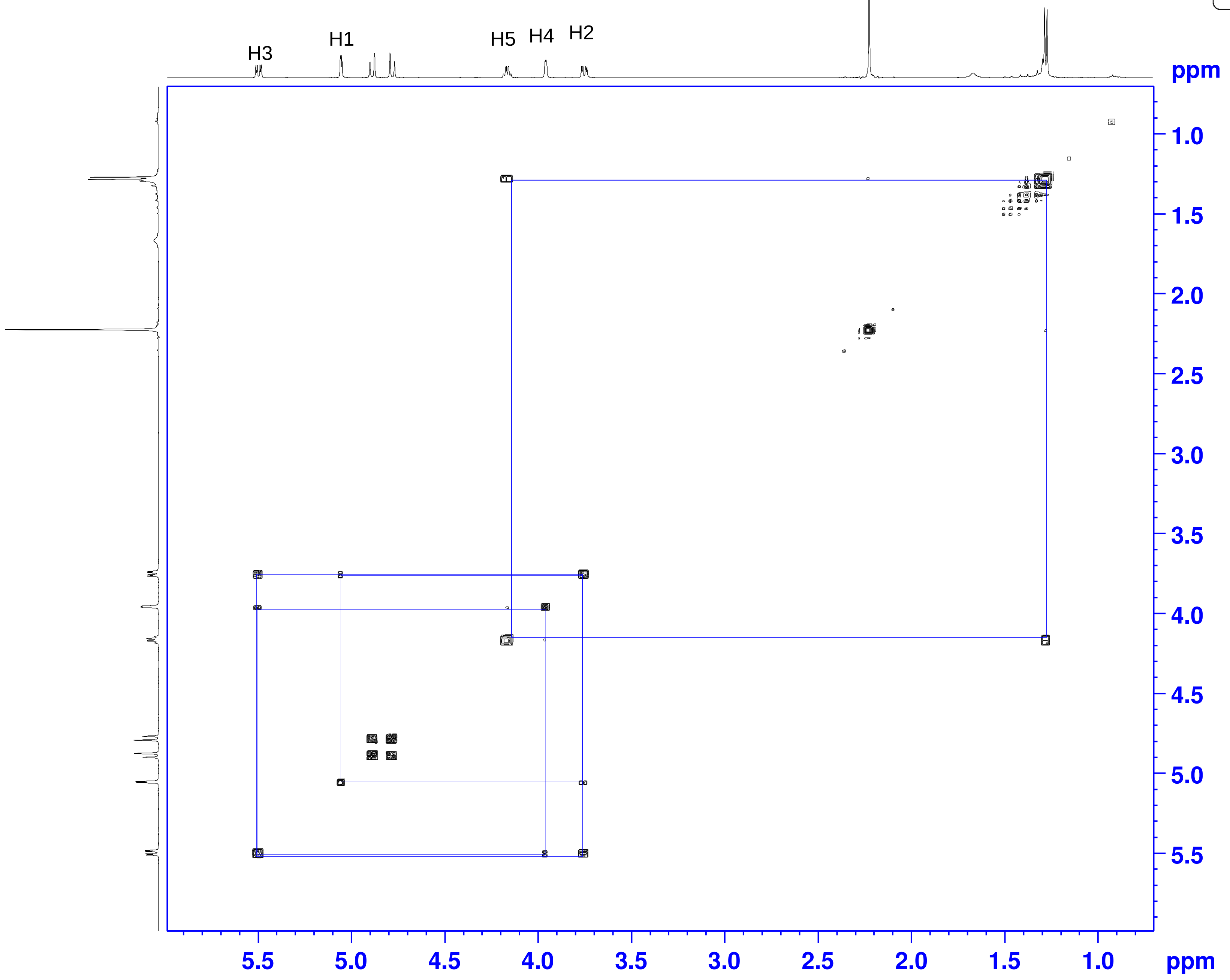

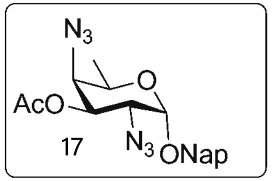

17

Current Data Parameters
NAME SSK-23-AP-DAT-ONAP-COSY
EXPNO 8
PROCNO 1

F2 - Acquisition Parameters
Date_ 20230804
Time 10.13 h
INSTRUM spect
PROBHD Z119470_0087 (
PULPROG cosygpppqf
TD 2048
SOLVENT CDCl3
NS 4
DS 0
SWH 2642.706 Hz
FIDRES 2.580768 Hz
AQ 0.3874816 sec
RG 61.42
DW 189.200 usec
DE 6.50 usec
TE 295.8 K
D0 0.00000300 sec
D1 1.00000000 sec
D11 0.03000000 sec
D12 0.00002000 sec
D13 0.00000400 sec
D16 0.00020000 sec
IN0 0.00037840 sec
TDav 1
SFO1 500.1316732 MHz
NUC1 1H
P0 13.35 usec
P1 13.35 usec
P17 2500.00 usec
PLW1 16.00000000 W
PLW10 3.16840005 W
GPNAM[1] SMSQ10.100
GPZ1 10.00 %
P16 1000.00 usec

F1 - Acquisition parameters
TD 38
SFO1 500.1317 MHz
FIDRES 139.089798 Hz
SW 5.284 ppm
FnMODE QF

F2 - Processing parameters
SI 1024
SF 500.1300000 MHz
WDW QSINE
SSB 0
LB 0 Hz
GB 0
PC 1.40

F1 - Processing parameters
SI 1024
MC2 QF
SF 500.1300000 MHz
WDW QSINE
SSB 0
LB 0 Hz
GB 0

SSK-23-AP-DAT-ONAP-HSQC

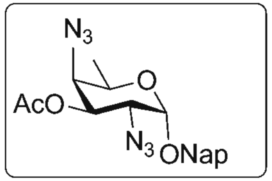

17

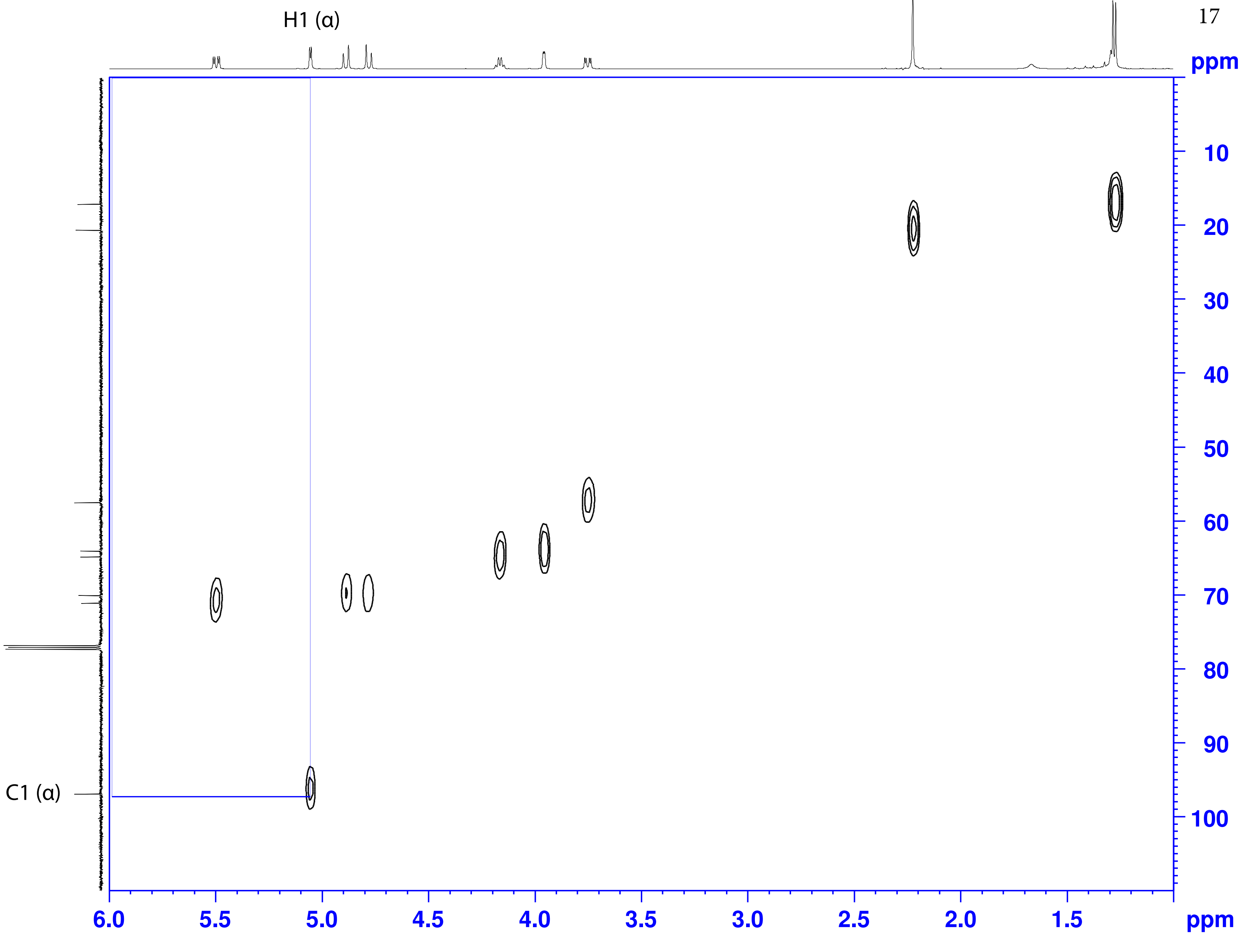


```
Current Data Parameters
NAME     SSK-23-AP-DAT-ONAP-HSQC
EXPNO                 9
PROCNO                1

F2 - Acquisition Parameters
Date_          20230804
Time              10.16 h
INSTRUM           spect
PROBHD   Z119470_0087 (
PULPROG  hsqcedetgpsisp2.2
TD                 1024
SOLVENT           CDCl3
NS                    4
DS                    0
SWH            8012.820 Hz
FIDRES        15.650040 Hz
AQ            0.0638976 sec
RG               197.27
DW               62.400 usec
DE                 6.50 usec
TE                295.7 K
CNST2       145.0000000
CNST17       -0.5000000
D0           0.00000300 sec
D1           1.00000000 sec
D4           0.00172414 sec
D11          0.03000000 sec
D16          0.00020000 sec
D21          0.00360000 sec
D24          0.00089000 sec
IN0          0.00002410 sec
TDav                  1
ZGOPTNS
SFO1        500.1323506 MHz
NUC1                 1H
P1                13.35 usec
P2                26.70 usec
PLW1        16.00000000 W
SFO2        125.7665916 MHz
NUC2                13C
CPDPRG[2 bi_p5m4sp_4sp.2
P3                 8.90 usec
P14              500.00 usec
P24             2000.00 usec
P63             1500.00 usec
PLW0                  0 W
PLW2       103.00000000 W
PLW12        1.66499996 W
SPNAM[3] Crp60,0.5,20.1
SPOAL3            0.500
SPOFFS3               0 Hz
SPW3        12.46500015 W
SPNAM[7]    Crp60comp.4
SPOAL7            0.500
SPOFFS7               0 Hz
SPW7        12.46500015 W
SPNAM[14 Crp32,1.5,20.2
SPOAL14           0.500
SPOFFS14              0 Hz
SPW14        5.31860018 W
SPNAM[31 Crp32,1.5,20.2
SPOAL31           0.500
SPOFFS31              0 Hz
SPW31        1.32969999 W
GPNAM[1]     SMSQ10.100
GPZ1              80.00 %
GPNAM[2]     SMSQ10.100
GPZ2              20.10 %
GPNAM[3]     SMSQ10.100
GPZ3              11.00 %
GPNAM[4]     SMSQ10.100
GPZ4              -5.00 %
P16             1000.00 usec
P19              600.00 usec

F1 - Acquisition parameters
TD                   30
SFO1           125.7666 MHz
FIDRES      1383.125854 Hz
SW              164.963 ppm
FnMODE     Echo-Antiecho

F2 - Processing parameters
SI                 1024
SF          500.1300000 MHz
WDW               QSINE
SSB                   2
LB                    0 Hz
GB                    0
PC                 1.40

F1 - Processing parameters
SI                 1024
MC2        echo-antiecho
SF          125.7577890 MHz
WDW               QSINE
SSB                   2
LB                    0 Hz
GB                    0
```

SSK-23-AP-DAT-ONAF-F-1H

Current Data Parameters
NAME SSK-23-AP-DAT-ONAF-F-1H
EXPNO 3
PROCNO 1

F2 - Acquisition Parameters
Date_ 20230830
Time 15.58 h
INSTRUM spect
PROBHD Z104450_0346 (
PULPROG zg30
TD 54274
SOLVENT CDCl3
NS 9
DS 0
SWH 8223.685 Hz
FIDRES 0.303043 Hz
AQ 3.2998593 sec
RG 90.5
DW 60.800 usec
DE 6.50 usec
TE 161.8 K
D1 1.00000000 sec
TD0 1
SFO1 400.1324710 MHz
NUC1 1H
P0 5.00 usec
P1 15.00 usec
PLW1 9.69999981 W

F2 - Processing parameters
SI 32768
SF 400.1300095 MHz
WDW EM
SSB 0
LB 0.30 Hz
GB 0
PC 1.00

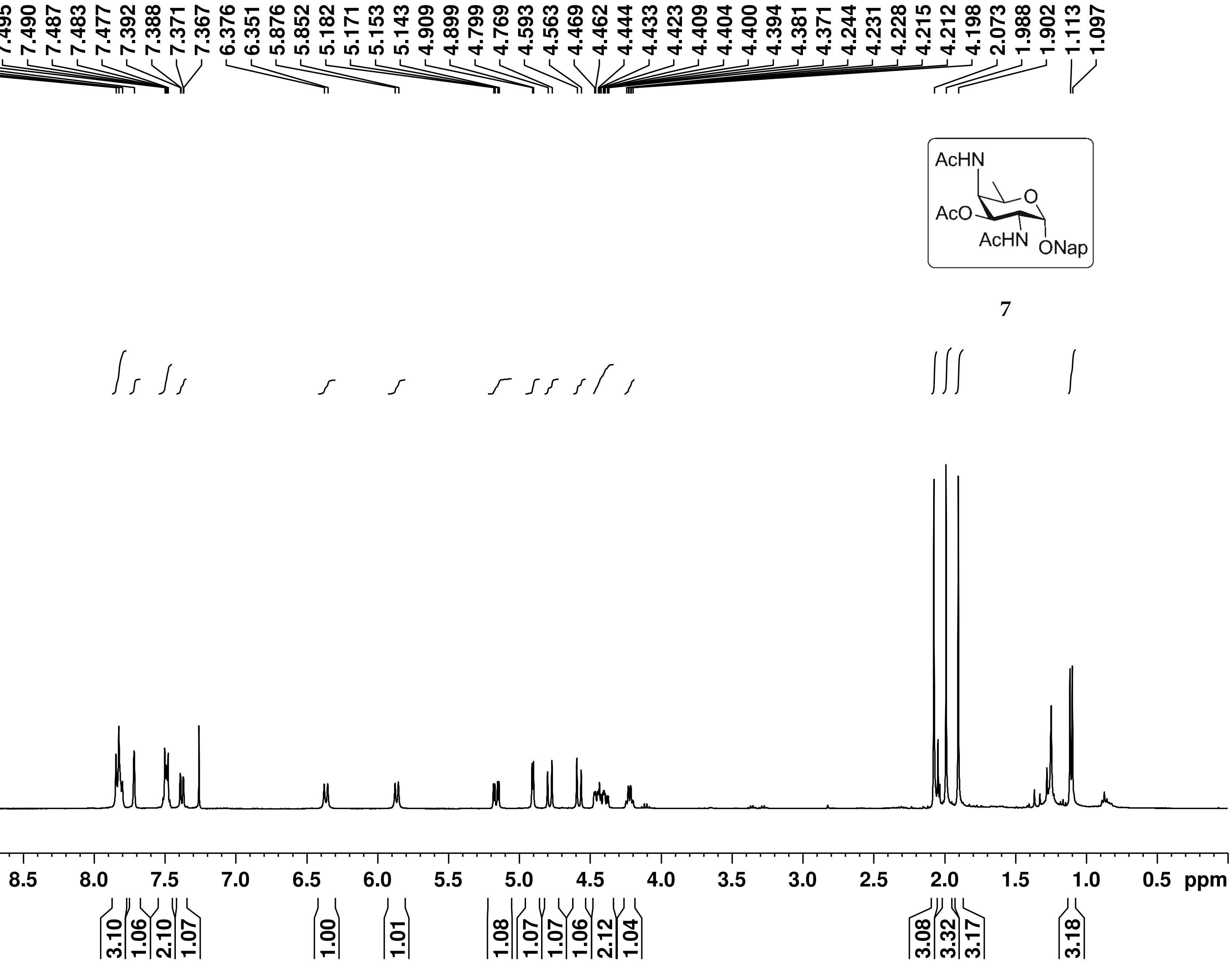

SSK-23-AP-DAT-ONAF-F-13C

Current Data Parameters
NAME SSK-23-AP-DAT-ONAF-F-13C
EXPNO 4
PROCNO 1

F2 - Acquisition Parameters
Date_ 20230830
Time 15.59 h
INSTRUM spect
PROBHD Z104450_0346 (
PULPROG zgpg30
TD 65536
SOLVENT CDCl3
NS 28
DS 0
SWH 26041.666 Hz
FIDRES 0.794729 Hz
AQ 1.2582912 sec
RG 1030
DW 19.200 usec
DE 6.50 usec
TE 161.7 K
D1 1.00000000 sec
D11 0.03000000 sec
TD0 1
SFO1 100.6238364 MHz
NUC1 13C
P0 3.33 usec
P1 10.00 usec
PLW1 47.00000000 W
SFO2 400.1316005 MHz
NUC2 1H
CPDPRG[2 waltz16
PCPD2 90.00 usec
PLW2 9.69999981 W
PLW12 0.26944000 W
PLW13 0.13552999 W

F2 - Processing parameters
SI 32768
SF 100.6127592 MHz
WDW EM
SSB 0
LB 1.00 Hz
GB 0
PC 1.40

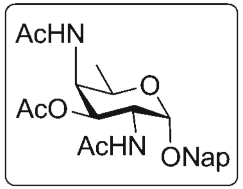

7

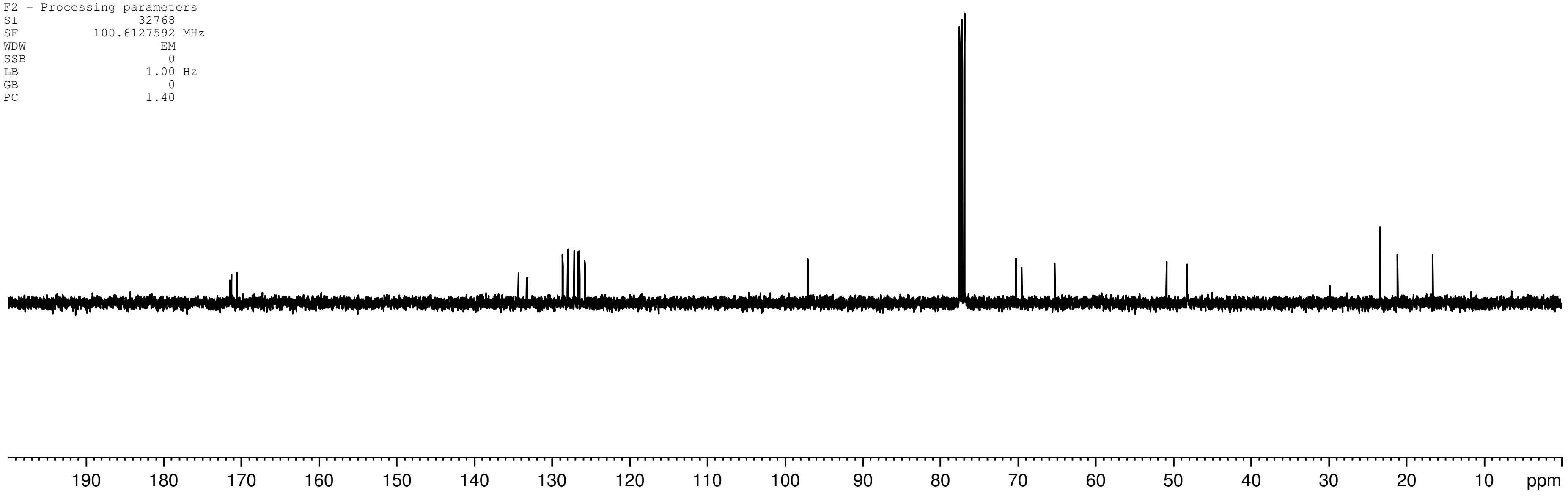

SSK-23-AP-DAT-ONAF-F-DEPT

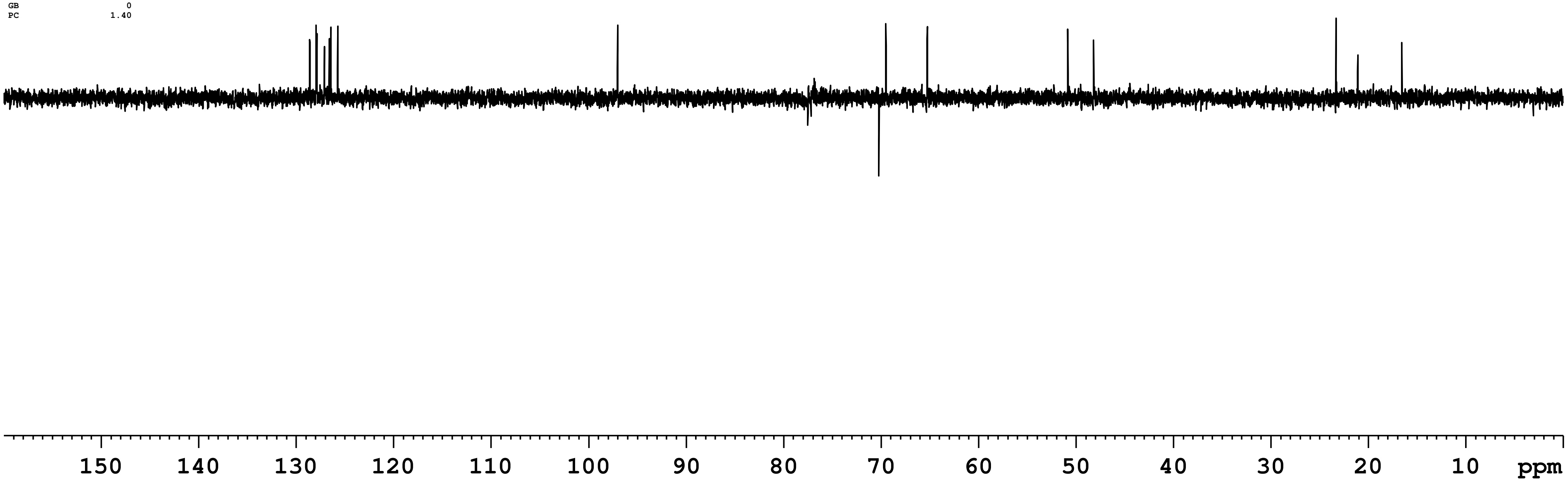

Current Data Parameters
NAME SSK-23-AP-DAT-ONAF-F-DEPT
EXPNO 5
PROCNO 1

F2 - Acquisition Parameters
Date_ 20230830
Time 16.00 h
INSTRUM spect
PROBHD Z104450_0346 (
PULPROG dept135
TD 65536
SOLVENT CDCl3
NS 15
DS 0
SWH 27777.777 Hz
FIDRES 0.847710 Hz
AQ 1.1796480 sec
RG 203
DW 18.000 usec
DE 6.50 usec
TE 161.7 K
CNST2 145.0000000
D1 1.00000000 sec
D2 0.00344828 sec
D12 0.00002000 sec
TD0 1
SFO1 100.6242389 MHz
NUC1 13C
P1 10.00 usec
P2 20.00 usec
PLW1 47.00000000 W
SFO2 400.1316005 MHz
NUC2 1H
CPDPRG[2 waltz16
P3 15.00 usec
P4 30.00 usec
PCPD2 90.00 usec
PLW2 9.69999981 W
PLW12 0.26944000 W

F2 - Processing parameters
SI 32768
SF 100.6127596 MHz
WDW EM
SSB 0
LB 1.00 Hz
GB 0
PC 1.40

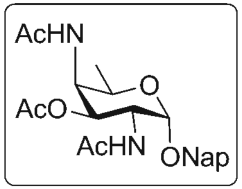

7

SSK-23-AP-DAT-ONAF-F-COSY

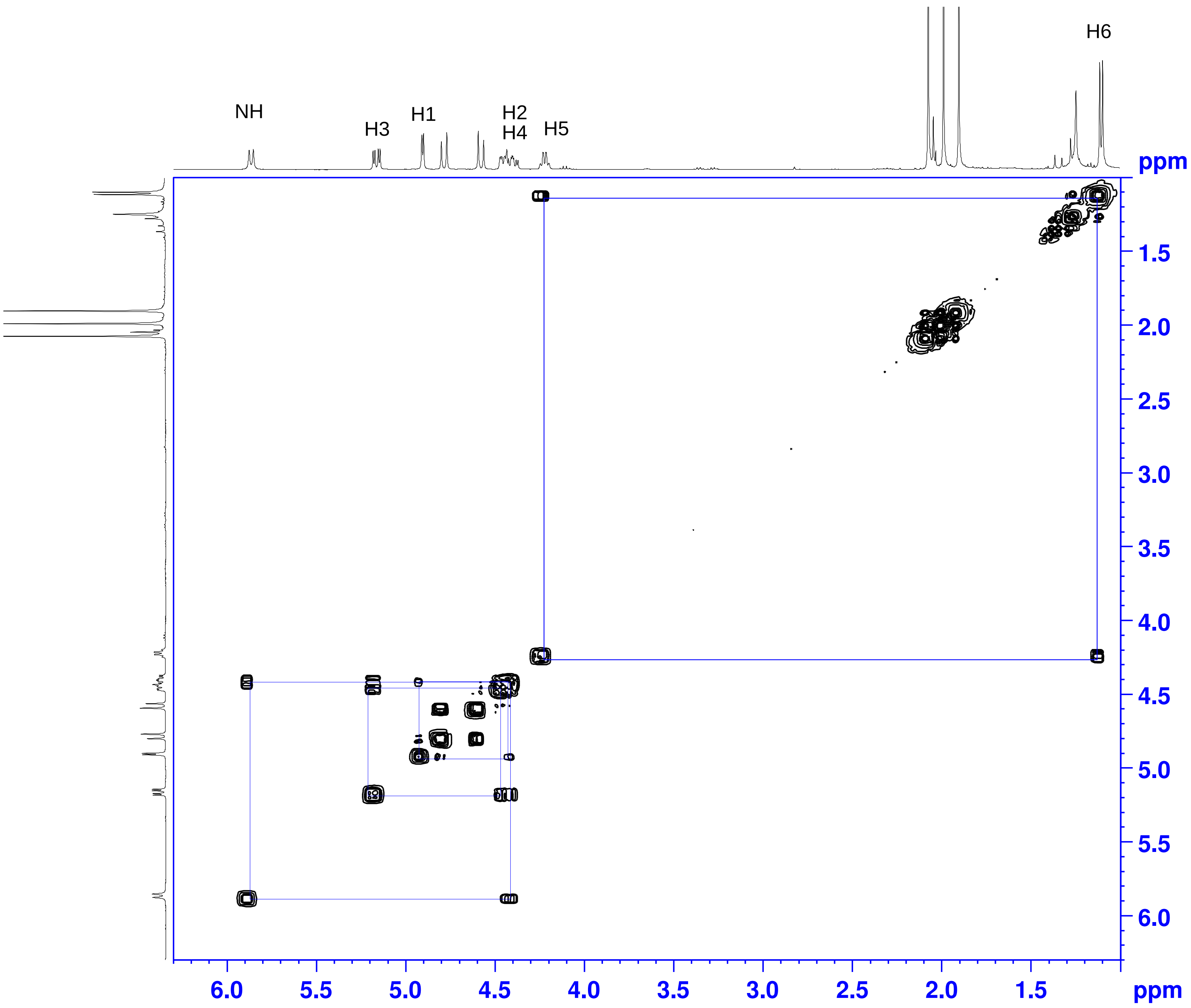

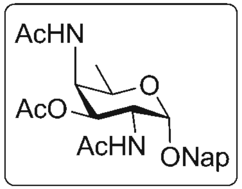

7

Current Data Parameters		
NAME	SSK-23-AP-DAT-ONAF-F-COSY	
EXPNO	6	
PROCNO	1	
F2 - Acquisition Parameters		
Date_	20230830	
Time	16.07	h
INSTRUM	spect	
PROBHD	Z104450_0346 (	
PULPROG	cosygpqf	
TD	2048	
SOLVENT	CDCl3	
NS	4	
DS	0	
SWH	5341.880	Hz
FIDRES	5.216680	Hz
AQ	0.1916928	sec
RG	64	
DW	93.600	usec
DE	6.50	usec
TE	161.6	K
D0	0.00000300	sec
D1	1.00000000	sec
D13	0.00000400	sec
D16	0.00020000	sec
IN0	0.00018720	sec
TDav	1	
SFO1	400.1324057	MHz
NUC1	1H	
P0	15.00	usec
P1	15.00	usec
PLW1	9.69999981	W
GPNAM[1]	SINE.100	
GPZ1	10.00	%
P16	1000.00	usec
F1 - Acquisition parameters		
TD	80	
SFO1	400.1324	MHz
FIDRES	133.547012	Hz
SW	13.350	ppm
FnMODE	QF	
F2 - Processing parameters		
SI	1024	
SF	400.1300000	MHz
WDW	SINE	
SSB	0	
LB	0	Hz
GB	0	
PC	1.40	
F1 - Processing parameters		
SI	1024	
MC2	QF	
SF	400.1300000	MHz
WDW	SINE	
SSB	0	
LB	0	Hz
GB	0	

SSK-23-AP-1262-1H

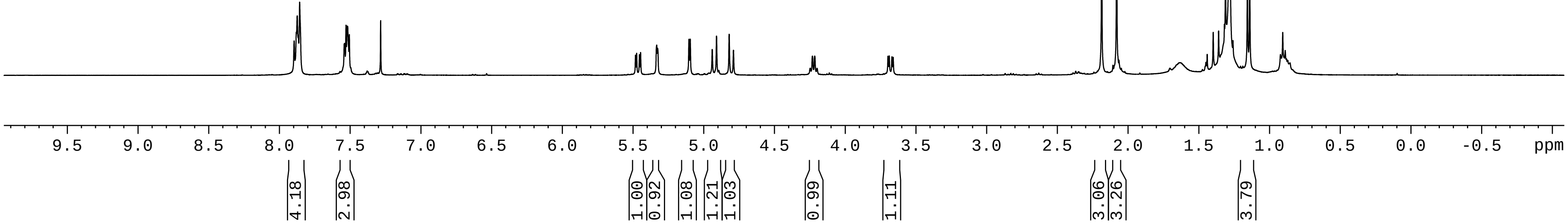


```
Current Data Parameters
NAME     SSK-23-AP-1262-1H
EXPNO                   10
PROCNO                   1

F2 - Acquisition Parameters
Date_           20230328
Time               13.13 h
INSTRUM  Avance Neo 400
PROBHD   Z163739_0226 (
PULPROG             zg30
TD                 51724
SOLVENT            CDCl3
NS                    18
DS                     0
SWH             8620.689 Hz
FIDRES          0.333334 Hz
AQ             2.9999919 sec
RG                   101
DW                58.000 usec
DE                 13.14 usec
TE                 297.2 K
D1            1.00000000 sec
TD0                    1
SFO1         400.1324708 MHz
NUC1                  1H
P0                  2.67 usec
P1                  8.00 usec
PLW1         25.07999992 W

F2 - Processing parameters
SI                 65536
SF           400.1300000 MHz
WDW                   EM
SSB                    0
LB                  0.30 Hz
GB                     0
PC                  1.00
```

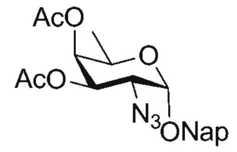

18

SSK-23-AP-1262-13C

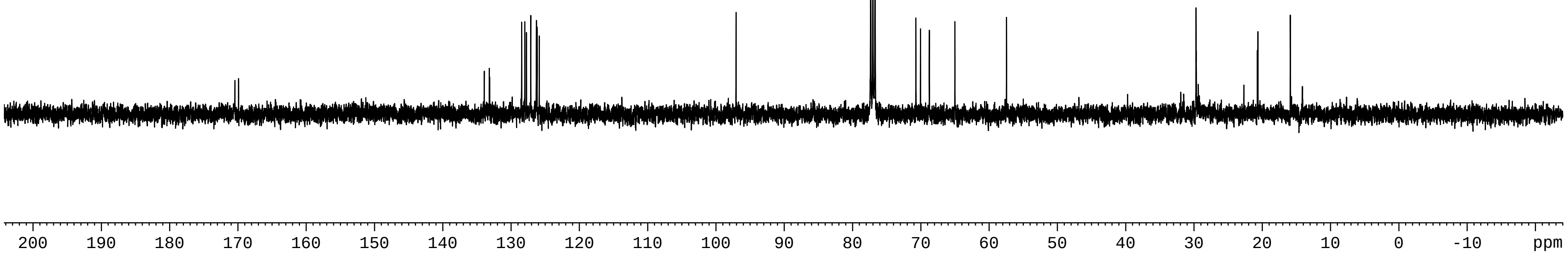


```
Current Data Parameters
NAME     SSK-23-AP-1262-13C
EXPNO                  12
PROCNO                  1

F2 - Acquisition Parameters
Date_            20230328
Time                13.19 h
INSTRUM  Avance Neo 400
PROBHD   Z163739_0226 (
PULPROG           zgpg30
TD                 65536
SOLVENT            CDCl3
NS                   143
DS                     2
SWH            27777.777 Hz
FIDRES          0.847710 Hz
AQ             1.1796480 sec
RG                   101
DW                18.000 usec
DE                  6.50 usec
TE                 297.9 K
D1            1.00000000 sec
D11           0.03000000 sec
TD0                    1
SFO1         100.6242384 MHz
NUC1                 13C
P0                  2.67 usec
P1                  8.00 usec
PLW1         99.33999634 W
SFO2         400.1316005 MHz
NUC2                  1H
CPDPRG[2         waltz65
PCPD2              90.00 usec
PLW2         25.07999992 W
PLW12         0.19815999 W
PLW13         0.09967500 W

F2 - Processing parameters
SI                 32768
SF           100.6127685 MHz
WDW                   EM
SSB                    0
LB                  1.00 Hz
GB                     0
PC                  1.40
```

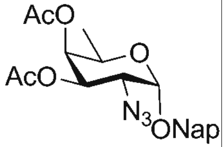

18

SSK-23-AP-1262-DEPT

```
Current Data Parameters
NAME     SSK-23-AP-1262-DEPT
EXPNO                   14
PROCNO                   1

F2 - Acquisition Parameters
Date_             20230328
Time                 13.23 h
INSTRUM  Avance Neo 400
PROBHD   Z163739_0226 (
PULPROG          deptsp135
TD                   65536
SOLVENT              CDCl3
NS                      76
DS                       4
SWH              27777.777 Hz
FIDRES            0.847710 Hz
AQ               1.1796480 sec
RG                     101
DW                  18.000 usec
DE                    6.50 usec
TE                   297.7 K
CNST2        145.0000000
D1            1.00000000 sec
D2            0.00344828 sec
D12           0.00002000 sec
TD0                      1
SFO1         100.6242384 MHz
NUC1                   13C
P1                    8.00 usec
P13                2000.00 usec
PLW0                     0 W
PLW1         99.33999634 W
SPNAM[5]     Crp60comp.4
SPOAL5               0.500
SPOFFS5                  0 Hz
SPW5          9.71399975 W
SFO2         400.1316005 MHz
NUC2                    1H
CPDPRG[2           waltz65
P3                    8.00 usec
P4                   16.00 usec
PCPD2                90.00 usec
PLW2         25.07999992 W
PLW12         0.19815999 W

F2 - Processing parameters
SI                   32768
SF           100.6127685 MHz
WDW                     EM
SSB                      0
LB                    1.00 Hz
GB                       0
PC                    1.40
```

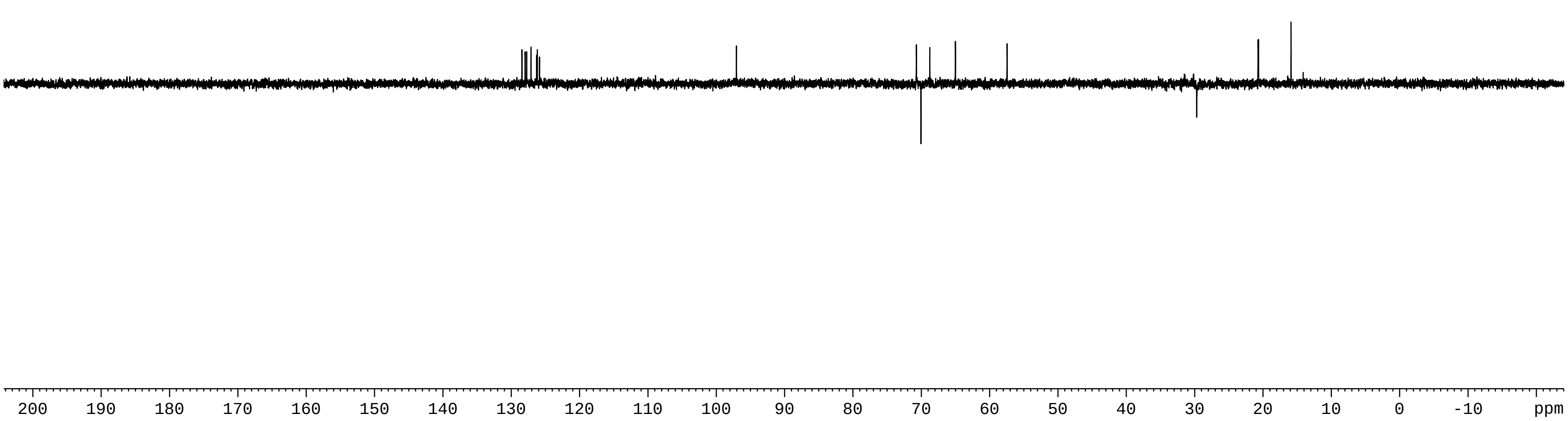

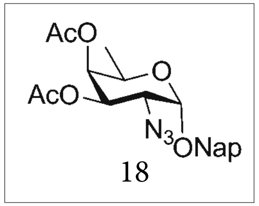

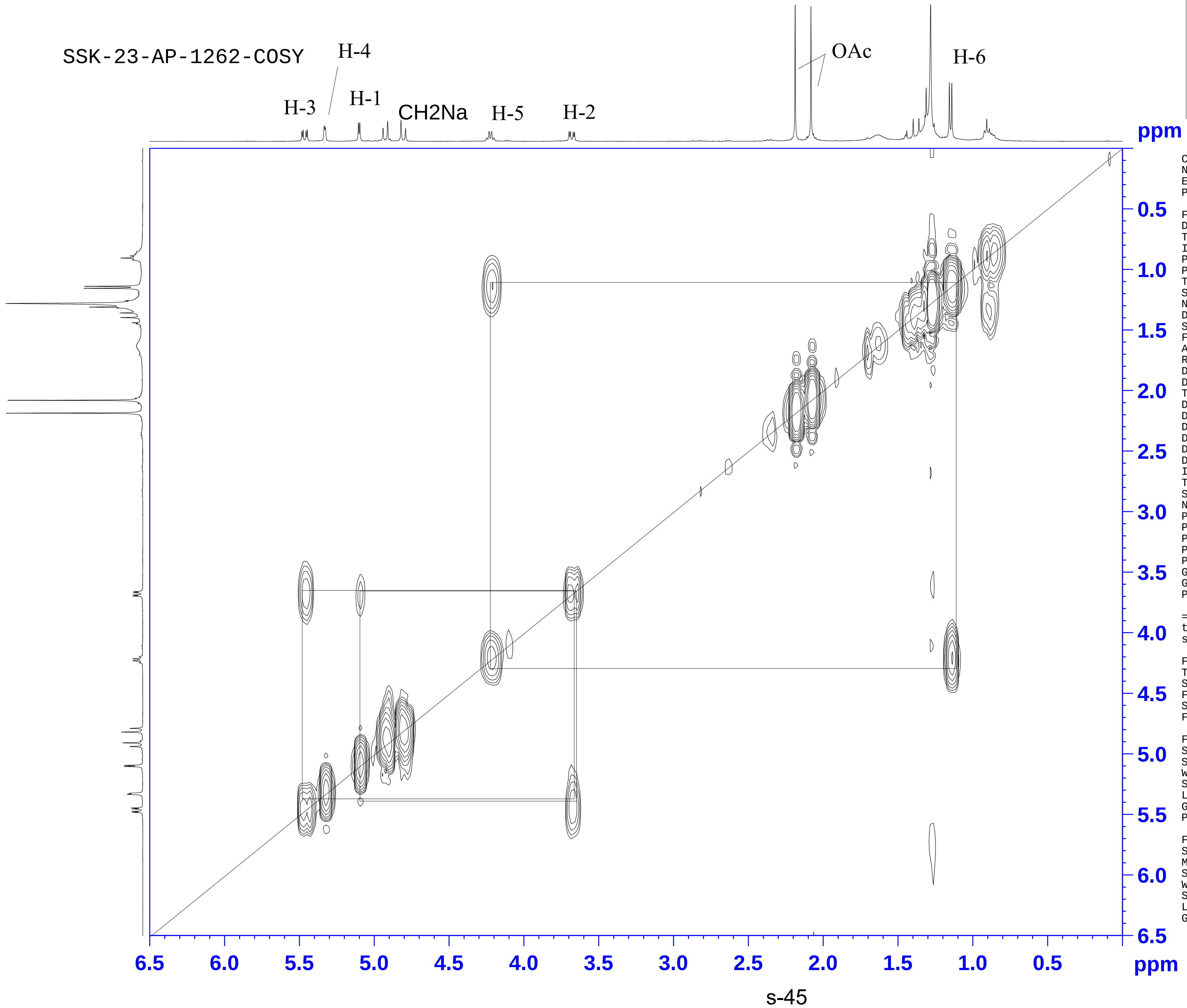

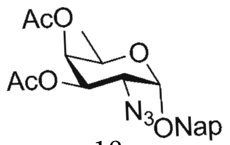

18

```
Current Data Parameters
NAME     SSK-23-AP-1262-COSY
EXPNO                     16
PROCNO                     1

F2 - Acquisition Parameters
Date_               20230328
Time                 13.31 h
INSTRUM       Avance Neo 400
PROBHD       Z163739_0226 (
PULPROG           cosygpppqf
TD                      2048
SOLVENT                CDCl3
NS                         4
DS                         0
SWH              8620.689 Hz
FIDRES           8.418642 Hz
AQ             0.1187840 sec
RG                       101
DW               58.000 usec
DE                 6.50 usec
TE                   297.4 K
D0            0.00000300 sec
D1            1.00000000 sec
D11           0.03000000 sec
D12           0.00002000 sec
D13           0.00000400 sec
D16           0.00020000 sec
IN0           0.00011360 sec
TDav                       1
SFO1         400.1324708 MHz
NUC1                      1H
P0                 8.00 usec
P1                 8.00 usec
P17             2500.00 usec
PLW1          25.07999992 W
PLW10          1.78349996 W
GPNAM[1]         SMSQ10.100
GPZ1                 10.00 %
P16             1000.00 usec

======== F1 INDIRECT DIMENSION ========
td1       128
sw_F1     21.999996

F1 - Acquisition parameters
TD                        91
SFO1           400.1325 MHz
FIDRES       193.468506 Hz
SW                22.000 ppm
FnMODE                    QF

F2 - Processing parameters
SI                      1024
SF           400.1300000 MHz
WDW                    QSINE
SSB                        0
LB                      0 Hz
GB                         0
PC                      1.40

F1 - Processing parameters
SI                      1024
MC2                       QF
SF           400.1300000 MHz
WDW                    QSINE
SSB                        0
LB                      0 Hz
GB                         0
```

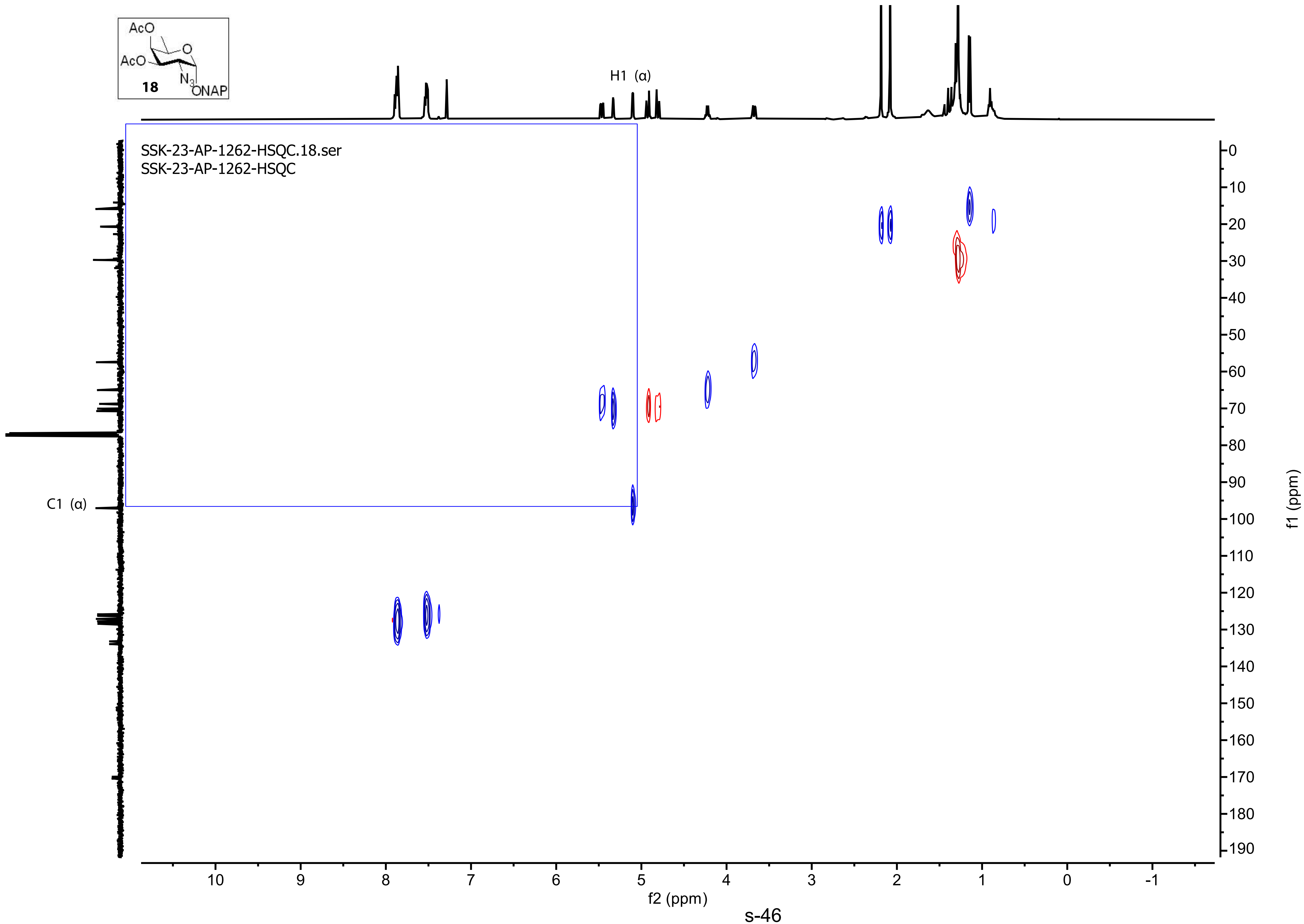
AcO
AcO
O
N3
ONAP
18
H1 (α)
C1 (α)
SSK-23-AP-1262-HSQC.18.ser
SSK-23-AP-1262-HSQC
f2 (ppm)
f1 (ppm)

SSK-23-AP-1264-1H

Current Data Parameters
NAME SSK-23-AP-1264-1H
EXPNO 1
PROCNO 1

F2 - Acquisition Parameters
Date_ 20230401
Time 10.54 h
INSTRUM Avance
PROBHD Z163739_0237 (
PULPROG zg30
TD 51724
SOLVENT CDCl3
NS 18
DS 0
SWH 8620.689 Hz
FIDRES 0.333334 Hz
AQ 2.9999919 sec
RG 95.1443
DW 58.000 usec
DE 13.14 usec
TE 297.2 K
D1 1.00000000 sec
TD0 1
SFO1 400.3024719 MHz
NUC1 1H
P0 2.67 usec
P1 8.00 usec
PLW1 21.00099945 W

F2 - Processing parameters
SI 65536
SF 400.3000000 MHz
WDW EM
SSB 0
LB 0.30 Hz
GB 0
PC 1.00

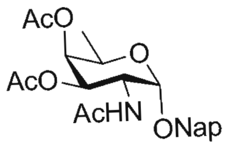

18

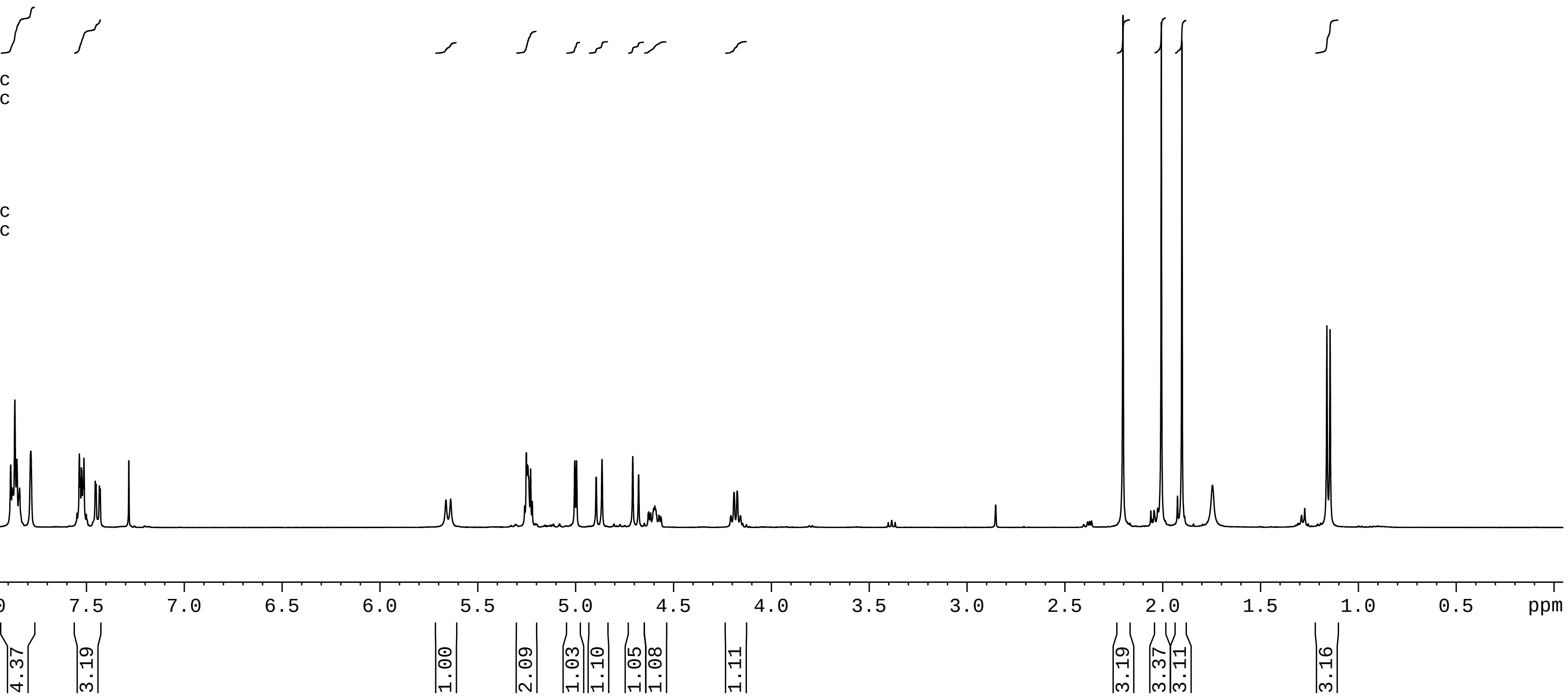

SSK-23-AP-1264-13C

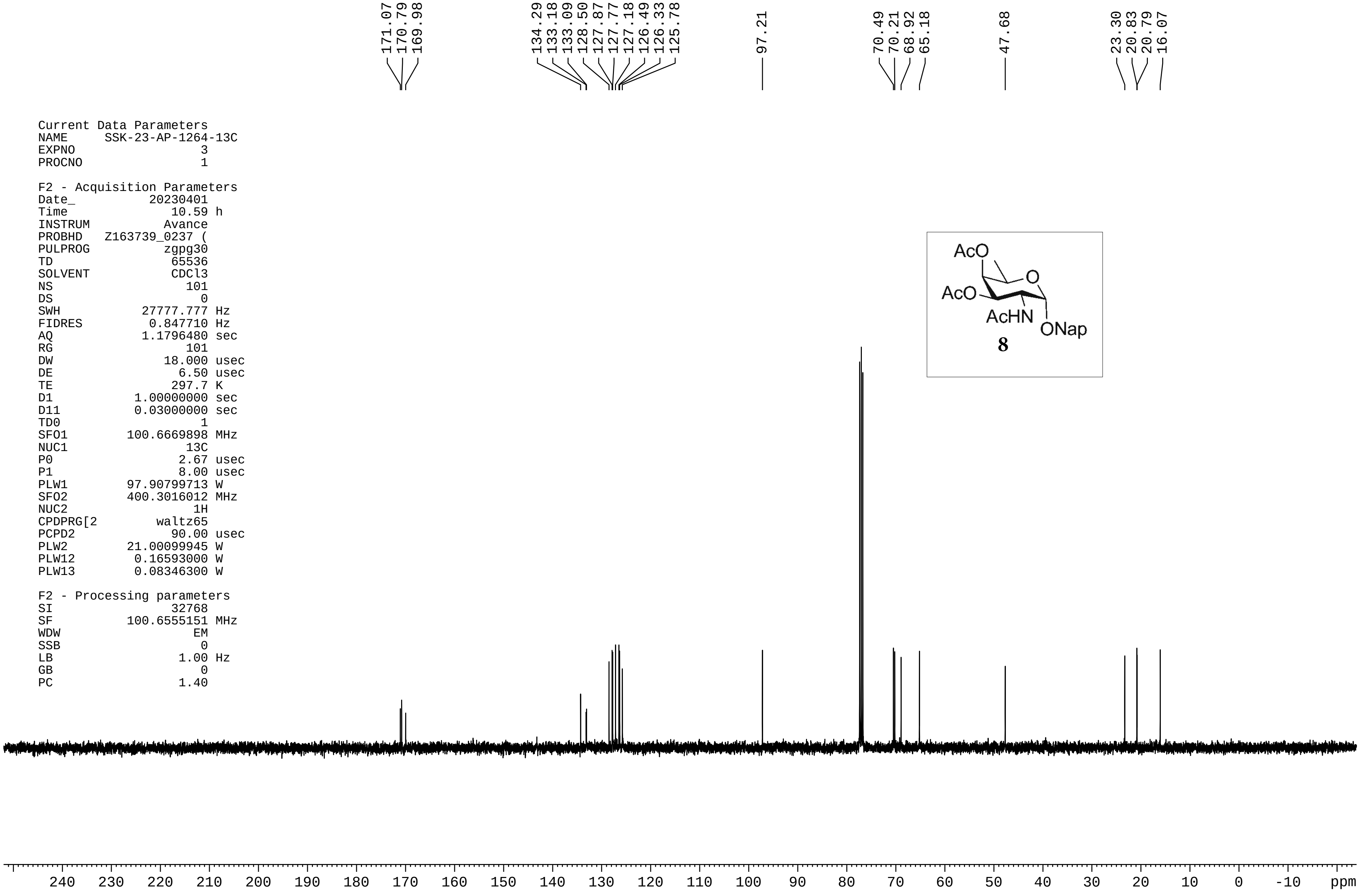

8

Current Data Parameters
NAME SSK-23-AP-1264-13C
EXPNO 3
PROCNO 1

F2 - Acquisition Parameters
Date_ 20230401
Time 10.59 h
INSTRUM Avance
PROBHD Z163739_0237 (
PULPROG zgpg30
TD 65536
SOLVENT CDCl3
NS 101
DS 0
SWH 27777.777 Hz
FIDRES 0.847710 Hz
AQ 1.1796480 sec
RG 101
DW 18.000 usec
DE 6.50 usec
TE 297.7 K
D1 1.00000000 sec
D11 0.03000000 sec
TD0 1
SFO1 100.6669898 MHz
NUC1 13C
P0 2.67 usec
P1 8.00 usec
PLW1 97.90799713 W
SFO2 400.3016012 MHz
NUC2 1H
CPDPRG[2 waltz65
PCPD2 90.00 usec
PLW2 21.00099945 W
PLW12 0.16593000 W
PLW13 0.08346300 W

F2 - Processing parameters
SI 32768
SF 100.6555151 MHz
WDW EM
SSB 0
LB 1.00 Hz
GB 0
PC 1.40

SSK-23-AP-1264-DEPT

```
Current Data Parameters
NAME     SSK-23-AP-1264-DEPT
EXPNO                  5
PROCNO                 1

F2 - Acquisition Parameters
Date_           20230401
Time               11.01 h
INSTRUM           Avance
PROBHD   Z163739_0237 (
PULPROG       deptsp135
TD                 65536
SOLVENT            CDCl3
NS                    22
DS                     0
SWH            27777.777 Hz
FIDRES          0.847710 Hz
AQ             1.1796480 sec
RG                   101
DW                18.000 usec
DE                  6.50 usec
TE                 297.6 K
CNST2        145.0000000
D1            1.00000000 sec
D2            0.00344828 sec
D12           0.00002000 sec
TD0                    1
SFO1         100.6669898 MHz
NUC1                 13C
P1                  8.00 usec
P13              2000.00 usec
PLW0                   0 W
PLW1         97.90799713 W
SPNAM[5]    Crp60comp.4
SPOAL5             0.500
SPOFFS5                0 Hz
SPW5          9.57390022 W
SFO2         400.3016012 MHz
NUC2                  1H
CPDPRG[2         waltz65
P3                  8.00 usec
P4                 16.00 usec
PCPD2              90.00 usec
PLW2         21.00099945 W
PLW12         0.16593000 W

F2 - Processing parameters
SI                 32768
SF           100.6555151 MHz
WDW                   EM
SSB                    0
LB                  1.00 Hz
GB                     0
PC                  1.40
```

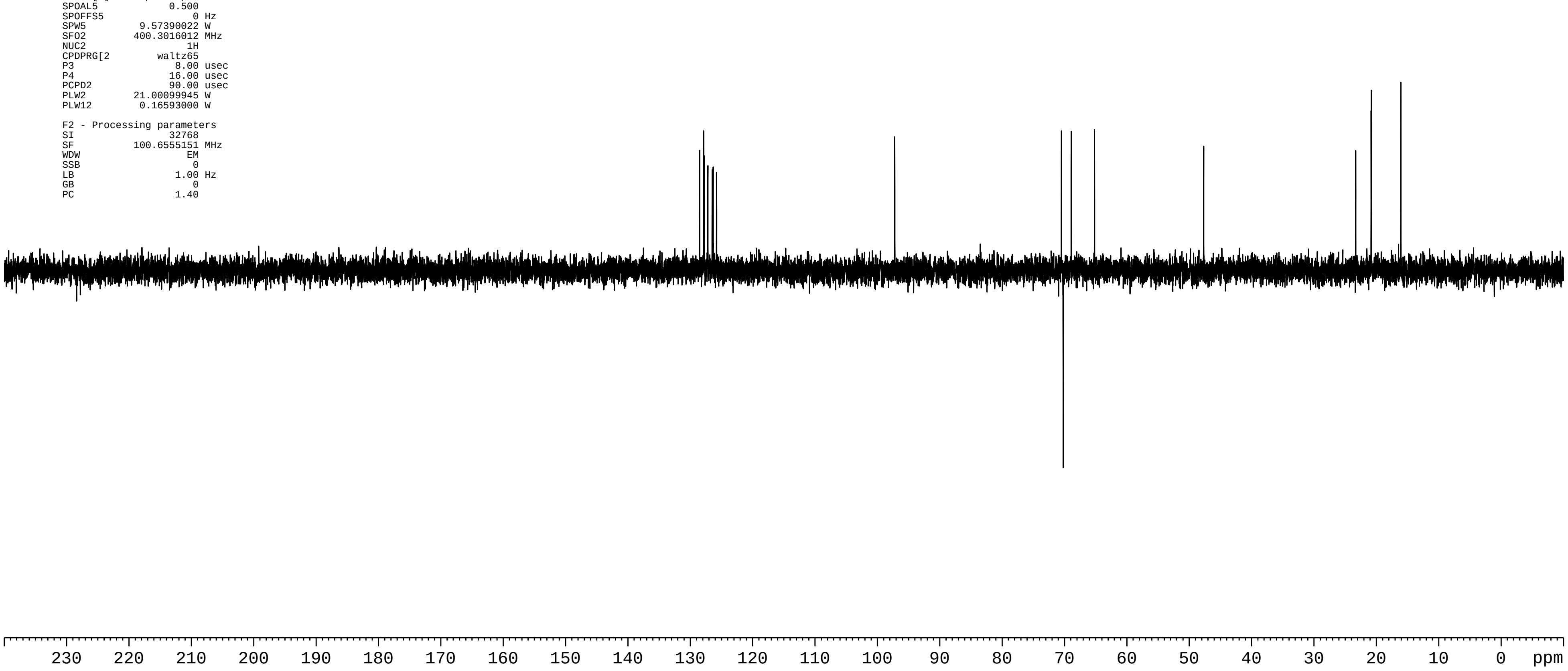

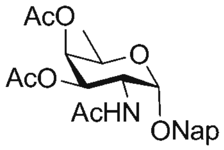

8

SSK-23-AP-1264-COSY

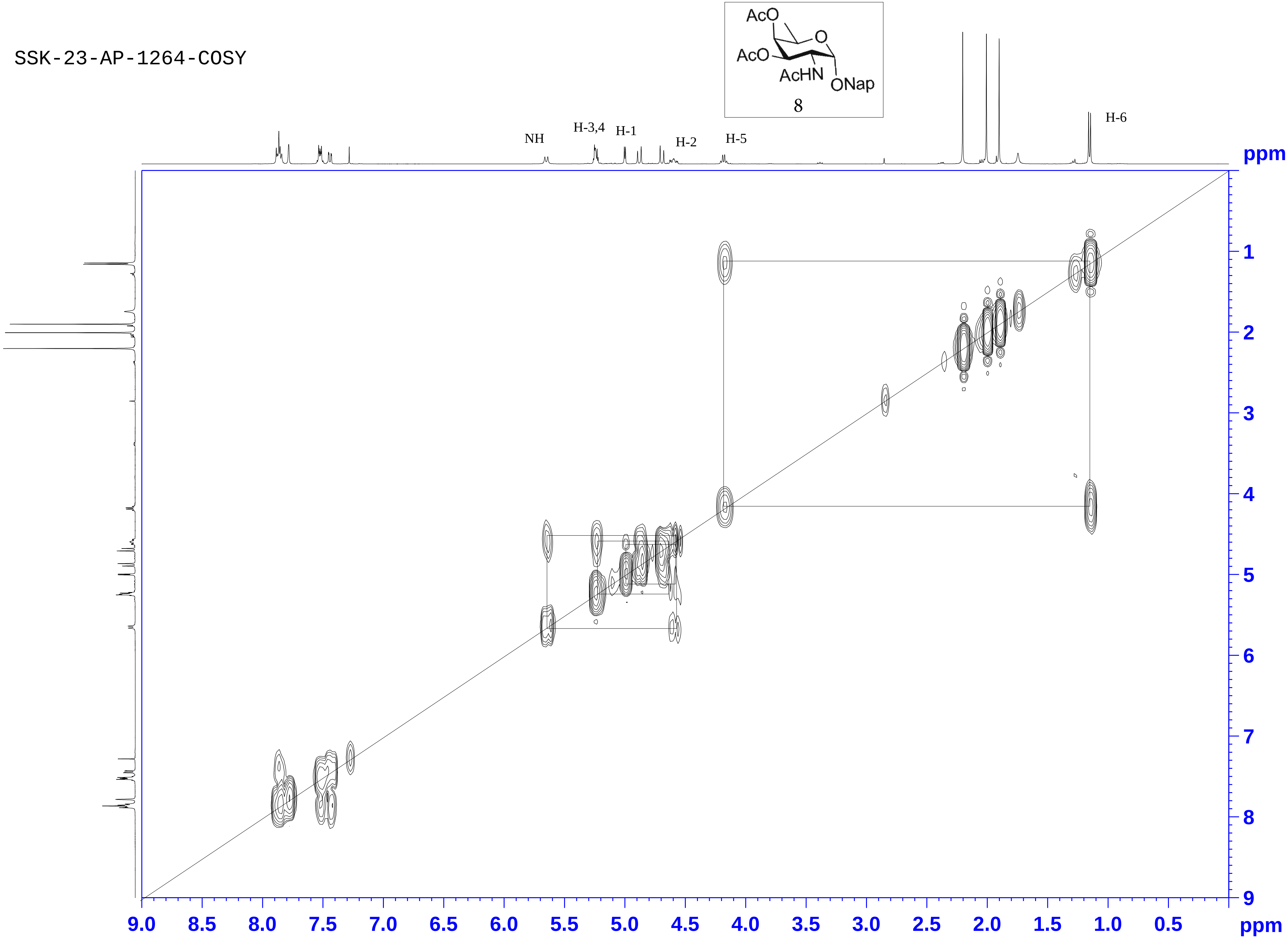

SSK-32-SKM-BAC-ANTH-1H

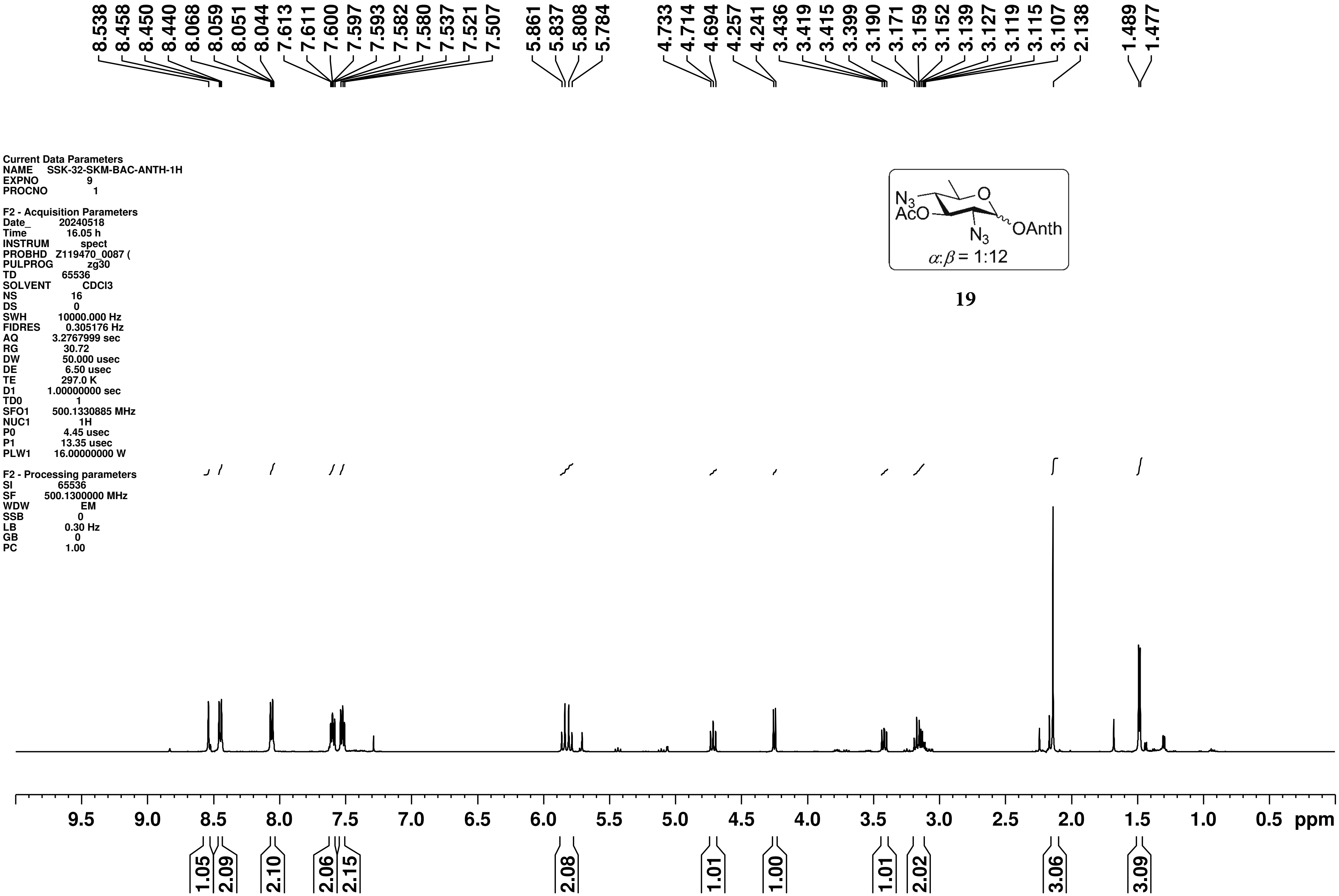

SSK-32-SKM-BAC-ANTH-13C

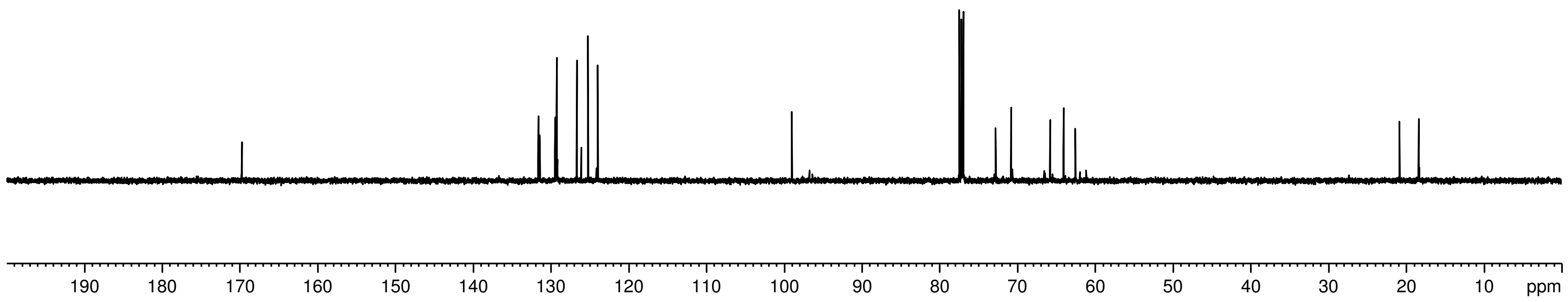


```
Current Data Parameters
NAME      SSK-32-SKM-BAC-ANTH-13C
EXPNO                    10
PROCNO                    1

F2 - Acquisition Parameters
Date_           20240518
Time               16.06 h
INSTRUM            spect
PROBHD   Z119470_0087 (
PULPROG          zgpg30
TD                65536
SOLVENT           CDCl3
NS                   20
DS                    0
SWH           34722.223 Hz
FIDRES         1.059638 Hz
AQ            0.9437184 sec
RG               197.27
DW               14.400 usec
DE                 6.50 usec
TE                297.2 K
D1           1.00000000 sec
D11          0.03000000 sec
TD0                   1
SFO1        125.7721254 MHz
NUC1                13C
P0                 2.97 usec
P1                 8.90 usec
PLW1       103.00000000 W
SFO2        500.1320005 MHz
NUC2                 1H
CPDPRG[2        waltz16
PCPD2             80.00 usec
PLW2        16.00000000 W
PLW12        0.44556001 W
PLW13        0.22411001 W

F2 - Processing parameters
SI                32768
SF          125.7577842 MHz
WDW                  EM
SSB                   0
LB                 1.00 Hz
GB                    0
PC                 1.40
```

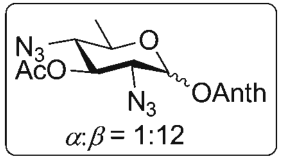

19

SSK-32-SKM-BAC-ANTH-DEPT

```
Current Data Parameters
NAME      SSK-32-SKM-BAC-ANTH-DEPT
EXPNO               11
PROCNO               1

F2 - Acquisition Parameters
Date_          20240518
Time              16.08 h
INSTRUM           spect
PROBHD   Z119470_0087 (
PULPROG       deptsp135
TD                65536
SOLVENT           CDCl3
NS                   28
DS                    0
SWH           20161.291 Hz
FIDRES         0.615274 Hz
AQ            1.6252928 sec
RG               197.27
DW               24.800 usec
DE                 6.50 usec
TE                297.4 K
CNST2       145.0000000
D1           1.00000000 sec
D2           0.00344828 sec
D12          0.00002000 sec
TD0                   1
SFO1        125.7678486 MHz
NUC1                13C
P1                 8.90 usec
P13             2000.00 usec
PLW0                  0 W
PLW1       103.00000000 W
SPNAM[5]    Crp60comp.4
SPOAL5            0.500
SPOFFS5               0 Hz
SPW5        12.46500015 W
SFO2        500.1315995 MHz
NUC2                 1H
CPDPRG[2        waltz16
P3                13.35 usec
P4                26.70 usec
PCPD2             80.00 usec
PLW2        16.00000000 W
PLW12        0.44556001 W

F2 - Processing parameters
SI                32768
SF          125.7577854 MHz
WDW                  EM
SSB                   0
LB                 1.00 Hz
GB                    0
PC                 1.40
```

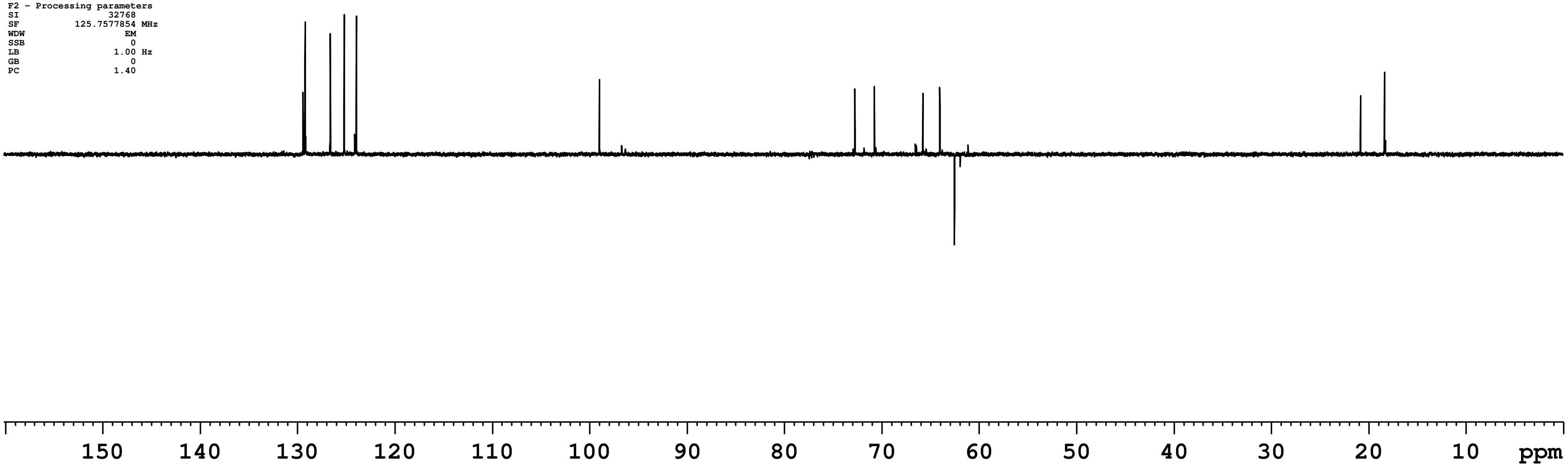

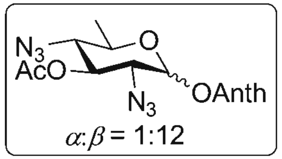

19

SSK-32-SKM-BAC-ANTH-COSY

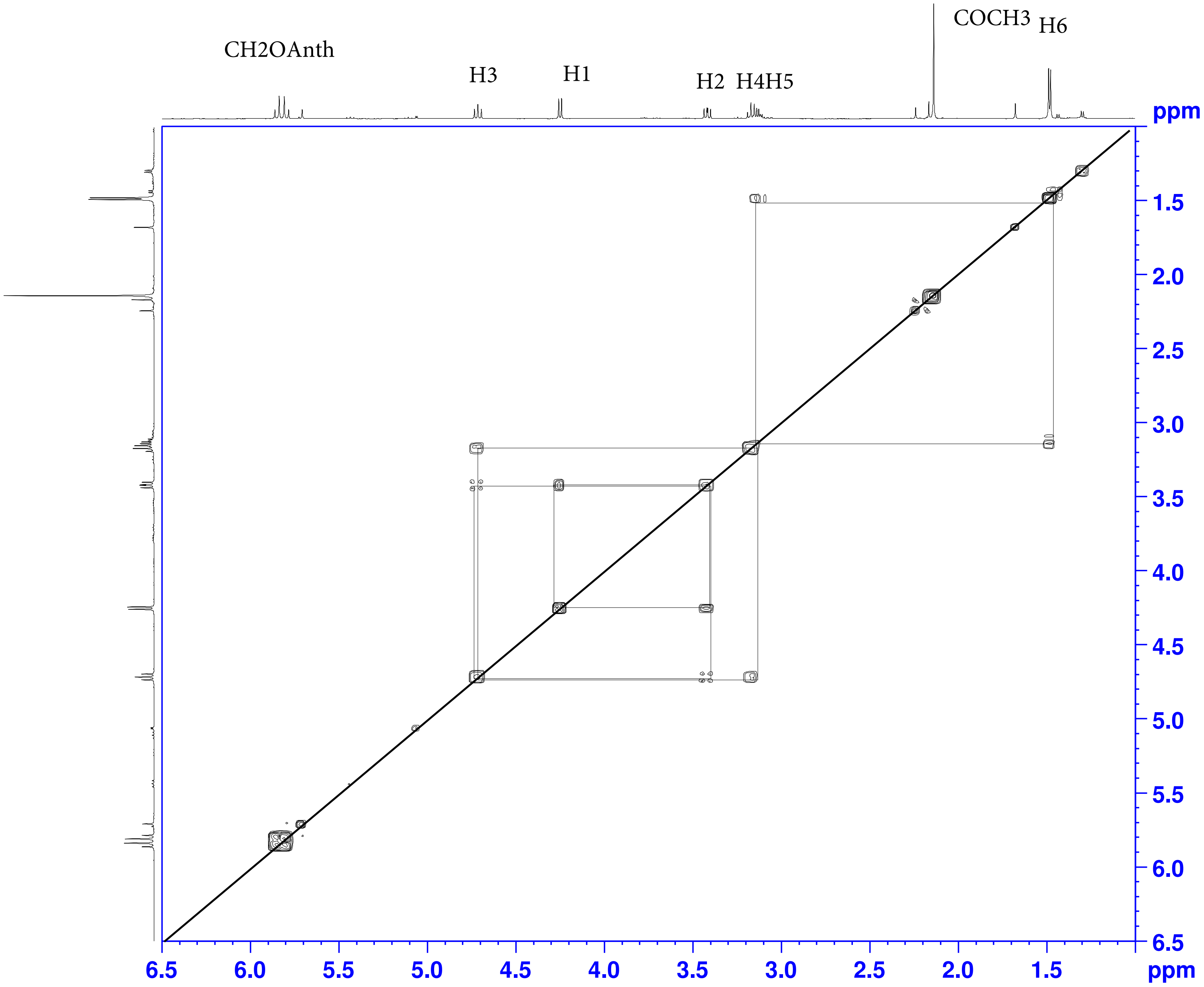

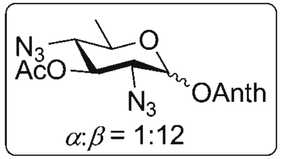

19

```
Current Data Parameters
NAME       SSK-32-SKM-BAC-ANTH-COSY
EXPNO                    11
PROCNO                    1

F2 - Acquisition Parameters
Date_              20240518
Time                  16.20 h
INSTRUM               spect
PROBHD   Z119470_0087 (
PULPROG          cosygpppqf
TD                     2048
SOLVENT               CDCl3
NS                        4
DS                        0
SWH                6684.492 Hz
FIDRES             6.527824 Hz
AQ                0.1531904 sec
RG                    61.42
DW                   74.800 usec
DE                     6.50 usec
TE                    297.2 K
D0               0.00000300 sec
D1               1.00000000 sec
D11              0.03000000 sec
D12              0.00002000 sec
D13              0.00000400 sec
D16              0.00020000 sec
IN0              0.00014960 sec
TDav                      1
SFO1            500.1330069 MHz
NUC1                     1H
P0                    13.35 usec
P1                    13.35 usec
P17                 2500.00 usec
PLW1            16.00000000 W
PLW10            3.16840005 W
GPNAM[1]        SMSQ10.100
GPZ1                  10.00 %
P16                 1000.00 usec

F1 - Acquisition parameters
TD                      128
SFO1                500.133 MHz
FIDRES           104.445190 Hz
SW                   13.365 ppm
FnMODE                   QF

F2 - Processing parameters
SI                     1024
SF              500.1300000 MHz
WDW                   QSINE
SSB                       0
LB                        0 Hz
GB                        0
PC                     1.40

F1 - Processing parameters
SI                     1024
MC2                      QF
SF              500.1300000 MHz
WDW                   QSINE
SSB                       0
LB                        0 Hz
GB                        0
```

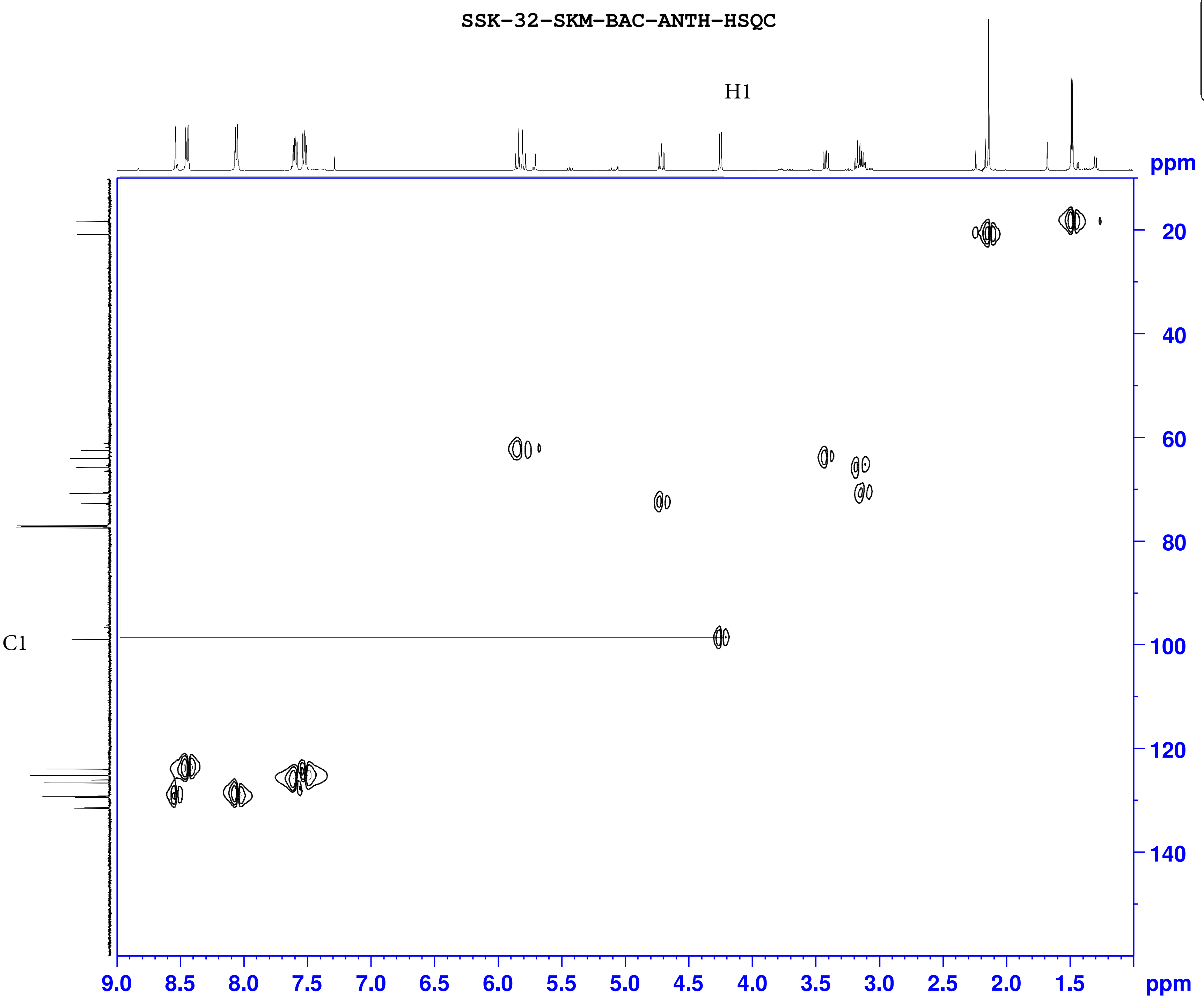

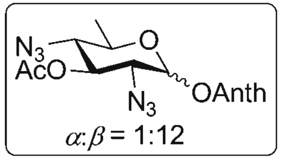

19

```
Current Data Parameters
NAME     SSK-32-SKM-BAC-ANTH-HSQC
EXPNO                     12
PROCNO                     1

F2 - Acquisition Parameters
Date_              20240518
Time                  16.09 h
INSTRUM               spect
PROBHD    Z119470_0087 (
PULPROG  hsqcedetgpsisp2.2
TD                     1024
SOLVENT               CDCl3
NS                        2
DS                        0
SWH                8012.820 Hz
FIDRES            15.650040 Hz
AQ                0.0638976 sec
RG                   197.27
DW                   62.400 usec
DE                     6.50 usec
TE                    297.3 K
CNST2           145.0000000
CNST17           -0.5000000
D0               0.00000300 sec
D1               1.00000000 sec
D4               0.00172414 sec
D11              0.03000000 sec
D16              0.00020000 sec
D21              0.00360000 sec
D24              0.00089000 sec
IN0              0.00002410 sec
TDav                      1
ZGOPTNS
SFO1            500.1323506 MHz
NUC1                     1H
P1                    13.35 usec
P2                    26.70 usec
PLW1            16.00000000 W
SFO2            125.7665916 MHz
NUC2                    13C
CPDPRG[2 bi_p5m4sp_4sp.2
P3                     8.90 usec
P14                  500.00 usec
P24                 2000.00 usec
P63                 1500.00 usec
PLW0                      0 W
PLW2           103.00000000 W
PLW12            1.66499996 W
SPNAM[3] Crp60,0.5,20.1
SPOAL3                0.500
SPOFFS3                   0 Hz
SPW3            12.46500015 W
SPNAM[7]     Crp60comp.4
SPOAL7                0.500
SPOFFS7                   0 Hz
SPW7            12.46500015 W
SPNAM[14 Crp32,1.5,20.2
SPOAL14               0.500
SPOFFS14                  0 Hz
SPW14            5.31860018 W
SPNAM[31 Crp32,1.5,20.2
SPOAL31               0.500
SPOFFS31                  0 Hz
SPW31            1.32969999 W
GPNAM[1]       SMSQ10.100
GPZ1                  80.00 %
GPNAM[2]       SMSQ10.100
GPZ2                  20.10 %
GPNAM[3]       SMSQ10.100
GPZ3                  11.00 %
GPNAM[4]       SMSQ10.100
GPZ4                  -5.00 %
P16                 1000.00 usec
P19                  600.00 usec

F1 - Acquisition parameters
TD                       46
SFO1               125.7666 MHz
FIDRES           902.038635 Hz
SW                  164.963 ppm
FnMODE       Echo-Antiecho

F2 - Processing parameters
SI                     1024
SF              500.1300000 MHz
WDW                   QSINE
SSB                       2
LB                        0 Hz
GB                        0
PC                     1.40

F1 - Processing parameters
SI                     1024
MC2           echo-antiecho
SF              125.7577890 MHz
WDW                   QSINE
SSB                       2
LB                        0 Hz
GB                        0
```

SSK-32-SKM-BAC-ANTH-NHAC-1H

Current Data Parameters
NAME SSK-32-SKM-BAC-ANTH-NHAC-1H
EXPNO 12
PROCNO 1

F2 - Acquisition Parameters
Date_ 20240521
Time 22.33 h
INSTRUM spect
PROBHD Z104450_0346 (
PULPROG zg30
TD 54274
SOLVENT CDCl3
NS 10
DS 0
SWH 8223.685 Hz
FIDRES 0.303043 Hz
AQ 3.2998593 sec
RG 161
DW 60.800 usec
DE 6.50 usec
TE 157.7 K
D1 1.00000000 sec
TD0 1
SFO1 400.1324710 MHz
NUC1 1H
P0 5.00 usec
P1 15.00 usec
PLW1 9.69999981 W

F2 - Processing parameters
SI 32768
SF 400.1316291 MHz
WDW EM
SSB 0
LB 0.30 Hz
GB 0
PC 1.00

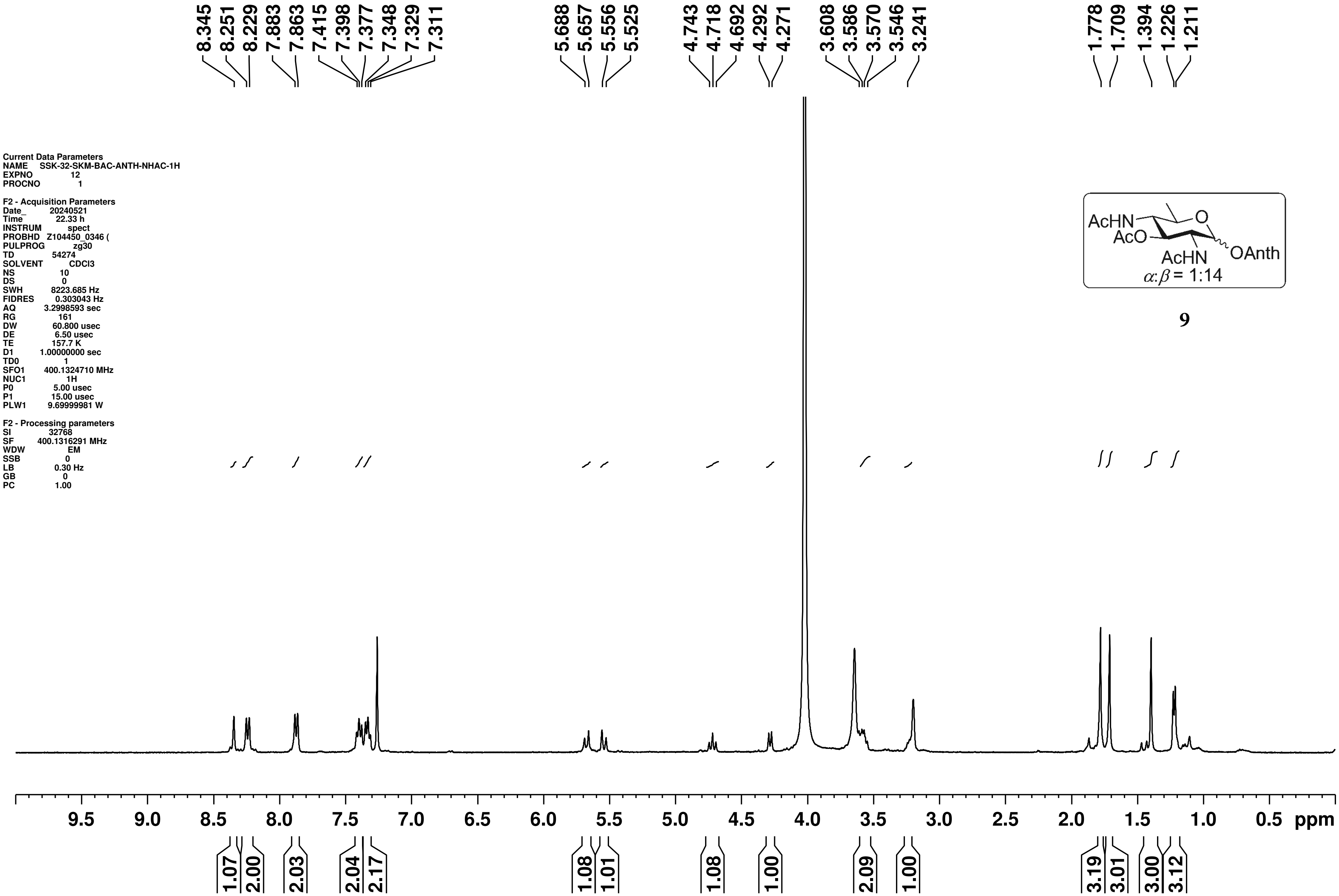

9

SSK-32-SKM-BAC-ANTH-NHAC-13

```
Current Data Parameters
NAME      SSK-32-SKM-BAC-ANTH-NHAC-13C
EXPNO                   13
PROCNO                   1

F2 - Acquisition Parameters
Date_           20240523
Time                0.45 h
INSTRUM            spect
PROBHD   Z104450_0346 (
PULPROG          zgpg30
TD                 65536
SOLVENT            CDCl3
NS                  2500
DS                     0
SWH           26041.666 Hz
FIDRES         0.794729 Hz
AQ            1.2582912 sec
RG                  1030
DW                19.200 usec
DE                  6.50 usec
TE                 172.3 K
D1           1.00000000 sec
D11          0.03000000 sec
TD0                    1
SFO1        100.6238364 MHz
NUC1                 13C
P0                  3.33 usec
P1                 10.00 usec
PLW1        47.00000000 W
SFO2        400.1316005 MHz
NUC2                  1H
CPDPRG[2         waltz16
PCPD2              90.00 usec
PLW2         9.69999981 W
PLW12        0.26944000 W
PLW13        0.13552999 W

F2 - Processing parameters
SI                 32768
SF          100.6131735 MHz
WDW                   EM
SSB                    0
LB                  1.00 Hz
GB                     0
PC                  1.00
```

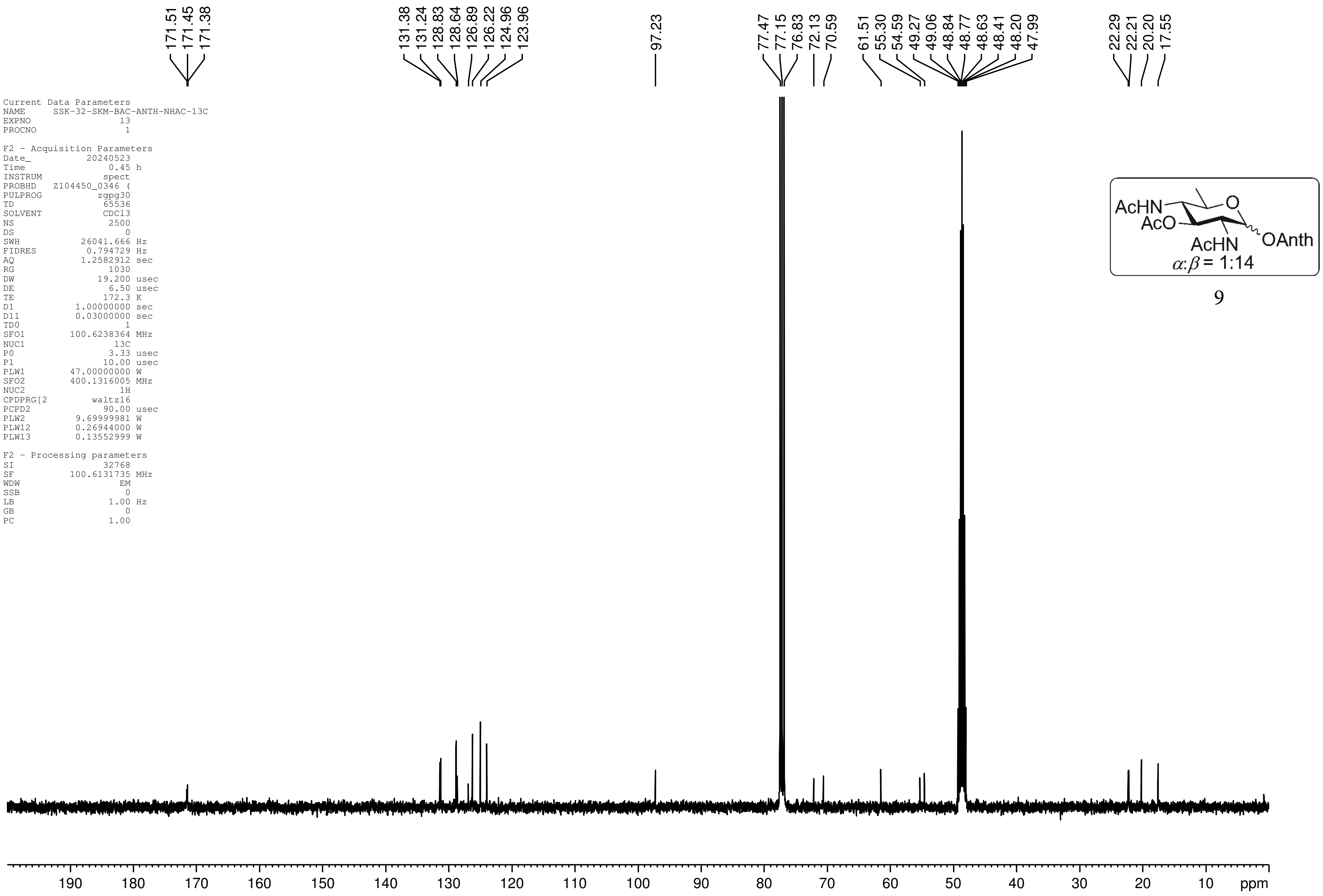

9

SSK-32-SKM-BAC-ANTH-NHAC-DEPT

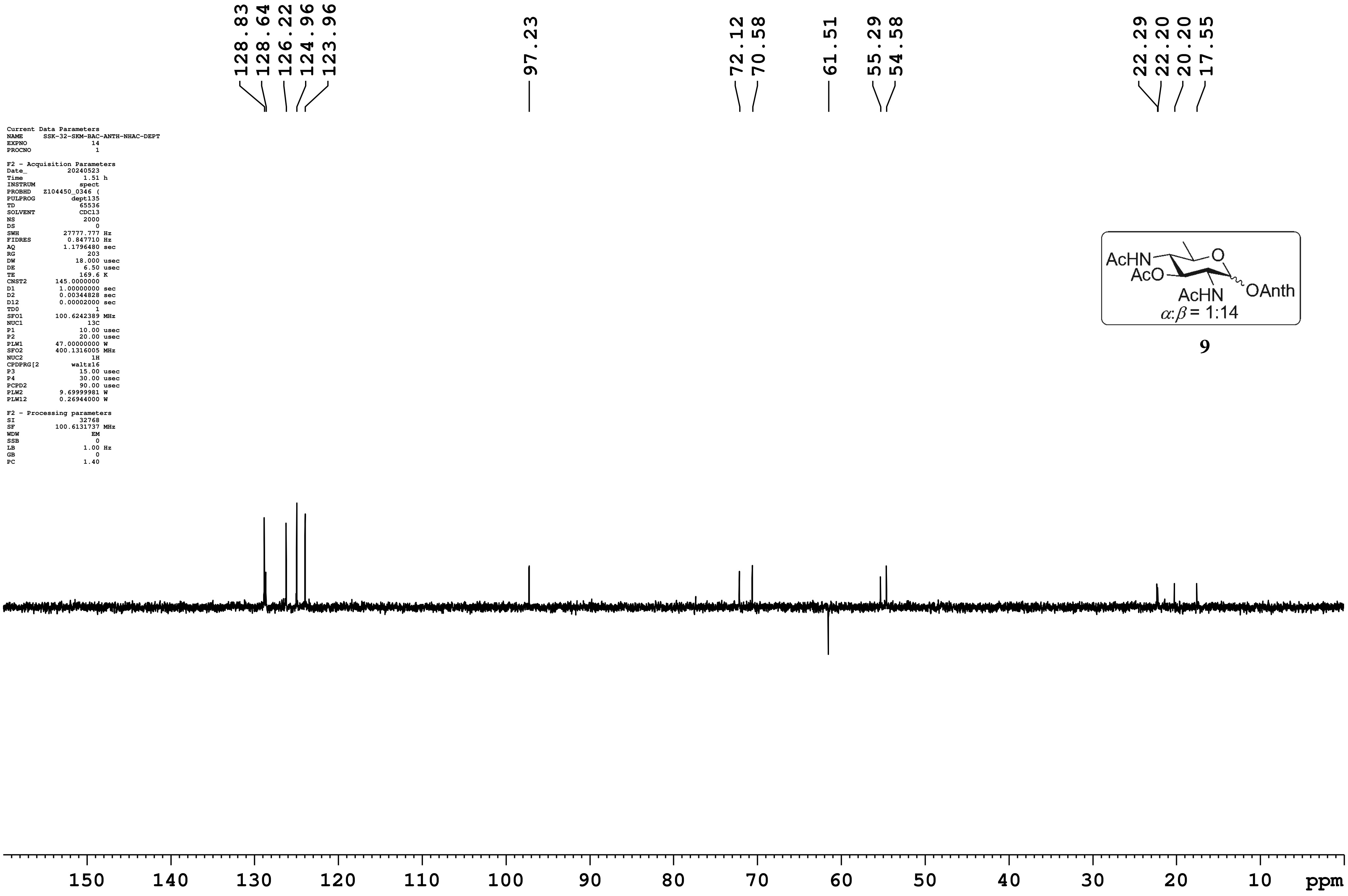

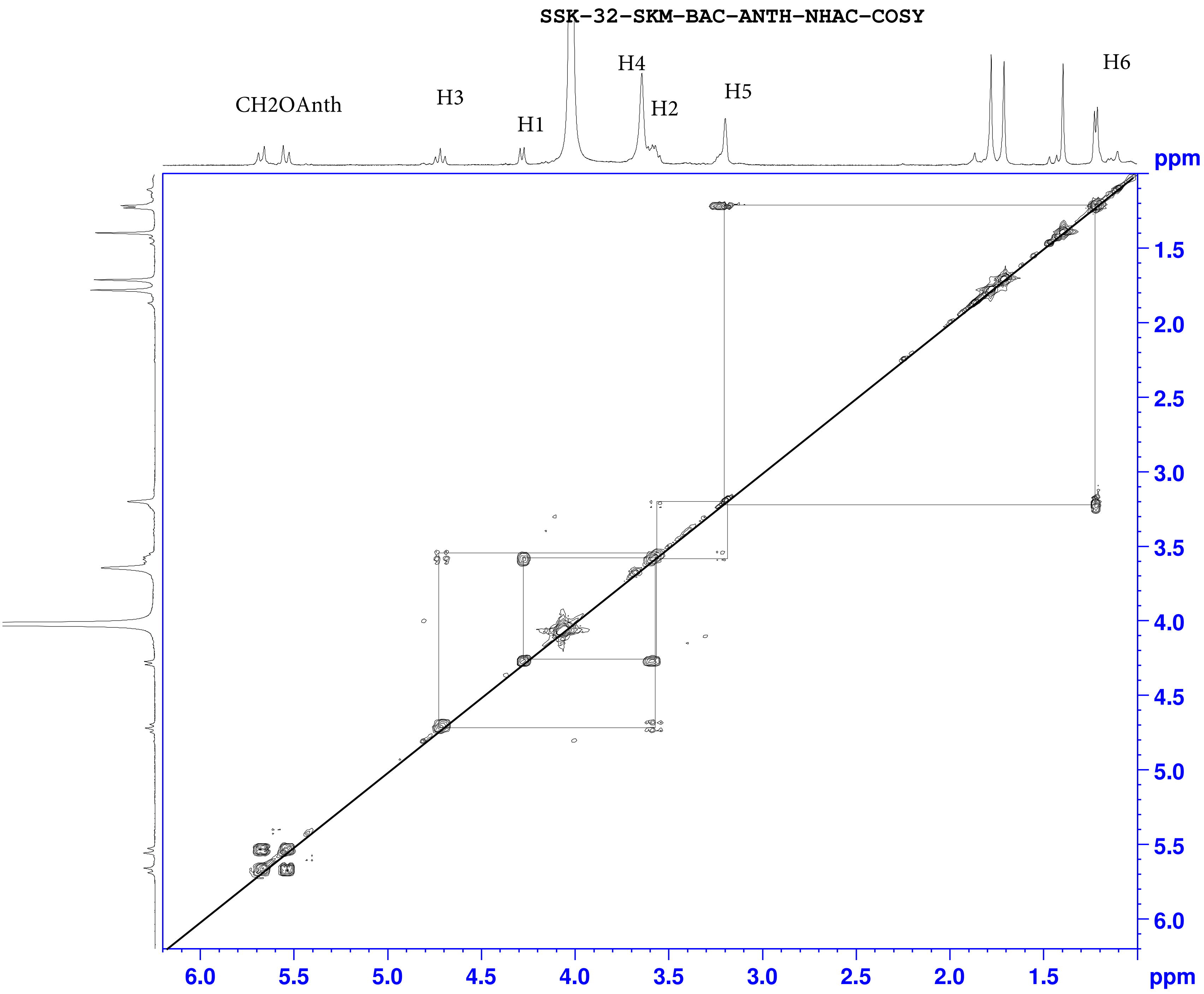

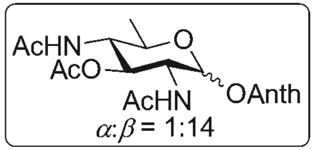

9

```
Current Data Parameters
NAME      SSK-32-SKM-BAC-ANTH-NHAC-COSY
EXPNO                    16
PROCNO                    1

F2 - Acquisition Parameters
Date_              20240523
Time                 3.49 h
INSTRUM               spect
PROBHD     Z104450_0346 (
PULPROG            cosygpqf
TD                     2048
SOLVENT               CDCl3
NS                        8
DS                        8
SWH             5341.880 Hz
FIDRES          5.216680 Hz
AQ             0.1916928 sec
RG                       64
DW               93.600 usec
DE                 6.50 usec
TE                169.6 K
D0           0.00000300 sec
D1           1.48689198 sec
D13          0.00000400 sec
D16          0.00020000 sec
IN0          0.00018720 sec
TDav                      1
SFO1        400.1324057 MHz
NUC1                     1H
P0                15.00 usec
P1                15.00 usec
PLW1          9.69999981 W
GPNAM[1]           SINE.100
GPZ1              10.00 %
P16             1000.00 usec

F1 - Acquisition parameters
TD                      512
SFO1           400.1324 MHz
FIDRES        20.866720 Hz
SW               13.350 ppm
FnMODE                   QF

F2 - Processing parameters
SI                     1024
SF           400.1316297 MHz
WDW                    SINE
SSB                       0
LB                      0 Hz
GB                        0
PC                     1.40

F1 - Processing parameters
SI                     1024
MC2                      QF
SF           400.1316296 MHz
WDW                    SINE
SSB                       0
LB                      0 Hz
GB                        0
```

SSK-32-SKM-346-2-1H

Current Data Parameters
NAME SSK-32-SKM-346-2-1H
EXPNO 16
PROCNO 1

F2 - Acquisition Parameters
Date_ 20231002
Time 19.29 h
INSTRUM spect
PROBHD Z104450_0346 (
PULPROG zg30
TD 54274
SOLVENT CDCl3
NS 5
DS 0
SWH 8223.685 Hz
FIDRES 0.303043 Hz
AQ 3.2998593 sec
RG 80.6
DW 60.800 usec
DE 6.50 usec
TE 161.3 K
D1 1.00000000 sec
TD0 1
SFO1 400.1324710 MHz
NUC1 1H
P0 5.00 usec
P1 15.00 usec
PLW1 9.69999981 W

F2 - Processing parameters
SI 32768
SF 400.1300095 MHz
WDW EM
SSB 0
LB 0.30 Hz
GB 0
PC 1.00

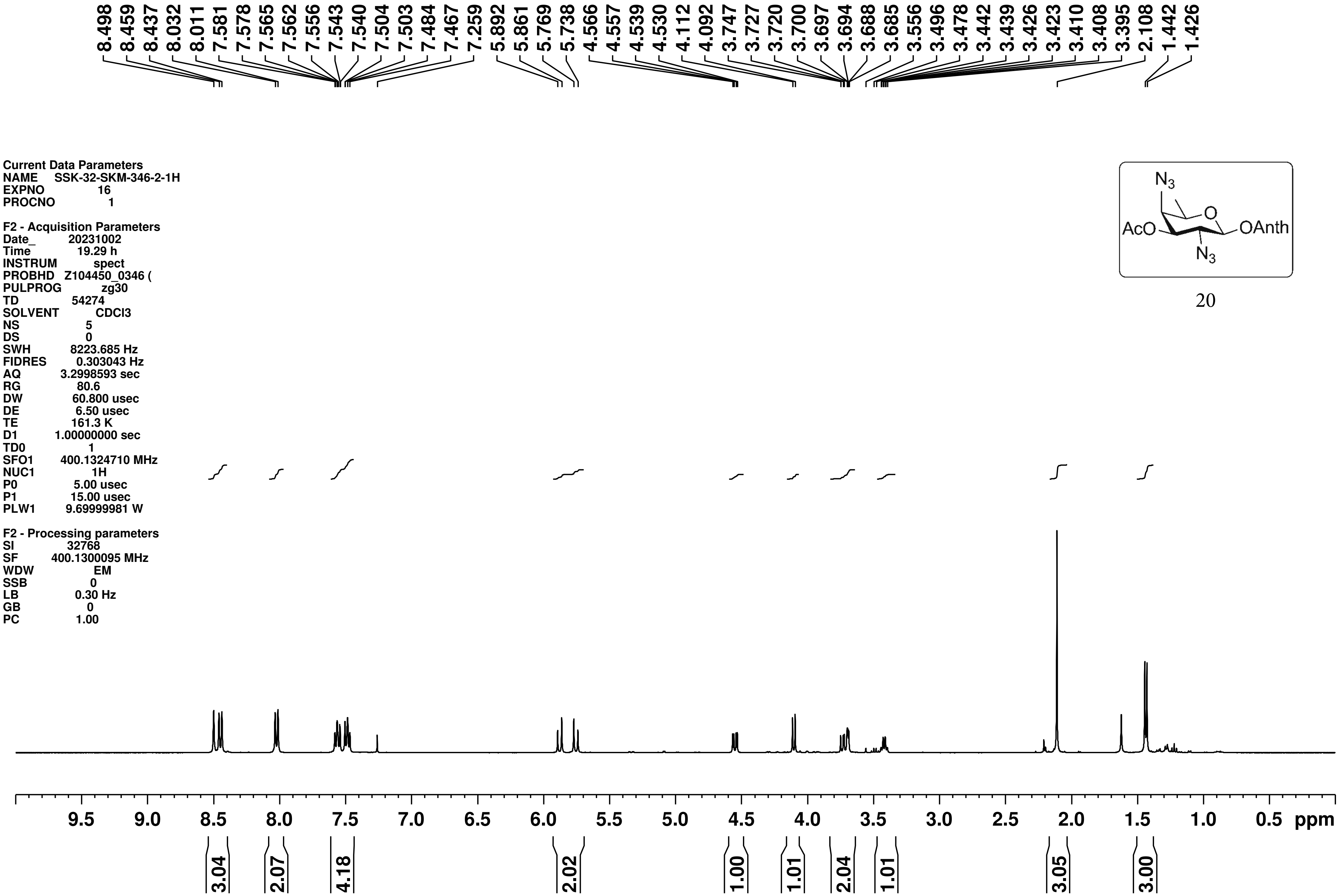

SSK-32-SKM-346-2-13C

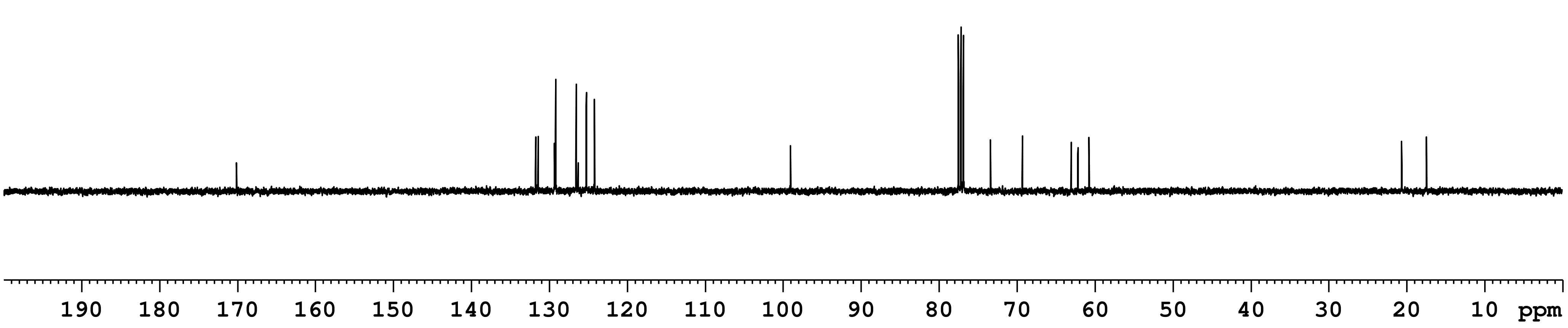


```
Current Data Parameters
NAME      SSK-32-SKM-346-2-13C
EXPNO                   17
PROCNO                   1

F2 - Acquisition Parameters
Date_             20231002
Time                 19.31 h
INSTRUM              spect
PROBHD   Z104450_0346 (
PULPROG            zgpg30
TD                   65536
SOLVENT              CDCl3
NS                      44
DS                       0
SWH              26041.666 Hz
FIDRES            0.794729 Hz
AQ               1.2582912 sec
RG                    1030
DW                  19.200 usec
DE                    6.50 usec
TE                   161.3 K
D1              1.00000000 sec
D11             0.03000000 sec
TD0                      1
SFO1           100.6238364 MHz
NUC1                   13C
P0                    3.33 usec
P1                   10.00 usec
PLW1           47.00000000 W
SFO2           400.1316005 MHz
NUC2                    1H
CPDPRG[2           waltz16
PCPD2                90.00 usec
PLW2            9.69999981 W
PLW12           0.26944000 W
PLW13           0.13552999 W

F2 - Processing parameters
SI                   32768
SF             100.6127608 MHz
WDW                     EM
SSB                      0
LB                    1.00 Hz
GB                       0
PC                    1.40
```

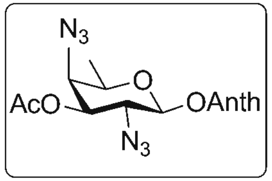

20

SSK-32-SKM-346-2-DEPT

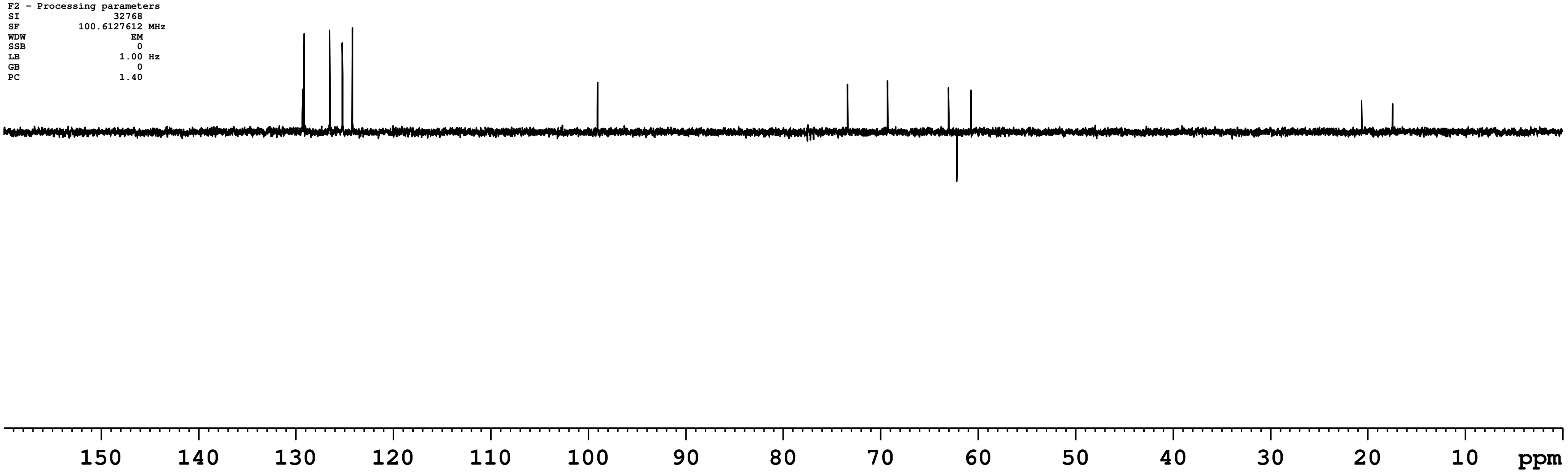


```
Current Data Parameters
NAME       SSK-32-SKM-346-2-DEPT
EXPNO                 18
PROCNO                 1

F2 - Acquisition Parameters
Date_           20231002
Time               19.33 h
INSTRUM            spect
PROBHD   Z104450_0346 (
PULPROG          dept135
TD                 65536
SOLVENT            CDCl3
NS                    55
DS                     0
SWH            27777.777 Hz
FIDRES          0.847710 Hz
AQ             1.1796480 sec
RG                   203
DW                18.000 usec
DE                  6.50 usec
TE                 161.3 K
CNST2        145.0000000
D1            1.00000000 sec
D2            0.00344828 sec
D12           0.00002000 sec
TD0                    1
SFO1         100.6242389 MHz
NUC1                 13C
P1                 10.00 usec
P2                 20.00 usec
PLW1         47.00000000 W
SFO2         400.1316005 MHz
NUC2                  1H
CPDPRG[2         waltz16
P3                 15.00 usec
P4                 30.00 usec
PCPD2              90.00 usec
PLW2          9.69999981 W
PLW12         0.26944000 W

F2 - Processing parameters
SI                 32768
SF           100.6127612 MHz
WDW                   EM
SSB                    0
LB                  1.00 Hz
GB                     0
PC                  1.40
```

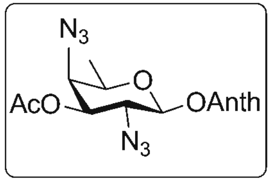

20

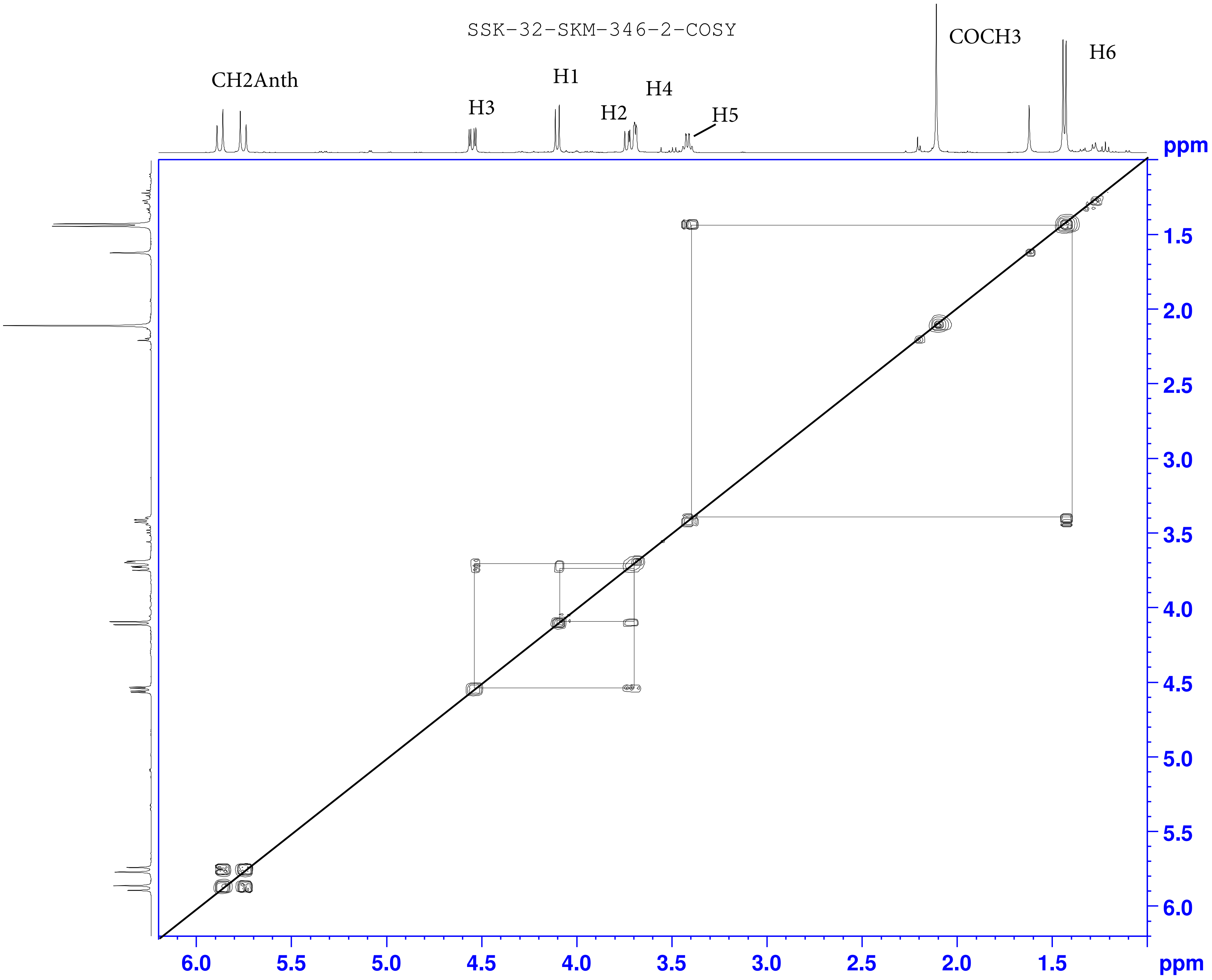

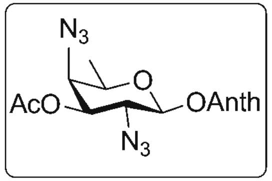

20

```
Current Data Parameters
NAME       SSK-32-SKM-346-2-COSY
EXPNO                     19
PROCNO                     1

F2 - Acquisition Parameters
Date_             20231002
Time                 19.44 h
INSTRUM              spect
PROBHD     Z104450_0346 (
PULPROG           cosygpqf
TD                    2048
SOLVENT              CDCl3
NS                       4
DS                       0
SWH               5341.880 Hz
FIDRES            5.216680 Hz
AQ               0.1916928 sec
RG                      64
DW                  93.600 usec
DE                    6.50 usec
TE                   161.3 K
D0              0.00000300 sec
D1              1.00000000 sec
D13             0.00000400 sec
D16             0.00020000 sec
IN0             0.00018720 sec
TDav                     1
SFO1           400.1324057 MHz
NUC1                    1H
P0                   15.00 usec
P1                   15.00 usec
PLW1            9.69999981 W
GPNAM[1]          SINE.100
GPZ1                 10.00 %
P16                1000.00 usec

F1 - Acquisition parameters
TD                     126
SFO1              400.1324 MHz
FIDRES           84.791748 Hz
SW                  13.350 ppm
FnMODE                  QF

F2 - Processing parameters
SI                    1024
SF             400.1300145 MHz
WDW                   SINE
SSB                      0
LB                       0 Hz
GB                       0
PC                    1.40

F1 - Processing parameters
SI                    1024
MC2                     QF
SF             400.1300060 MHz
WDW                   SINE
SSB                      0
LB                       0 Hz
GB                       0
```

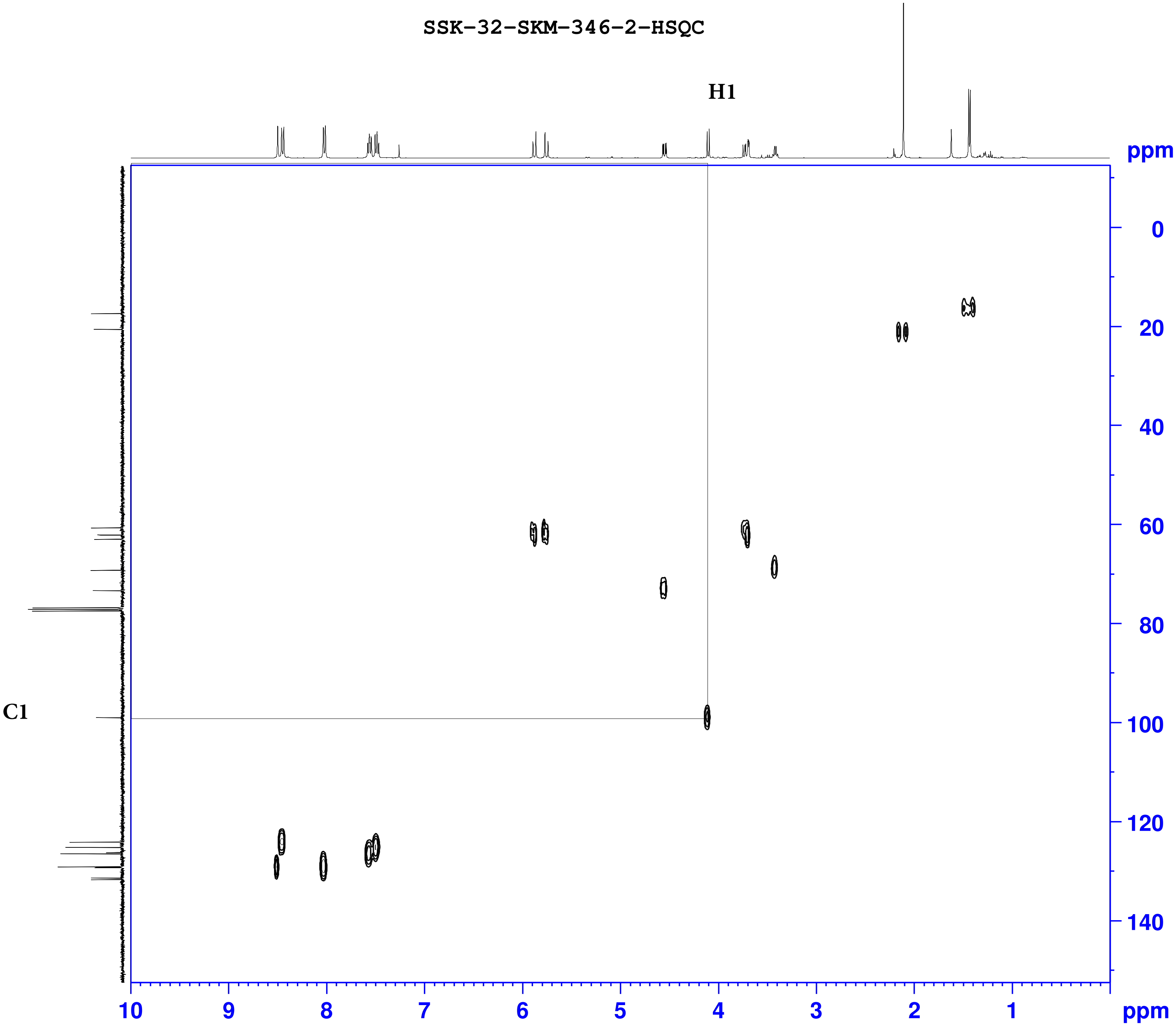

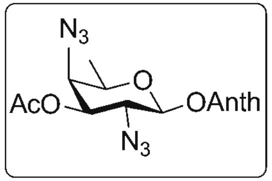

20

Current Data Parameters
NAME SSK-32-SKM-346-2-HSQC
EXPNO 20
PROCNO 1

F2 - Acquisition Parameters
Date_ 20231002
Time 19.47 h
INSTRUM spect
PROBHD Z104450_0346 (
PULPROG hsqcetgp
TD 2048
SOLVENT CDCl3
NS 2
DS 0
SWH 8802.817 Hz
FIDRES 8.596501 Hz
AQ 0.1163264 sec
RG 2050
DW 56.800 usec
DE 6.50 usec
TE 160.8 K
CNST2 145.0000000
D0 0.00000300 sec
D1 1.00000000 sec
D4 0.00172414 sec
D11 0.03000000 sec
D16 0.00020000 sec
IN0 0.00001800 sec
TDav 1
ZGOPTNS
SFO1 400.1324708 MHz
NUC1 1H
P1 15.00 usec
P2 30.00 usec
PLW1 9.69999981 W
SFO2 100.6248425 MHz
NUC2 13C
CPDPRG[2 garp
P3 10.00 usec
P4 20.00 usec
PCPD2 80.00 usec
PLW2 47.00000000 W
PLW12 0.73438001 W
GPNAM[1] SINE.100
GPZ1 80.00 %
GPNAM[2] SINE.100
GPZ2 20.10 %
P16 1000.00 usec

F1 - Acquisition parameters
TD 76
SFO1 100.6248 MHz
FIDRES 730.994141 Hz
SW 276.053 ppm
FnMODE Echo-Antiecho

F2 - Processing parameters
SI 2048
SF 400.1300052 MHz
WDW QSINE
SSB 2
LB 0 Hz
GB 0
PC 1.40

F1 - Processing parameters
SI 1024
MC2 echo-antiecho
SF 100.6127690 MHz
WDW QSINE
SSB 2
LB 0 Hz
GB 0

SSK-32-SKM-DAT-ANTH-NHAC-1H

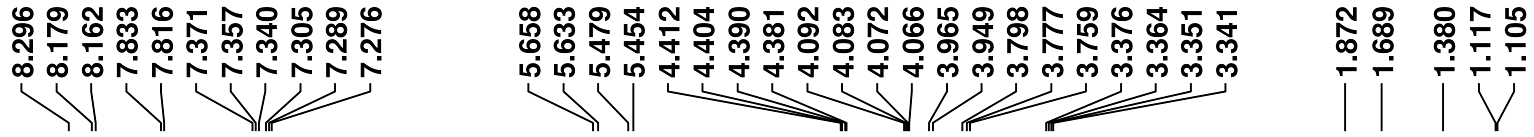

Current Data Parameters
NAME SSK-32-SKM-DAT-ANTH-NHAC-1H
EXPNO 17
PROCNO 1

F2 - Acquisition Parameters
Date_ 20250612
Time 10.04 h
INSTRUM spect
PROBHD Z119470_0087 (
PULPROG zg30
TD 65536
SOLVENT CDCl3
NS 16
DS 0
SWH 10000.000 Hz
FIDRES 0.305176 Hz
AQ 3.2767999 sec
RG 85.91
DW 50.000 usec
DE 6.50 usec
TE 296.0 K
D1 1.00000000 sec
TD0 1
SFO1 500.1330885 MHz
NUC1 1H
P0 4.45 usec
P1 13.35 usec
PLW1 16.00000000 W

F2 - Processing parameters
SI 65536
SF 500.1320664 MHz
WDW EM
SSB 0
LB 0.30 Hz
GB 0
PC 1.00

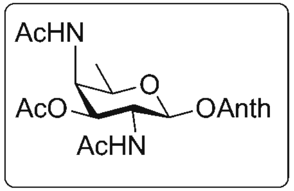

10

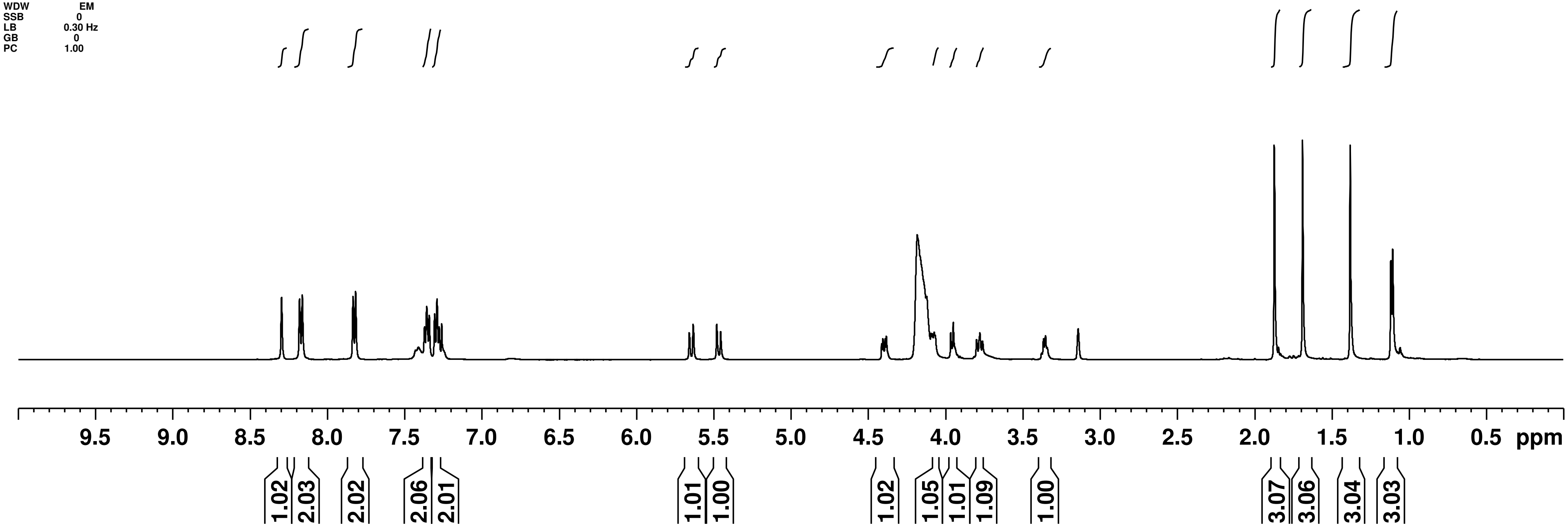

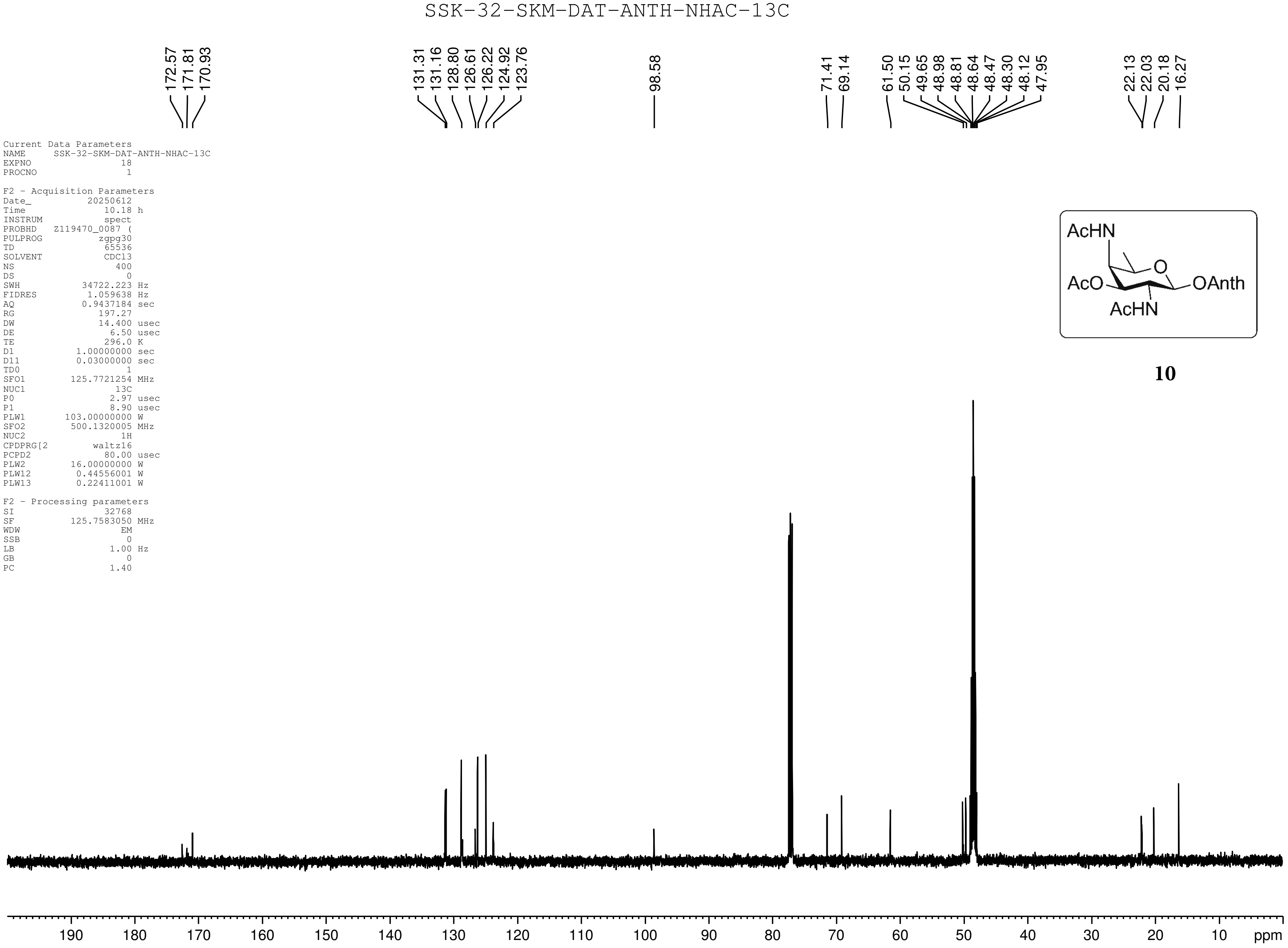
SSK-32-SKM-DAT-ANTH-NHAC-13C
172.57
171.81
170.93
131.31
131.16
128.80
126.61
126.22
124.92
123.76
98.58
71.41
69.14
61.50
50.15
49.65
48.98
48.81
48.64
48.47
48.30
48.12
47.95
22.13
22.03
20.18
16.27
Current Data Parameters
NAME SSK-32-SKM-DAT-ANTH-NHAC-13C
EXPNO 18
PROCNO 1
F2 - Acquisition Parameters
Date_ 20250612
Time 10.18 h
INSTRUM spect
PROBHD Z119470_0087 (
PULPROG zgpg30
TD 65536
SOLVENT CDCl3
NS 400
DS 0
SWH 34722.223 Hz
FIDRES 1.059638 Hz
AQ 0.9437184 sec
RG 197.27
DW 14.400 usec
DE 6.50 usec
TE 296.0 K
D1 1.00000000 sec
D11 0.03000000 sec
TD0 1
SFO1 125.7721254 MHz
NUC1 13C
P0 2.97 usec
P1 8.90 usec
PLW1 103.00000000 W
SFO2 500.1320005 MHz
NUC2 1H
CPDPRG[2 waltz16
PCPD2 80.00 usec
PLW2 16.00000000 W
PLW12 0.44556001 W
PLW13 0.22411001 W
F2 - Processing parameters
SI 32768
SF 125.7583050 MHz
WDW EM
SSB 0
LB 1.00 Hz
GB 0
PC 1.40
190
180
170
160
150
140
130
120
110
100
90
80
70
60
50
40
30
20
10
ppm
AcHN
O
AcO
OAnth
AcHN

10

SSK-32-SKM-DAT-ANTH-NHAC-DEPT

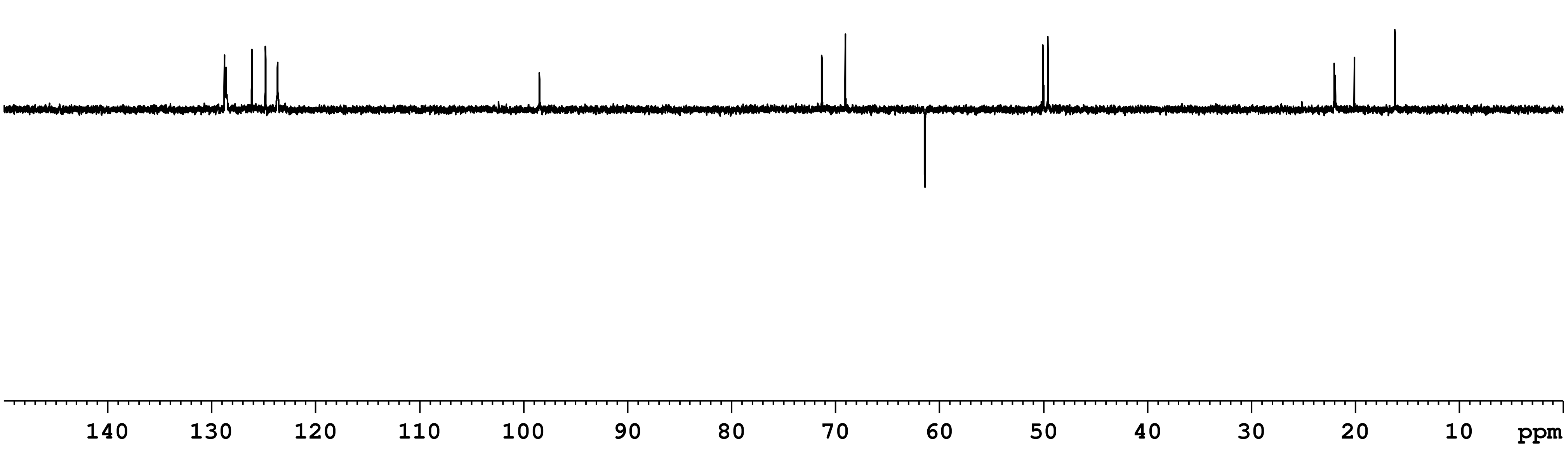


```
Current Data Parameters
NAME     SSK-32-SKM-DAT-ANTH-NHAC-DEPT
EXPNO              19
PROCNO              1

F2 - Acquisition Parameters
Date_          20250612
Time              10.31 h
INSTRUM           spect
PROBHD   Z119470_0087 (
PULPROG       deptsp135
TD                65536
SOLVENT           CDCl3
NS                  300
DS                    0
SWH           20161.291 Hz
FIDRES         0.615274 Hz
AQ            1.6252928 sec
RG               197.27
DW               24.800 usec
DE                 6.50 usec
TE                296.9 K
CNST2       145.0000000
D1           1.00000000 sec
D2           0.00344828 sec
D12          0.00002000 sec
TD0                   1
SFO1        125.7678486 MHz
NUC1                13C
P1                 8.90 usec
P13             2000.00 usec
PLW0                  0 W
PLW1       103.00000000 W
SPNAM[5]    Crp60comp.4
SPOAL5            0.500
SPOFFS5               0 Hz
SPW5        12.46500015 W
SFO2        500.1315995 MHz
NUC2                 1H
CPDPRG[2        waltz16
P3                13.35 usec
P4                26.70 usec
PCPD2             80.00 usec
PLW2        16.00000000 W
PLW12        0.44556001 W

F2 - Processing parameters
SI                32768
SF          125.7583187 MHz
WDW                  EM
SSB                   0
LB                 1.00 Hz
GB                    0
PC                 1.40
```

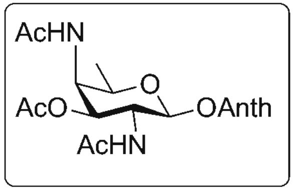

10

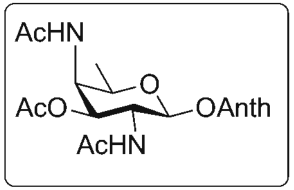

10

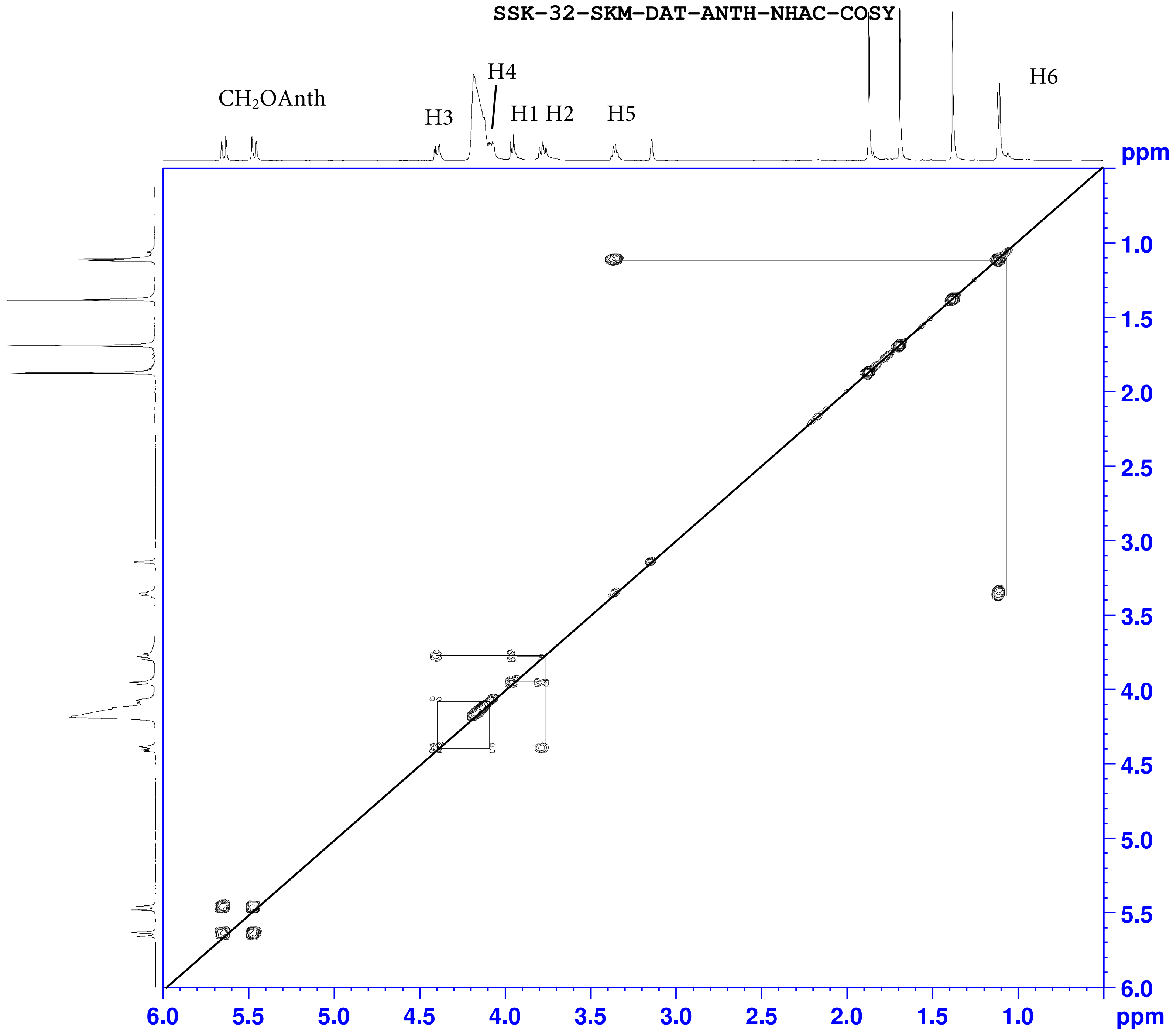

Current Data Parameters

NAME	SSK-32-SKM-DAT-ANTH-NHAC-COSY
EXPNO	21
PROCNO	1

F2 - Acquisition Parameters

Date_	20250612
Time	11.07 h
INSTRUM	spect
PROBHD	Z119470_0087 (
PULPROG	cosygpppqf
TD	2048
SOLVENT	CDCl3
NS	4
DS	0
SWH	6684.492 Hz
FIDRES	6.527824 Hz
AQ	0.1531904 sec
RG	61.42
DW	74.800 usec
DE	6.50 usec
TE	296.7 K
D0	0.00000300 sec
D1	1.00000000 sec
D11	0.03000000 sec
D12	0.00002000 sec
D13	0.00000400 sec
D16	0.00020000 sec
IN0	0.00014960 sec
TDav	1
SFO1	500.1330069 MHz
NUC1	1H
P0	13.35 usec
P1	13.35 usec
P17	2500.00 usec
PLW1	16.00000000 W
PLW10	3.16840005 W
GPNAM[1]	SMSQ10.100
GPZ1	10.00 %
P16	1000.00 usec

F1 - Acquisition parameters

TD	350
SFO1	500.133 MHz
FIDRES	38.197098 Hz
SW	13.365 ppm
FnMODE	QF

F2 - Processing parameters

SI	1024
SF	500.1320635 MHz
WDW	QSINE
SSB	0
LB	0 Hz
GB	0
PC	1.40

F1 - Processing parameters

SI	1024
MC2	QF
SF	500.1320659 MHz
WDW	QSINE
SSB	0
LB	0 Hz
GB	0

SSK-32-SKM-FUC-ANTH-1H

Current Data Parameters
NAME SSK-32-SKM-FUC-ANTH-1H
EXPNO 13
PROCNO 1

F2 - Acquisition Parameters
Date_ 20240518
Time 15.17 h
INSTRUM spect
PROBHD Z104450_0346 (
PULPROG zg30
TD 54274
SOLVENT CDCl3
NS 21
DS 0
SWH 8223.685 Hz
FIDRES 0.303043 Hz
AQ 3.2998593 sec
RG 161
DW 60.800 usec
DE 6.50 usec
TE 167.1 K
D1 1.00000000 sec
TD0 1
SFO1 400.1324710 MHz
NUC1 1H
P0 5.00 usec
P1 15.00 usec
PLW1 9.69999981 W

F2 - Processing parameters
SI 32768
SF 400.1300095 MHz
WDW EM
SSB 0
LB 0.30 Hz
GB 0
PC 1.00

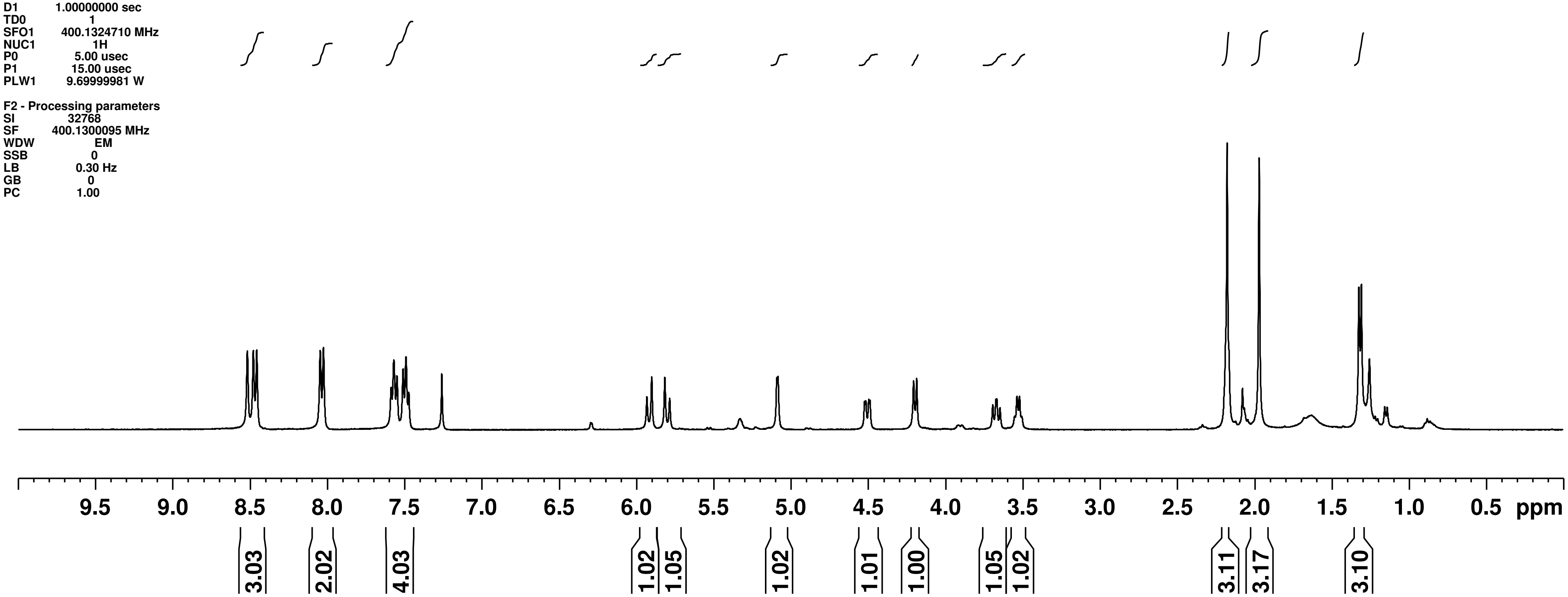

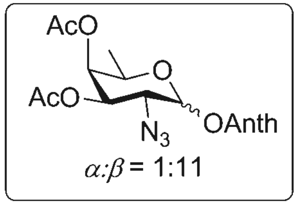

21

SSK-32-SKM-FUC-ANTH-13C

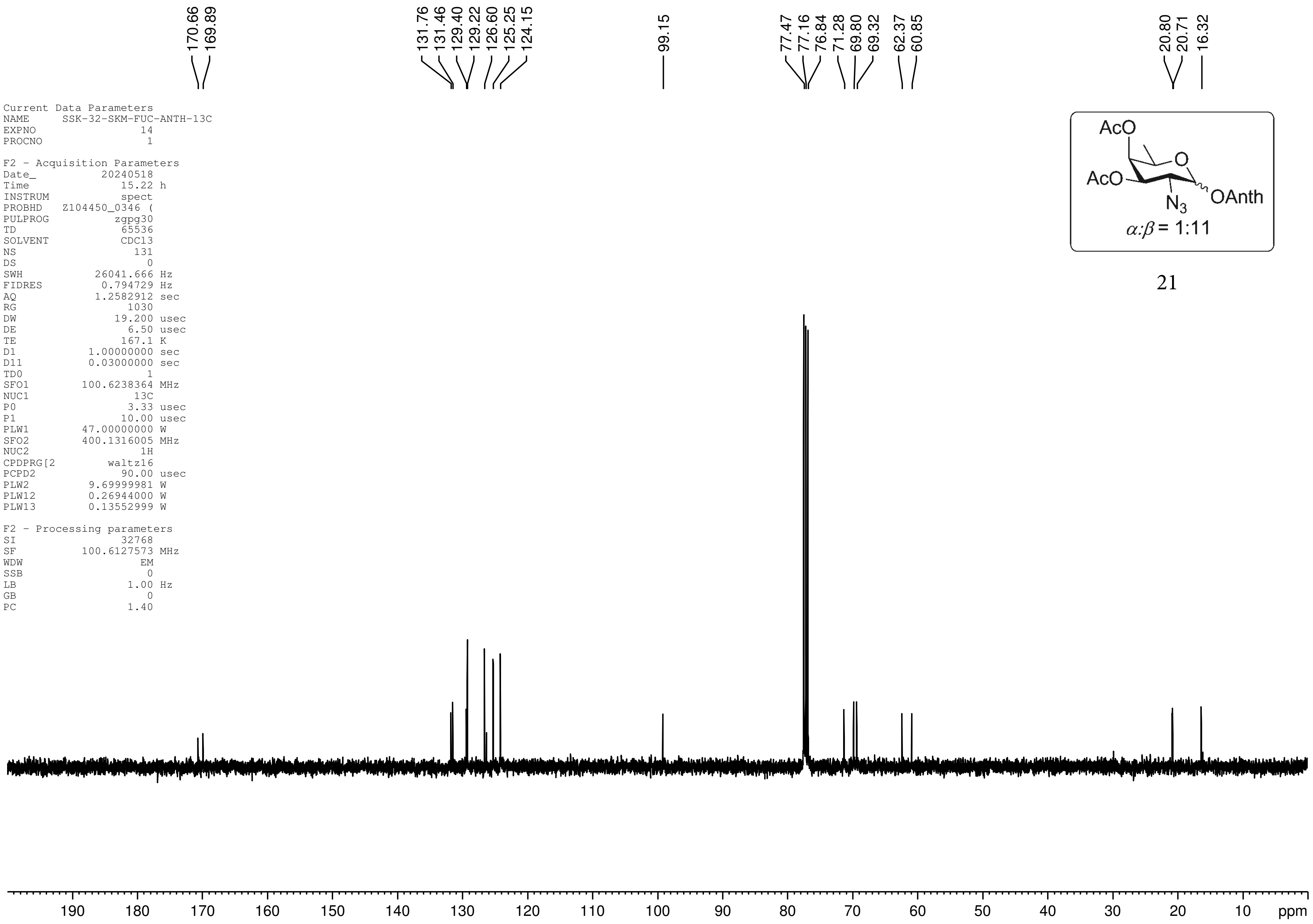

21

SSK-32-SKM-FUC-ANTH-DEPT

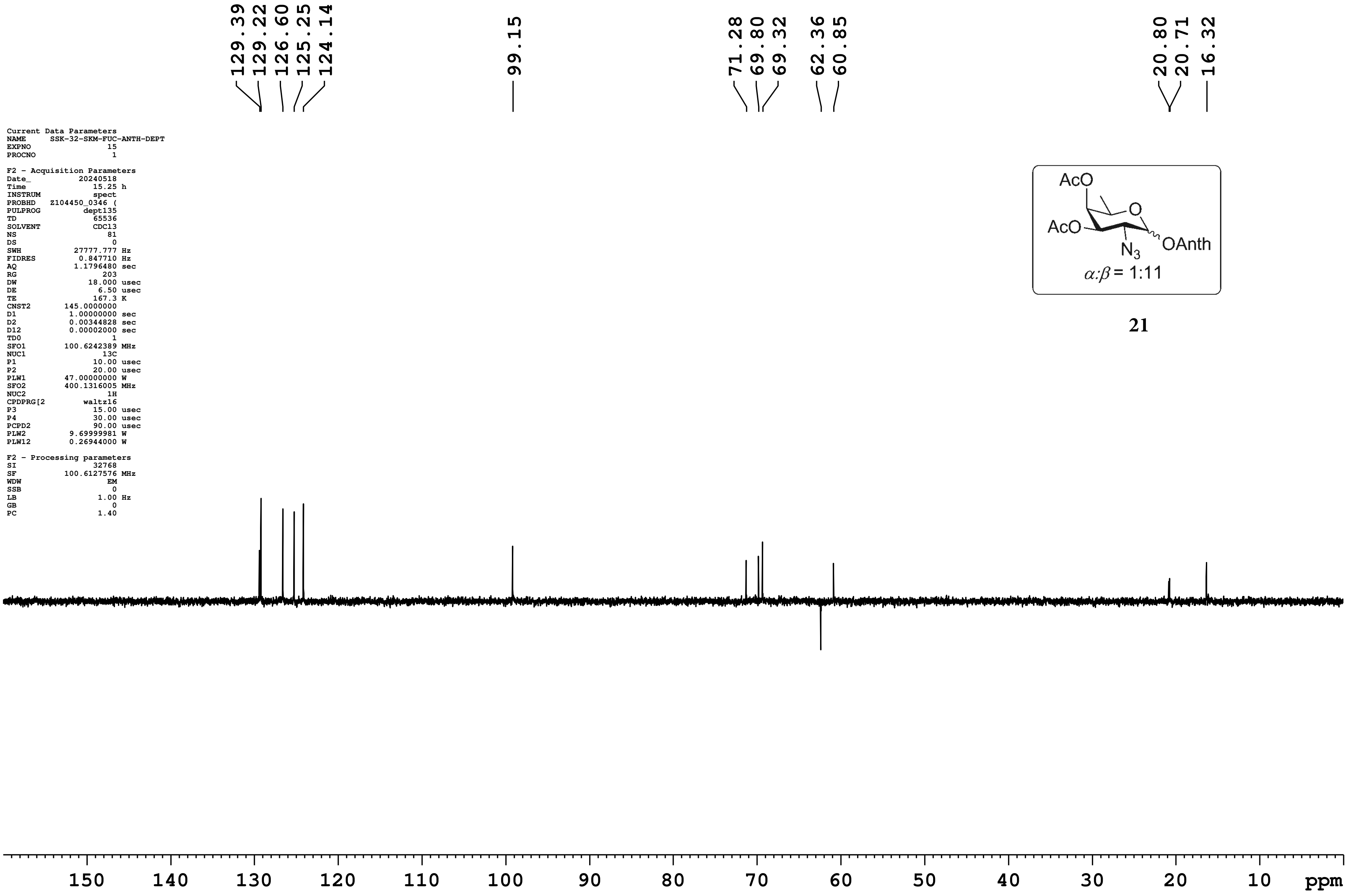

21

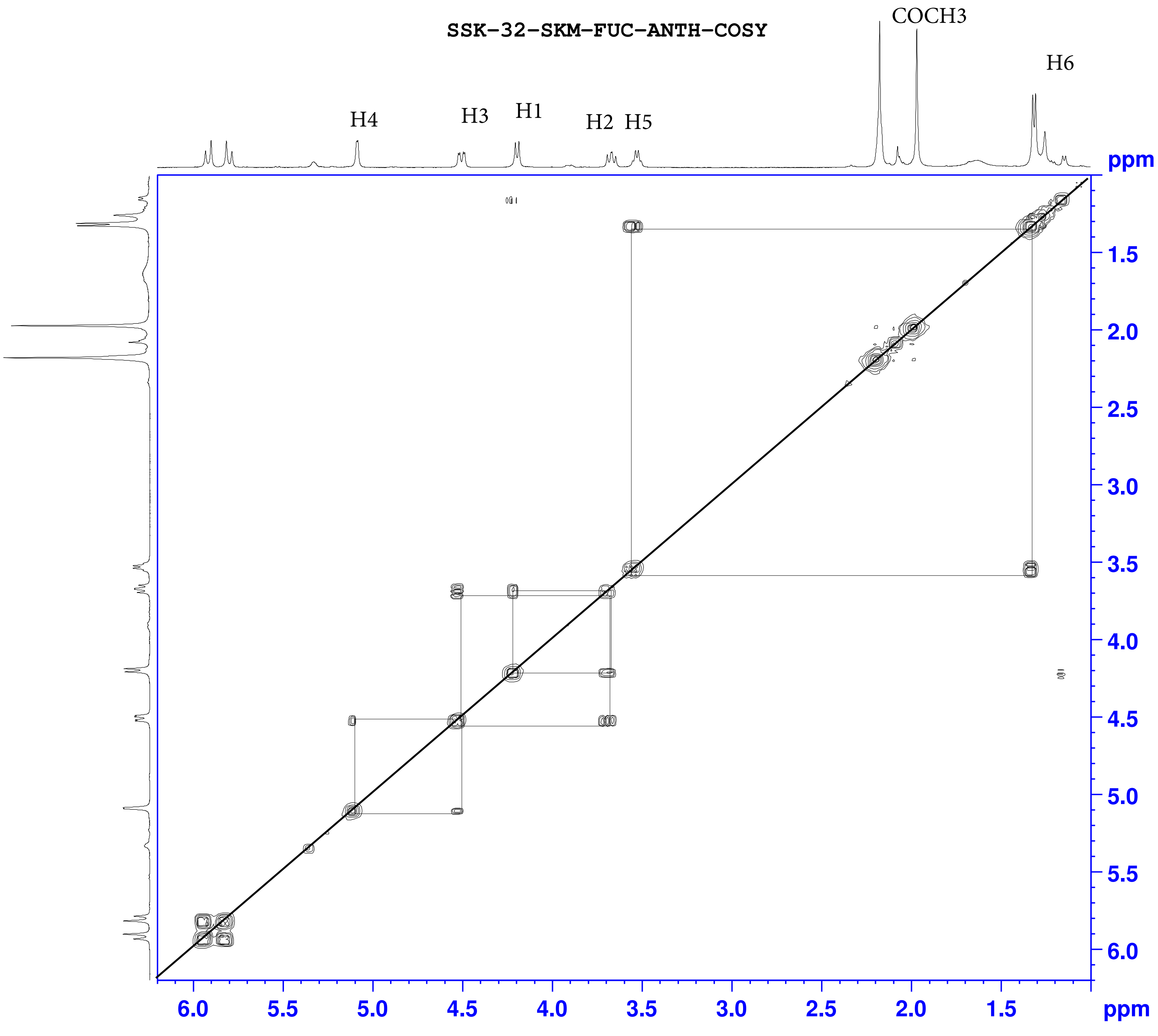

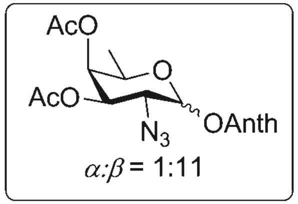

21

Current Data Parameters
NAME SSK-32-SKM-FUC-ANTH-COSY
EXPNO 17
PROCNO 1

F2 - Acquisition Parameters
Date_ 20240518
Time 15.39 h
INSTRUM spect
PROBHD Z104450_0346 (
PULPROG cosygpqf
TD 2048
SOLVENT CDCl3
NS 4
DS 0
SWH 5341.880 Hz
FIDRES 5.216680 Hz
AQ 0.1916928 sec
RG 64
DW 93.600 usec
DE 6.50 usec
TE 167.3 K
D0 0.00000300 sec
D1 1.00000000 sec
D13 0.00000400 sec
D16 0.00020000 sec
IN0 0.00018720 sec
TDav 1
SFO1 400.1324057 MHz
NUC1 1H
P0 15.00 usec
P1 15.00 usec
PLW1 9.69999981 W
GPNAM[1] SINE.100
GPZ1 10.00 %
P16 1000.00 usec

F1 - Acquisition parameters
TD 128
SFO1 400.1324 MHz
FIDRES 83.466881 Hz
SW 13.350 ppm
FnMODE QF

F2 - Processing parameters
SI 1024
SF 400.1300000 MHz
WDW SINE
SSB 0
LB 0 Hz
GB 0
PC 1.40

F1 - Processing parameters
SI 1024
MC2 QF
SF 400.1300000 MHz
WDW SINE
SSB 0
LB 0 Hz
GB 0

SSK-32-SKM-FUC-ANTH-HSQC

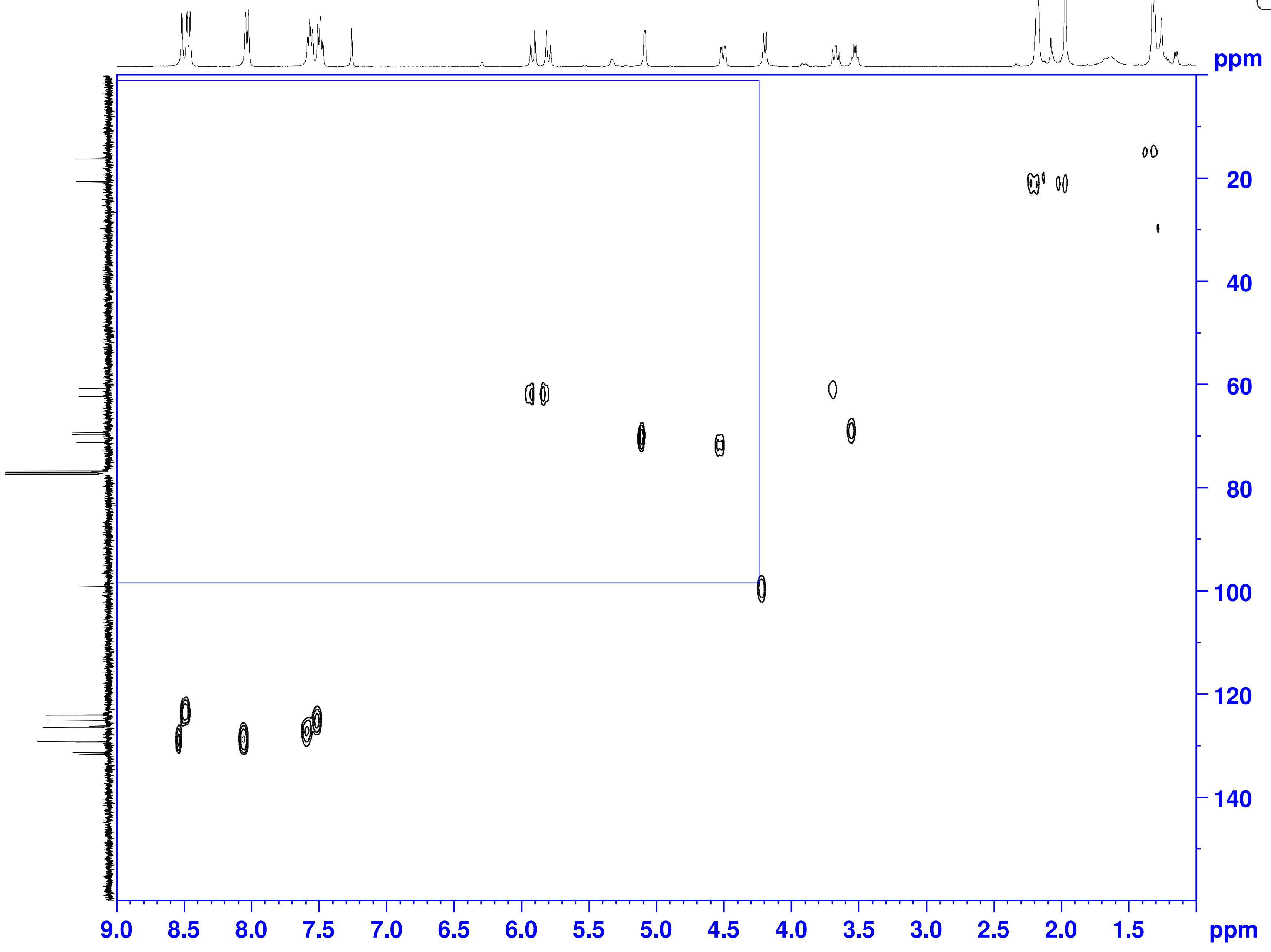

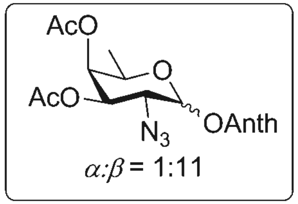

21

```
Current Data Parameters
NAME      SSK-32-SKM-FUC-ANTH-HSQC
EXPNO                   16
PROCNO                   1

F2 - Acquisition Parameters
Date_             20240518
Time                 15.29 h
INSTRUM              spect
PROBHD    Z104450_0346 (
PULPROG           hsqcetgp
TD                    2048
SOLVENT              CDCl3
NS                       2
DS                       0
SWH               8802.817 Hz
FIDRES            8.596501 Hz
AQ               0.1163264 sec
RG                    2050
DW                  56.800 usec
DE                    6.50 usec
TE                   167.3 K
CNST2          145.0000000
D0              0.00000300 sec
D1              1.00000000 sec
D4              0.00172414 sec
D11             0.03000000 sec
D16             0.00020000 sec
IN0             0.00001800 sec
TDav                     1
ZGOPTNS
SFO1           400.1324708 MHz
NUC1                    1H
P1                   15.00 usec
P2                   30.00 usec
PLW1            9.69999981 W
SFO2           100.6248425 MHz
NUC2                   13C
CPDPRG[2              garp
P3                   10.00 usec
P4                   20.00 usec
PCPD2                80.00 usec
PLW2           47.00000000 W
PLW12           0.73438001 W
GPNAM[1]         SINE.100
GPZ1                 80.00 %
GPNAM[2]         SINE.100
GPZ2                 20.10 %
P16                1000.00 usec

F1 - Acquisition parameters
TD                      87
SFO1              100.6248 MHz
FIDRES          638.569580 Hz
SW                 276.053 ppm
FnMODE        Echo-Antiecho

F2 - Processing parameters
SI                    2048
SF             400.1300000 MHz
WDW                  QSINE
SSB                      2
LB                       0 Hz
GB                       0
PC                    1.40

F1 - Processing parameters
SI                    1024
MC2           echo-antiecho
SF             100.6127690 MHz
WDW                  QSINE
SSB                      2
LB                       0 Hz
GB                       0
```

SSK-32-SKM-FUC-ANTH-NHAC-1H

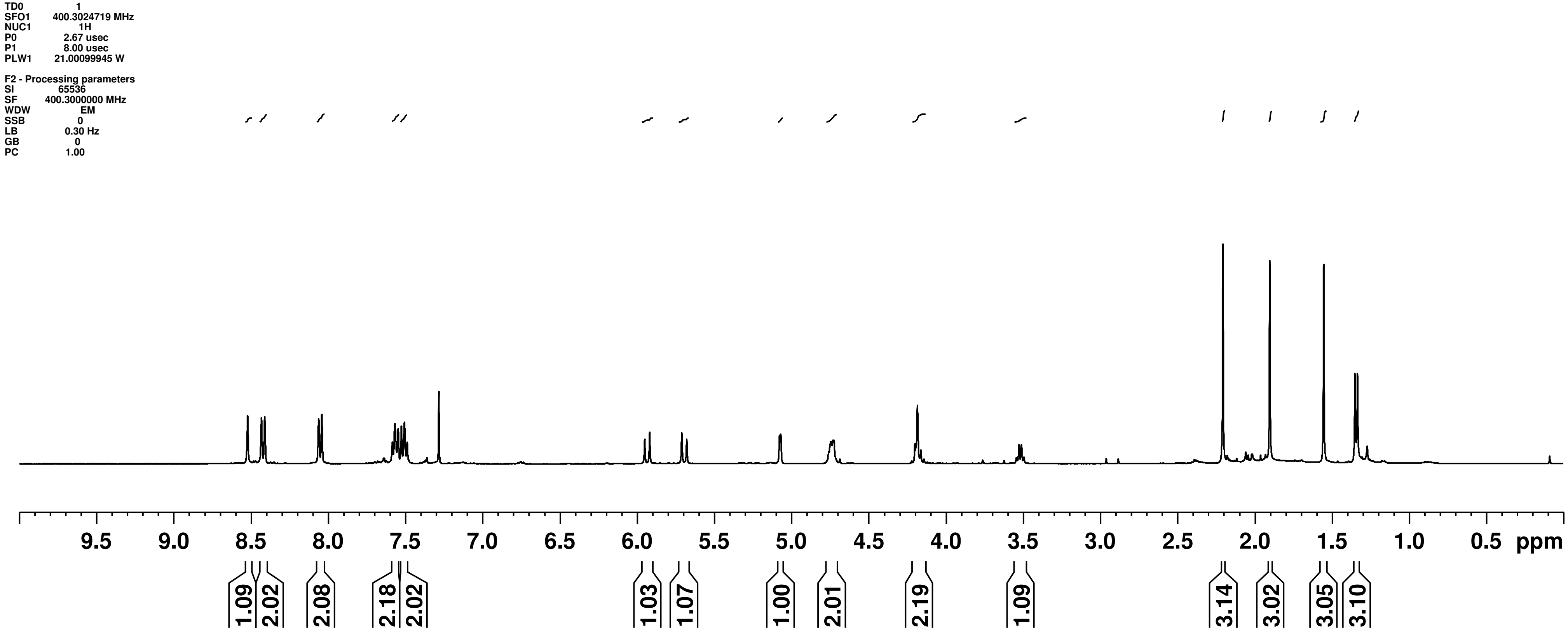

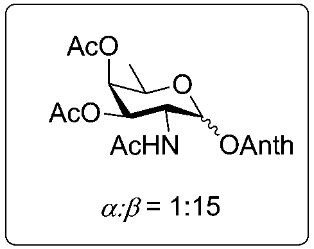

11

SSK-32-SKM-FUC-ANTH-NHAC-13C

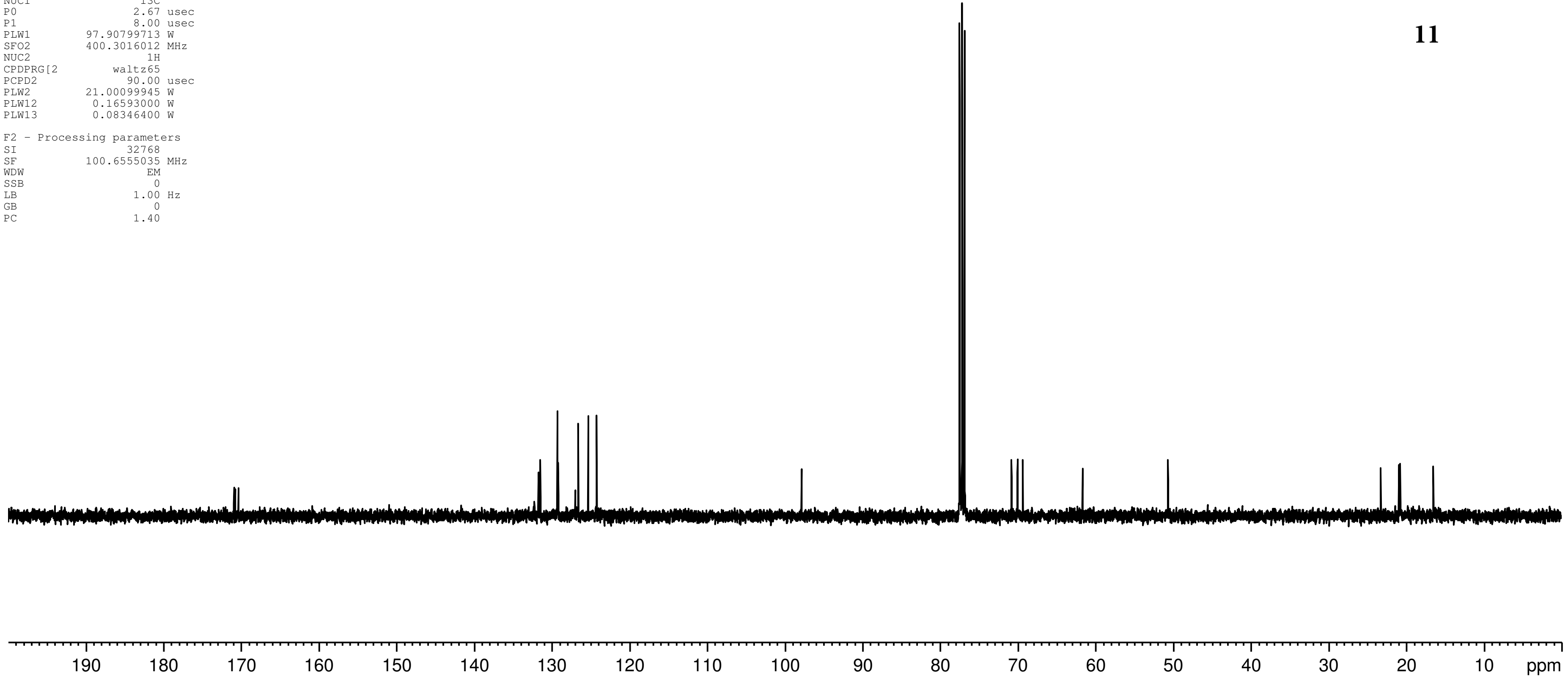


```
Current Data Parameters
NAME     SSK-32-SKM-FUC-ANTH-NHAC-13C
EXPNO                   5
PROCNO                  1

F2 - Acquisition Parameters
Date_            20240520
Time                 8.57 h
INSTRUM            Avance
PROBHD   Z163739_0237 (
PULPROG           zgpg30
TD                 65536
SOLVENT            CDCl3
NS                    78
DS                     0
SWH            27777.778 Hz
FIDRES          0.847710 Hz
AQ             1.1796480 sec
RG                   101
DW                18.000 usec
DE                  6.50 usec
TE                 297.1 K
D1            1.00000000 sec
D11           0.03000000 sec
TD0                    1
SFO1         100.6669898 MHz
NUC1                 13C
P0                  2.67 usec
P1                  8.00 usec
PLW1         97.90799713 W
SFO2         400.3016012 MHz
NUC2                  1H
CPDPRG[2         waltz65
PCPD2              90.00 usec
PLW2         21.00099945 W
PLW12         0.16593000 W
PLW13         0.08346400 W

F2 - Processing parameters
SI                 32768
SF           100.6555035 MHz
WDW                   EM
SSB                    0
LB                  1.00 Hz
GB                     0
PC                  1.40
```

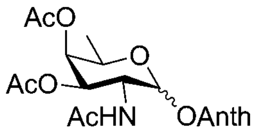

α:β = 1:15

11

SSK-32-SKM-FUC-ANTH-NHAC-DEPT

```
Current Data Parameters
NAME     SSK-32-SKM-FUC-ANTH-NHAC-DEPT
EXPNO              5
PROCNO             1

F2 - Acquisition Parameters
Date_          20240520
Time               9.00 h
INSTRUM          Avance
PROBHD   Z163739_0237 (
PULPROG       deptsp135
TD                65536
SOLVENT           CDCl3
NS                   56
DS                    0
SWH           27777.778 Hz
FIDRES         0.847710 Hz
AQ            1.1796480 sec
RG                  101
DW               18.000 usec
DE                 6.50 usec
TE                297.1 K
CNST2       145.0000000
D1           1.00000000 sec
D2           0.00344828 sec
D12          0.00002000 sec
TD0                   1
SFO1        100.6669898 MHz
NUC1                13C
P1                 8.00 usec
P13             2000.00 usec
PLW0                  0 W
PLW1        97.90799713 W
SPNAM[5]   Crp60comp.4
SPOAL5            0.500
SPOFFS5               0 Hz
SPW5         9.57390022 W
SFO2        400.3016012 MHz
NUC2                 1H
CPDPRG[2        waltz65
P3                 8.00 usec
P4                16.00 usec
PCPD2             90.00 usec
PLW2        21.00099945 W
PLW12        0.16593000 W

F2 - Processing parameters
SI                32768
SF          100.6555044 MHz
WDW                  EM
SSB                   0
LB                 1.00 Hz
GB                    0
PC                 1.40
```

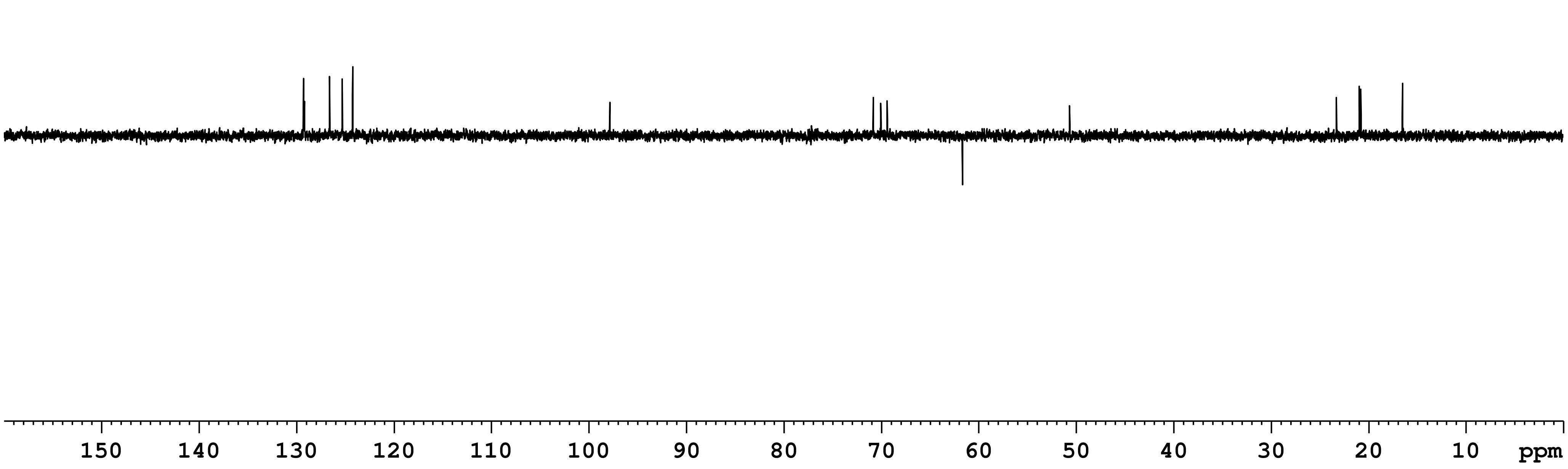

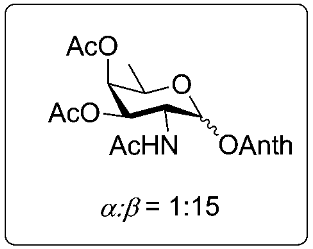

11

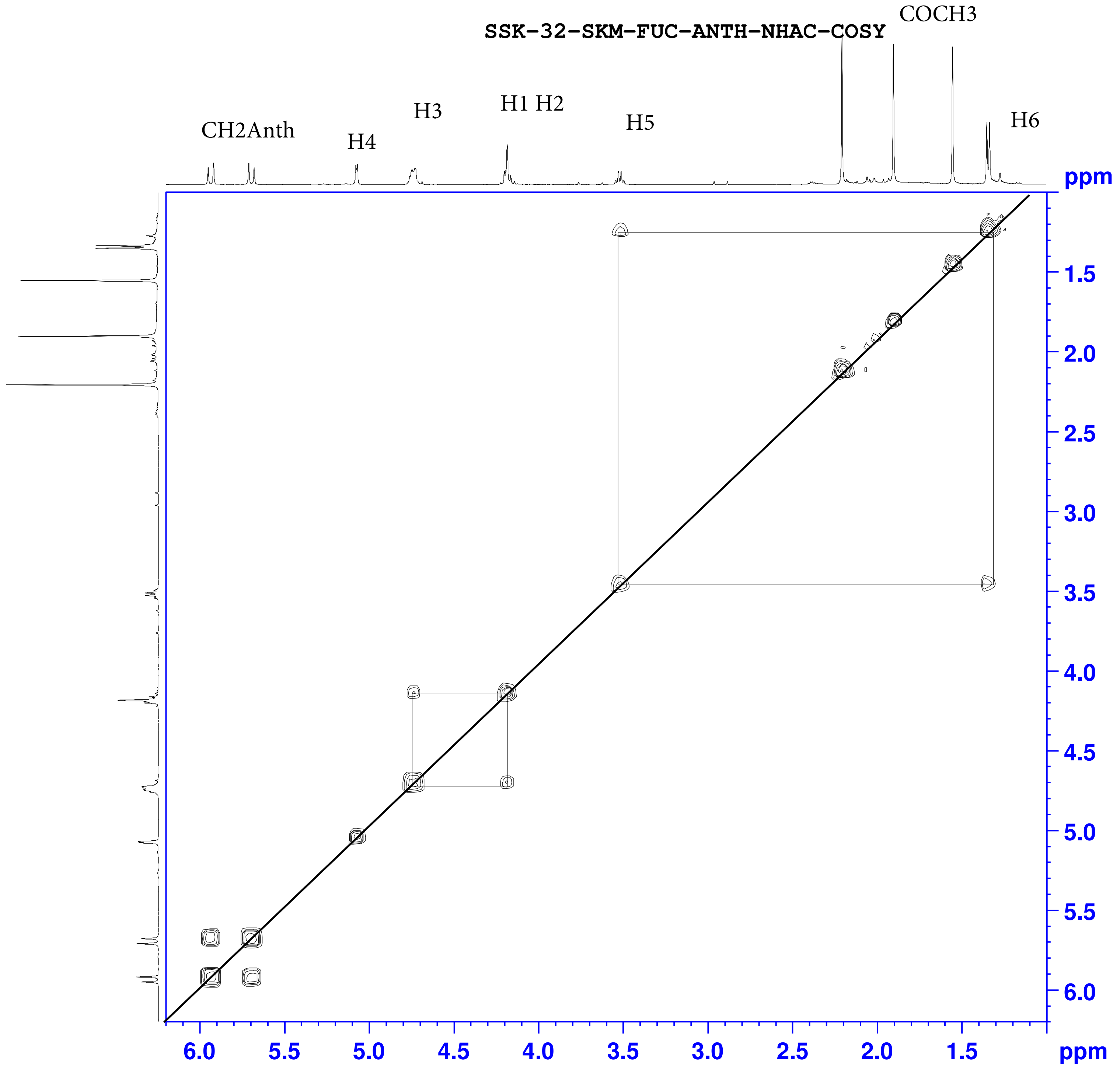

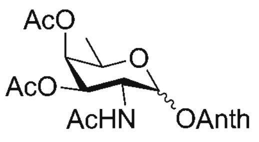

$\alpha:\beta$ = 1:15

11

```
Current Data Parameters
NAME       SSK-32-SKM-FUC-ANTH-NHAC-COSY
EXPNO                    10
PROCNO                    1

F2 - Acquisition Parameters
Date_              20240520
Time                   9.28 h
INSTRUM              Avance
PROBHD   Z163739_0237 (
PULPROG          cosygpppqf
TD                     2048
SOLVENT               CDCl3
NS                        4
DS                        0
SWH                8620.690 Hz
FIDRES             8.418642 Hz
AQ                0.1187840 sec
RG                       64
DW                   58.000 usec
DE                     6.50 usec
TE                    297.0 K
D0               0.00000300 sec
D1               1.00000000 sec
D11              0.03000000 sec
D12              0.00002000 sec
D13              0.00000400 sec
D16              0.00020000 sec
IN0              0.00011355 sec
TDav                      1
SFO1            400.3024018 MHz
NUC1                     1H
P0                     8.00 usec
P1                     8.00 usec
P17                 2500.00 usec
PLW1            21.00099945 W
PLW10            1.49339998 W
GPNAM[1]         SMSQ10.100
GPZ1                  10.00 %
P16                 1000.00 usec

======== F1 INDIRECT DIMENSION ========
td1        128
sw_F1      22.000000

F1 - Acquisition parameters
TD                      128
SFO1               400.3024 MHz
FIDRES           137.603958 Hz
SW                   22.000 ppm
FnMODE                   QF

F2 - Processing parameters
SI                     1024
SF              400.3000000 MHz
WDW                   QSINE
SSB                       0
LB                        0 Hz
GB                        0
PC                     1.40

F1 - Processing parameters
SI                     1024
MC2                      QF
SF              400.3000000 MHz
WDW                   QSINE
SSB                       0
LB                        0 Hz
GB                        0
```

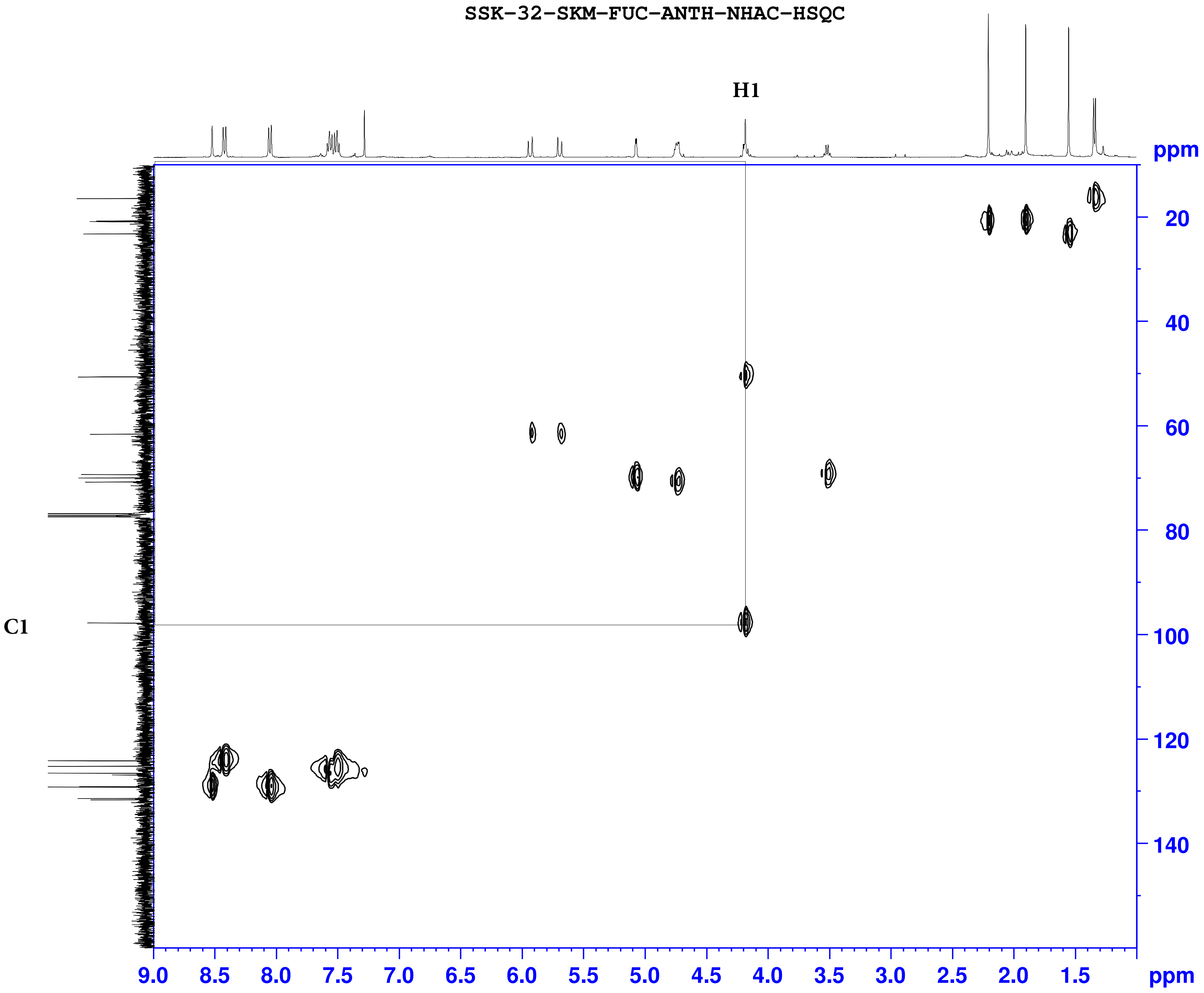

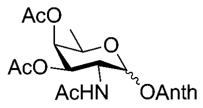

$\alpha:\beta$ = 1:15

11

```
Current Data Parameters
NAME      SSK-32-SKM-FUC-ANTH-NHAC-HSQC
EXPNO                  6
PROCNO                 1

F2 - Acquisition Parameters
Date_           20240520
Time                9.02 h
INSTRUM           Avance
PROBHD    Z163739_0237 (
PULPROG   hsqcedetgpsisp2.3
TD                  2048
SOLVENT            CDCl3
NS                     2
DS                     0
SWH             8196.721 Hz
FIDRES          8.004611 Hz
AQ             0.1249280 sec
RG                   101
DW                61.000 usec
DE                  6.50 usec
TE                 297.0 K
CNST2        145.0000000
CNST17        -0.5000000
D0            0.00000300 sec
D1            1.00000000 sec
D4            0.00172414 sec
D11           0.03000000 sec
D16           0.00020000 sec
D21           0.00344800 sec
D24           0.00086200 sec
IN0           0.00001799 sec
TDav                   1
ZGOPTNS
SFO1         400.3024719 MHz
NUC1                  1H
P1                  8.00 usec
P2                 16.00 usec
PLW1         21.00099945 W
SFO2         100.6669898 MHz
NUC2                 13C
CPDPRG[2           garp4
P3                  8.00 usec
P14               500.00 usec
P24              2000.00 usec
P31              2119.00 usec
PCPD2              80.00 usec
PLW0                   0 W
PLW2         97.90799713 W
PLW12         0.97908002 W
SPNAM[3] Crp60,0.5,20.1
SPOAL3             0.500
SPOFFS3                0 Hz
SPW3          9.57390022 W
SPNAM[7]     Crp60comp.4
SPOAL7             0.500
SPOFFS7                0 Hz
SPW7          9.57390022 W
SPNAM[18  Crp60_xfilt.2
SPOAL18            0.500
SPOFFS18               0 Hz
SPW18         1.84440005 W
GPNAM[1]      SMSQ10.100
GPZ1               80.00 %
GPNAM[2]      SMSQ10.100
GPZ2               20.10 %
GPNAM[3]      SMSQ10.100
GPZ3               11.00 %
GPNAM[4]      SMSQ10.100
GPZ4               -5.00 %
P16              1000.00 usec
P19               600.00 usec

======== F1 INDIRECT DIMENSION ========
td1       1024
sw_F1     276.000000

F1 - Acquisition parameters
TD                    72
SFO1             100.667 MHz
FIDRES        771.780273 Hz
SW               276.000 ppm
FnMODE    Echo-Antiecho

F2 - Processing parameters
SI                  1024
SF           400.3000000 MHz
WDW                QSINE
SSB                    2
LB                     0 Hz
GB                     0
PC                  1.40

F1 - Processing parameters
SI                  1024
MC2       echo-antiecho
SF           100.6555151 MHz
WDW                QSINE
SSB                    2
LB                     0 Hz
GB                     0
```
